# Socio-health factors, ability to perform instrumental and basic activities of daily living, and use of assistive mobility devices during the COVID-19 pandemic: Interrelationships and impact on long-term survival

**DOI:** 10.1371/journal.pone.0318481

**Published:** 2025-05-19

**Authors:** Vicente Martín Moreno, María Inmaculada Martínez Sanz, Irene Sánchez González, Miguel Recuero Vázquez, Sara Guerra Maroto, Miriam Fernández Gallardo, Amanda Martín Fernández, Julia Herranz Hernando, María Palma Benítez Calderón, Elena Pérez Rico, Laura Calderón Jiménez, Elena Sánchez Rodríguez, Helena Alonso Samperiz, Irene León Saiz, Juana Marcos Guerra

**Affiliations:** 1 Orcasitas Health Care Center, Madrid, Spain; 2 i+12 Research Institute of the Doce de Octubre hospital; GIDO collaborative group codirector, Madrid, Spain; 3 GIDO Collaborative Group Codirector, Madrid, Spain; 4 Nursing Home Care Unit of the Center Assistance Directorate, Madrid, Spain; 5 Polibea Concert; GIDO collaborative group, Madrid, Spain; King Faisal University, SAUDI ARABIA

## Abstract

**Introduction:**

Functional dependence for the performance of basic activities of daily living (ADLs) is one of the main causes of institutionalization. This study analyzed the interrelationships between basic and instrumental activities of daily living, use of assistive mobility devices, socioeconomic factors, changes during COVID-19 pandemic confinement, and 3-year survival in the ADL-dependent people of the Orcasitas neighborhood of Madrid (Spain).

**Methods:**

A longitudinal descriptive study, carried out on the entire population of functional dependent patients (Barthel ≤ 60) in the Orcasitas neighborhood. We included 127 patients, 78.7% women and 21.3% men, with a mean age of 86 years. Pre-pandemic, post-confinement (June 2020) and June 2023 data were contrasted.

**Results:**

Results: The use of crutches-cane was associated with a higher probability of being independent in performing ADLs, leaving home (OR 4.848; CI 1.428–16.458), improving functional capacity during confinement (OR 3.621; CI 1.409–9.308), and even ceasing to be functionally dependent (OR 0.394; CI 0.165–0.941). Using a wheelchair was associated with a higher level of dependency (OR 2.583; CI 1.167–5.714) and higher mortality (HR 1.913; CI 1.106–3.309). After COVID-19 pandemic confinement, having a financial income of less than 11,200 euros/year (OR 2.413; CI 1.159–5.023), or using a wheelchair (OR 2.464; CI 1.009–6.017), increased the risk of living homebound. Living homebound decreased the probability of survival, while maintaining the ability to leave home increased it (OR 3.880; CI 1.834–8.211). Economic capacity modulated the results. Lower economic capacity was associated with higher mortality (HR 2.47 (Exp(B) 0.405; CI 0.232–0.708). Living in confinement and having a low economic income were associated with higher mortality (OR 0.127; CI 0.029–0.562), mortality that was also higher with respect to those who could leave their home (OR 6.697; CI 2.084–21.525).

**Conclusions:**

Functional ADL-dependence affects multiple facets of the person. Confinement triggered changes in the baseline conditions of this cohort, which were influenced by the level of dependency, mobility capacity and economic income level. Economic capacity modulated the results, showing that social inequalities influence survival. The ability to leave home and the use of a wheelchair should be included in the assessment of the risk level of this population group.

## Introduction

Longer life expectancy in recent decades and the low birth rate have transformed the classic demographic patterns of our society, creating new realities, among them, that of population ageing. This situation has generated new needs and new objectives, including the promotion of healthy ageing. The presence of an ever-increasing number of older people brings with it the need to address the functional limitations associated with the ageing process itself. These limitations, associated with comorbidities, the possible loss of a partner and, sometimes, loneliness or social isolation, contribute to the development of functional dependence for the carrying out of basic activities of daily living (ADLs) [1], which is one of the main causes of institutionalization [2,3].

After ADL-dependency is established, patients can follow two evolutionary itineraries, either to remain at home or become institutionalized in a nursing home. These two alternatives have different trajectories. Institutionalized patients receive scheduled daily follow-up and rehabilitative or palliative measures for their dependency. However, non-institutionalized patients have less access to these measures and do not receive daily follow-up, marking a difference that could lead to inequalities [4]. On the other hand, media interest focuses on the first group [5], contributing to the relative invisibility of non-institutionalized dependent patients, reflected in a smaller number of studies in this population [6].

This situation contributes to a lesser knowledge of functional dependence in the community-dwelling population. This adds to the limited evidence on what level of independence to perform instrumental activities persists within the dependence to perform basic activities of daily living, data that have relevance in health planning [7–9]. Because personal autonomy depends largely on the independence to perform these activities, and the level of mobility preserved.

Moreover, the functional limitations inherent to the aging process can lead to a reduction in living space, to the point of reducing it to the home. This functional impairment decreases the quality of life, but it is not an isolated process, it occurs in an environment, which also influences its evolution. Spain is one of the countries where disability is most prevalent, with its prevalence increasing every year [10], a situation that generates a growing demand for care [9].

Autonomy, capabilities and mobility that often require the use of mobility assistance devices [11]. The need for these mobility assistance devices has been associated with the characteristics of the dwelling in which one resides, the economic capacity, the social situation of living alone or living with other people, the burden of morbidity and the limitations associated with age, which contribute to limiting the ability to perform basic and instrumental activities of daily living [12]. On the other hand, its use reduces the caregiver’s burden [13].

However, the extent to which reliance on the use of these devices influences survival is not well known. There also remains some degree of uncertainty as to what factors influence the availability of these devices [14]. Among these factors, economic capacity may be relevant in certain settings. There is also no clear evidence on how the use of these devices affects survival itself.

In parallel, other aspects must be present as well. Mobility assistance devices contribute to maintaining autonomy, but they also make dependence visible and contribute to creating stereotypes that favor ageism in society. Perhaps for these reasons, some older people renounce their use, even though they need them [15]. However, the perceptions of older people towards the use of mobility aids have been little explored [16] and survival itself.

In the current context, other factors must also be considered, such as the COVID-19 pandemic, which implied prolonged confinement to the home in many countries, and which had such a significant impact on the older people in our environment as to change behavioral patterns, generating a “post-COVID society” [17], changes whose influence on survival needs to be analyzed.

Based on these premises, several factors need to be analyzed. On the one hand, the use of mobility assistance devices probably implied a greater functional deterioration, and this deterioration probably had a gradient, such that not using these devices would imply a better functional situation, while using crutches-cane would indicate a lesser deterioration than using a walker and using a walker a lesser deterioration than needing a wheelchair. This different level of functional impairment suggested by having to require the use of one or the other mobility assistance device could influence survival, and this effect can be indirectly estimated through the device used. This is a relevant issue, as there is a high degree of uncertainty about these aspects, especially in people with greater functional impairment [18,19]. Also, because the increase in the use of this type of device in recent decades justifies the evaluation of its effectiveness [20].

On the other hand, beyond the need for the use of assistive devices, mobility itself, assessed dichotomously by the indicators “having the ability to leave the house” and “living homebound”, also shows the impact of functional impairment. The community-dwelling mode, open to the outside or confined to the home, could promote social fragility [21] that could result in different mortality rates depending on the housing situation [22].

Both ways of assessing mobility are likely to be modulated, in turn, by economic status, which may play a role, since a higher economic status may make it easier to acquire mobility aid or to leave the home. Economic capacity has been associated with a higher probability of living homebound [19,23] and living homebound with higher mortality [22]. Thus, economic capacity would play a role in the survival of these individuals.

At the same time, and influenced by economic capacity, the presence of a greater degree of functional dependence, both basic and instrumental, was probably associated with a greater need for assistants for housework and personal care, or for an internal caregiver. Also, of the need for nursing care [24].

Finally, the different functional capacity would also imply a different ability to perform both basic activities of daily living and instrumental activities of daily living, which could be influenced by the economic level of the population [25]. Being less able to perform these activities probably increased the frailty of these persons [26] and could influence their survival [27]. And both factors, greater frailty and a higher level of dependency, are associated with higher mortality [13,27].

In addition to the economic level and the mentioned factors, multimorbidity also plays a relevant role in the development of disability, as has been reflected in several studies, being an indispensable factor when analyzing the interrelationships that lead to functional dependence [11].

Within morbidity, finally, the COVID-19 pandemic had a severe impact on the world population and modified social behavior patterns [28,29], having an effect both on the baseline situation of this population and on the factors that traditionally coexisted with it, an effect that should be analyzed.

Autonomy depends on many factors, and the functional capacity to perform activities and mobility largely condition the outcomes. Knowledge of all these facets can allow for more personalized care and more efficient resource management.

The present study focused on the population dependent for the performance of ADLs in the Orcasitas neighborhood of Madrid (Spain) and was aimed at answering these hypotheses. The objectives of this study are as follows: 1: To analyze the influence of the level of dependence to perform basic activities of daily living on the ability to perform instrumental activities of daily living and on survival. 2: To analyze in relation to personal autonomy the interrelationships between functional capacity to perform basic and instrumental activities of daily living and mobility. 3: to analyze the influence of mobility on long-term survival, evaluating mobility by means of three indicators: a: the need to use mobility assistance devices; b: the need for assistants for housework. And c: mode of community dwelling, contrasting the situation of having the ability to leave the house versus living homebound. 4: to analyze the association between disease burden, the housing situation of living homebound and survival. 5: to analyze the interrelationships between these indicators, disease burden, polypharmacy, level of dependency and economic status. 6: finally, to analyze in parallel the influence of the COVID-19 pandemic on these indicators and on survival.

## Material and methods

### Design

Prospective longitudinal descriptive study conducted in the non-institutionalized functional dependent population of the Orcasitas neighborhood of Madrid (Spain) between June 2020 and August 2023. The study was carried out on the whole population with functional dependence for the performance of basic activities of daily living residing in the Orcasitas neighborhood, 150 persons, a population group that constituted the Orcasitas cohort. The data for this population were obtained from the informatics applications of the Madrid Health Service.

The Orcasitas neighborhood is situated in the south of the city of Madrid and is a socioeconomically disadvantaged neighborhood. Orcasitas has a single health center, which provides its health services to this population of 22,452 people as of June 2020.

The nationwide lockdown for COVID-19 began on March 15, 2020, and Madrid passed to phase 1 of the de-escalation on May 18, 2020. The state of alarm across Spain ended on June 21, 2020. In phase 1, de-escalation allowed people to leave for noncompulsory activities within time limits. We established three points of analysis: before confinement, end of national confinement in June 2020 and June 2023.

### Study population

All patients aged 65 years or older with a Barthel index score equal to or less than 60 recorded in the last year (May 2019-April 2020) in the informatics platform of the Orcasitas health center were included. These records were obtained in April 2020. During the months of May and June 2020, the population that met these criteria was contacted, making an appointment to invite them to participate in the study and explain its content. Fieldwork was carried out in June and the first week of July 2020.

Exclusion criteria were not being at home during the study period (n = 9) and the absence of pre-confinement Barthel score (n = 5). Five patients refused to participate in the study, four died in May-June 2020, before starting the fieldwork and 14 were excluded for the following causes: not located (n = 1), being in another home (n = 2), admission to a nursing home (n = 4), admission to hospital (n = 2), wrong previous Barthel (n = 1) and not having a Barthel in the previous year (n = 4). Finally, 127 patients participated, of whom 100 were women and 27 men.

### Variables

Through a questionnaire, we gathered each participant’s age; sex; marital status; educational level; family characteristics; availability of public, private, or internal caregivers, and changes in these caregivers because of the pandemic. The use of mobility assistance devices was also recorded: crutches-cane, walker, and wheelchair.

The nurses who collected the data using these questionnaires in June 2020 received prior training to unify the information transmitted to the participants and the possible answers to their questions. They also did not know the pre-pandemic level of functional dependency of the participants.

The educational level was classified according to the Madrid City Council protocol [30]: 1: Insufficient education (includes “Illiterate”, “Uneducated”, and “Incomplete primary education”). 2: Primary education (includes elementary baccalaureate degree and professional training certification). 3: Middle education (high school). 4: Higher education.

To answer the objectives of the study, personal autonomy was assessed by means of functional capacity and mobility indicators. Functional capacity indicators were the ability to perform basic activities of daily living at home and the ability to perform instrumental activities outside the home. Mobility assessment was based on three indicators: 1: use of mobility assistance devices: crutches-cane, walker or wheelchair. 2: having a public or private home assistant or internal caregiver assigned. 3: maintaining the ability to leave the house for walks or activities, as opposed to living homebound.

In relation to capacities, functional capacity for the performance of basic activities of daily living (ADLs) was assessed using the Barthel index, a widely validated tool, establishing a cut-off point of a Barthel index score of 60 to define functional dependence, in accordance with the protocols of the Madrid Health Service [31].

On the other hand, the functional capacity to perform instrumental activities of daily living was assessed by the following activities: 1: Leaving the house; 2: How the patient leaves the house (alone, accompanied); 3: Leaving the house for non-compulsory activities (walking, leisure); 4: Shopping autonomously; 5: Supervised shopping; and 6: Shopping by others. These activities were evaluated pre- and post-confinement by the COVID-19 pandemic.

Autonomy was also assessed by recording the need to use mobility assistance devices: wheelchair, walker, crutches/cane.

The economic level as of June 2020 was also obtained through the software application Individual Health Insurance Card (IHIC), of the Health System of the Community of Madrid, which reflects data from the Ministry of Finance.

The date of death of the patients included in this study was obtained using the software applications of the Health System of the community of Madrid IHIC and Cibeles. Blinding techniques were applied, so that the professional who recorded the date of death did not have access to the data obtained from the surveys and medical records, while the professionals who handled the database in which these data were incorporated did not have access to the computer systems that recorded the date of death from the central services.

Finally, through the AP-Madrid application, were registered the disease contemplated in the Strategy of Care for People with Chronic Diseases (EAPEC) [32] of the Community of Madrid (diabetes mellitus, chronic obstructive pulmonary disease [COPD]; cerebrovascular accident; arterial hypertension; ischemic heart disease; heart failure; asthma; obesity; dyslipidemia; renal insufficiency). The number of chronic diseases and the number of drugs consumed by the patient were also obtained from this informatics application.

### Data analysis

#### Variables utilization criteria.

**Fig 1:**
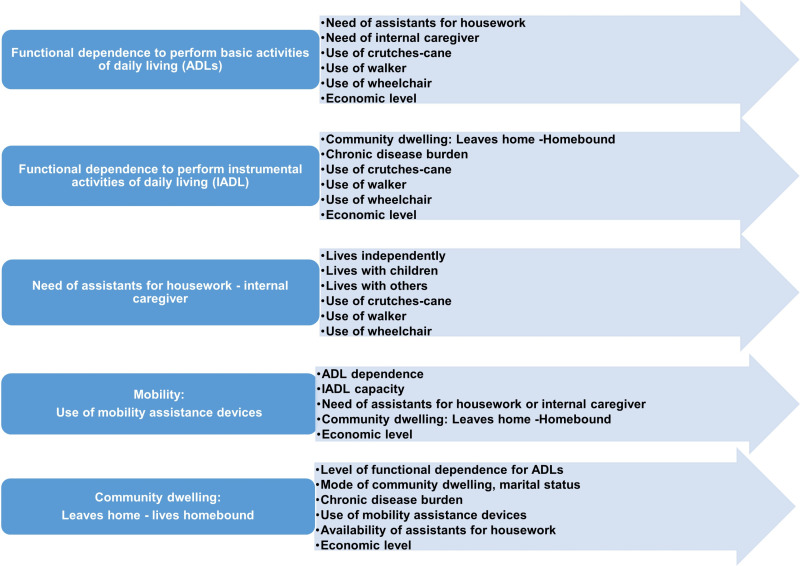
Autonomy, COVID-19 pandemic confinement and survival. Interrelationships. Representative diagram of the analysis of the interrelationships between the variables associated with functional dependence and the variables directly or indirectly associated with mobility. The influence of confinement to the usual home due to the COVID-19 pandemic was also analyzed for each node.

Fig 1 shows the main variables used and the scheme of interrelationships between them analyzed to meet the objectives of this study.

The classic classification of the Barthel index has four dependency levels: total (Barthel <20), severe (Barthel 20–35), moderate (Barthel 40–55) and mild (Barthel 60). However, in this study a dichotomized Barthel index classification was used, with two levels of functional dependence, severe (Barthel <40), which includes the total and severe levels of the classic classification, and moderate (Barthel 40–60), which includes the moderate and mild levels of the classic classification. This criterion is included in the “Plan for attention to frailty and promotion of healthy longevity in the older persons” of the Madrid Health Service, a document that reflects that 5-year mortality is 75% when there is a moderate level of functional dependence and 78.8% when there is a severe level [31].

An adaptation of the Barthel index classification to the classification proposed by Granger [33] was also used in some analyses, with two categories: activities associated with mobility (chair-to-bed transfer, going up-down stairs, walking and dressing-undressing) and activities not associated with mobility (the rest of the activities of the Barthel index).

In parallel, the different activities included in the Barthel index were contrasted with the rest of the variables under study. The activities of the Barthel index include two, three or four levels of functional capacity, depending on the activity. Therefore, in addition to using this classic classification criterion, a contrast was made with the rest of the variables and survival by dichotomizing each activity of the Barthel index into two response levels: “independent or needing minimal help” and “dependent or needing great help”. This criterion was adopted with the aim of giving greater equivalence to the “moderate” and “severe” levels of the global score obtained by means of the Barthel index.

The socio-health factors were operationalized by the ability to perform basic and instrumental activities of daily living, the assignment of assistants for housework or an internal caregiver, the mode of living in the community, and the need to use mobility assistance devices. On the other hand, confinement due to the COVID-19 pandemic was another socio-health factor analyzed.

Regarding the economic level of the population, the Madrid Health Service establishes that having an economic income of less than 11,200 euros/year is a criterion of economic vulnerability, which justifies that people with this income receive free pharmaceutical benefits. This economic threshold was used in this study as a criterion of low economic income.

The follow-up period for the Orcasitas cohort was three years (June 2020 - June 2023). This period was subdivided into 6-month time periods, with cut-off points in June and December of each year.

The impact of the COVID-19 pandemic was analyzed using the changes in the variables under study between their values before confinement due to the COVID-19 pandemic and the values observed after the end of confinement. The analyses were performed according to educational level, level of dependency and level of economic income. For each variable, we compared its impact on the population that was presented with that factor versus the population that did not present it. Previously published studies have analyzed the impact of confinement in relation to the level of functional dependence and sex [34], as well as in relation to the model of care of dependent persons and the mode of residence in the community [35].

Regarding missing cases, failure to answer four or more survey questions (10%) was considered equivalent to being excluded from the study. When the number of unanswered questions within the same questionnaire, or missing data within the same participant, was less than four, the method used was discarded by pairwise deletion.

The interrelationships between the variables were plotted using the Gephi Graph^®^ 0.10.1 program.

#### Descriptive analyses.

A descriptive analysis of the data obtained was carried out using the SPSS 18.0® statistical package, using mean and standard deviation for continuous variables, and using numbers/proportions for categorical variables.

The normality of the data distribution was checked via the Shapiro‒Wilk test. Given the small sample size in the male group, less than 30, to reconfirm this equality of variances ANOVA was performed with Levene’s test of homogeneity, applying Welch’s correction as a robust test of equality of means. In this study, a value of p<0.05 was considered significant.

#### Bivariate analysis.

The SPSS^®^ 18.0 statistical package was used for the bivariate analysis. Differences between continuous variables were analyzed via Student’s t test or the Mann‒Whitney U test, and differences between categorical variables were analyzed via the chi‒square test. The probability of the occurrence of an event was analyzed via odds ratios (ORs).

In addition to this analysis, a bivariate analysis was performed with all the variables included in this study to identify those that were associated with long-term survival. Variables that showed significant association (p<0.05) were included in the survival analysis via Cox regression and a proportional hazards model. The resulting model was summarized using the estimated coefficients, p-values and Hazard Ratios, with their 95% confidence intervals.

#### Post-hoc analysis.

Post-hoc tests were performed by linear regression analysis, stepwise model, to establish the percentages of variance in relation to survival explained by each variable, and predictive models were developed based on these results.

### Ethics statement

All participants signed an informed consent form and were previously informed of the objectives of the study and the publication of the results. The database was anonymized prior to data analysis. To guarantee the confidentiality of the data and records, the regulations included in the Spanish Data Protection Law 3/2018 and the Declaration of Helsinki, 2013 version, in effect in 2020, were followed.

This study was approved by the Local Research Commission of the Assistance Directorate Centre dependent on the Primary Care Management of the Department of Health of the Community of Madrid (Spain), resolutions14/20-C, of June 11, 2020, and 16/20-C-Bis, of June 29, 2020. The Ethics Committee of Hospital Universitario Doce de Octubre endorsed this as sufficient approval, resolution 23/501 of September 26, 2023.

## Results

### Sociodemographic data

The functional dependent population of the Orcasitas cohort had a mean age of 86.6 years, with 90.6% of the population in the 80 or older age group. Of the people in this cohort, 78.7% (n = 100) were women, mostly widowed, and 21.3% (n = 27) were men, most of whom were married (Table 1). In this cohort, 30.7% (n = 39) of the people lived with their partner. Analysis of variance showed no significant differences between men and women in relation to age, Barthel score, level of functional dependence, or BMI.

**Table 1:** Socio-health and demographic data of the functionally dependent population of Orcasitas (Orcasitas cohort) in June 2020.

Socio-demographic and socio-health data of the orcasitas cohort			
**Socio-demographic data**			
Variable/sex	Male	Woman	Total
n (%)	27 (21.3%)	100 (78.7%)	127 (100%)
Age (range):	(Rango 71–99)	(Rango 66–102)	(Rango 66–102)
65–74 years	1 (3.7%)	5 (5%)	6 (4.7%)
75–79 years	1 (3.7%)	5 (5%)	6 (4.7%)
≥80 years	25 (92.6%)	90 (90%)	115 (90.6%)
Mean ± SD	87.19 ± 5.962	86.48 ± 6.414	86.6 ± 6.304
Marital status:			
Married	15 (55.6%)	24 (24%)	39 (30.7%)
Widower	11 (40.7%)	71 (71%)	82 (64.6%)
Separated	1 (3.7%)	2 (2%)	3 (2.4%)
Single	0 (0%)	3 (3%)	3 (2.4%)
Schooling:			
Insufficient	23 (85.2%)	88 (88%)	111 (87.4%)
Primary	3 (11.1%)	7 (7%)	10 (7.9%)
Media	1 (3.7%)	3 (3%)	4 (3.1%)
Superiors	0 (0%)	1 (1%)	1 (0.8%)
Individual income level:			
<11,200 euros/year	13 (48.1%)	47 (47%)	60 (47.2%)
≥11,200 euros/year	14 (51.9%)	53 (53%)	67 (52.8%)
**Socio-health data**			
Barthel level/salary euros/year			
Severe/more than 11,200 euros	3 (11.1%)	11 (11%)	14 (11%)
Severe/less than 11,200 euros	5 (18.5%)	19 (19%)	24 (18.9%)
Moderate/more than 11,200 euros	11 (40.7%)	42 (42%)	53 (41.7%)
Moderate/less than 11,200 euros	8 (29.6%)	28 (28%)	36 (28.4%)
Public assistant:			
Yes	15 (55.6%)	55 (55.6%)	70 (55.6%)
No	12 (44.4%)	44 (44.4%)	56 (44.4%)
Private assistant:			
Yes	10 (38.5%)	35 (35.7%)	45 (36.3%)
No	16 (61.5%)	63 (64.3%)	79 (63.7%)
Internal caregiver:			
Yes	5 (18.5%)	19 (19.4%)	24 (19.2%)
No	22 (81.5%)	79 (80.6%)	101 (80.8%)
Wheelchair:			
Yes	11 (40.7%)	30 (30%)	41 (32.3%)
No	16 (59.3%)	70 (70%)	86 (67.7%)
Walker:			
Yes	8 (29.6%)	37 (37%)	45 (35.4%)
No	19 (70.4%)	63 (63%)	82 (64.6%)
Crutches-cane:			
Yes	14 (51.9%)	40 (40%)	54 (42.5%)
No	13 (48.1%)	60 (60%)	73 (57.5%)
Day center			
Yes	5 (18.5%)	16 (16.2%)	21 (16.5%)
No	22 (81.5%)	83 (83.8%)	105 (82.7%)
Telecare			
Yes	23 (85.2%)	73 (73.7%)	96 (75.6%)
No	4 (14.8%)	26 (26.3%)	30 (23.6%)
BMI (mean ± SD)	27.43 ± 3.684	28.90 ± 4.914	28.58 ± 4.701
Barthel score (mean ± SD)	42.96 ± 20.06	43.36 ± 18.83	43.28 ± 19.01
Barthel level:			
Severe (under 40)	8 (29.6%)	30 (30%)	38 (29.9%)
Moderate (40–60)	19 (70.4%)	70 (70%)	89 (70.1%)
Chronic diseases:			
0–2	3 (11.1%)	33 (33%)	36 (28.3%)
3–4	13 (48.2%)	44 (44%)	57 (44.9%)
5 or more	11 (40.7%)	23 (23%)	34 (26.8%)
Polypharmacy	26 (96.3%)	89 (89%)	115 (90.6%)

At the educational level, the level of studies in both sexes showed a low educational level, with 11.8% (n = 15) of the members of the Orcasitas cohort not knowing how to read or write, 25.2% (n = 32) declaring a lack of schooling but knowing how to read and write, and 50.4% not having completed primary education. The analysis of economic capacity showed that 47.2% had an economic income of less than 11,200 euros/year, while 52.8% had an annual income of more than this amount.

On the other hand, between June 1, 2020, and December 31, 2020, 6.3% (n = 8) of the individuals in this cohort died. An additional four persons had died between obtaining the database in May 2020 and the start of fieldwork in June 2020. At 12 months (June 2021) 20.5% (n = 26) had died, with death rates of 29.9% (n = 38) at 18 months, 34.6% (n = 44) at 24 months (June 2022), 38.6% (n = 49) at 30 months and 40.9% (n = 52) at three years (June 2023). Two out of three deaths in the study period occurred in the first 18 months of follow-up.

This study analyzes the interrelationships between various variables associated with autonomy, capacity and mobility, which is why, following the scheme presented in Fig 1, specific sections have been developed within the Results section, where these interrelationships are shown. Also, when appropriate, the influence of confinement due to the COVID-19 pandemic is included in the analysis of these interrelationships, as well as the effect of all these factors on survival:

#### The role of ADL and IADL interrelationships in autonomy and survival.

The ability to perform activities inside the home was assessed by the Barthel index, while the ability to perform activities outside the home was analyzed by the ability to leave the home and the performance of various activities outside the home.

When functional capacity at home was analyzed (Table 2), the need for help from another person for bathing, feeding and go up-down stairs were the main functional limitations observed, and they also frequently needed help for grooming and dressing/undressing. Regarding sphincter control, one in five dependent patients presented with fecal incontinence, and one in three patients presented with urinary incontinence. Being dependent for the performance of basic activities of daily living was associated with a lower probability of survival, except for the Barthel index activities “bladder sphincter control” and “anal sphincter control”.

**Table 2:** Barthel index and basic activities of daily living. Results of the evaluation performed in the Orcasitas cohort after the end of confinement due to the COVID-19 pandemic and survival analysis at three years of follow-up. 1: Analysis performed dichotomizing each activity of the Barthel index.

BARTHEL INDEX: BASIC ACTIVITIES OF DAILY LIFE ORCASITAS COHORT (June 2020)					
Barthel scale item	n	(%)	Barthel scale item	n	(%)
Feeding:			Dressing-undressing:		
Independent	21	13.4%	Independent	35	27.6%
Help	17	16.5%	Help	52	40.9%
Dependent	89	70.1%	Dependent	40	31.5%
Bathing:			Grooming:		
Independent	35	27.6%	Independent	66	52%
Help	92	72.4%	Help	61	48%
Toilet use:			Going up-down stairs:		
Independent	60	47.2%	Independent	16	12.6%
Help	44	34.6%	Help	32	25.2%
Dependent	23	18.1%	Dependent	79	62.2%
Urinary sphincter control:			Anal sphincter control:		
Continent	25	19.7%	Continent	74	58.3%
Occasional incontinent	58	45.7%	Occasional incontinent	27	21.3%
Incontinent	44	34.6%	Incontinent	26	20.5%
Chair-to-bed transfer:			Walking:		
Independent	30	23.6%	Independent	17	13.4%
Minimal help	44	34.6%	Help	84	66.1%
Great help	42	33.1%	Dependent	26	20.5%
Dependent	11	8.7%			
**Barthel Index (dichotomized) and survival in June 2023**					
**Variable**	**n**	**(%)**	**Deceased**	**Odds ratio**	**Confidence interval**
Feeding:					
Independent	89	70%	30 (33.7%)	0.370	**0.170–0.806**
Dependent or help	38	30%	22 (57.9%)		
Bathing:				0.261	**0.104–0.658**
Independent	35	27.6%	7 (20%)		
Help	92	72.4%	45 (48.9%)		
Toilet use:				0.232	**0.087–0.615**
Independent or Help	104	81.9%	36 (34.6%)		
Dependent	23	18.1%	16 (69.6%)		
Urinary sphincter control:				0.490	0.233–1.032
Continent or Occasional	83	65.4%	29 (34.9%)		
Incontinent					
Incontinent	44	34.6%	23 (52.3%)		
Chair-to-bed transfer:				0.209	**0.098–0.449**
Independent or Minimal help	74	58.3%	19 (25.7%)		
Great help or Dependent	53	41.7%	33 (62.3%)		
Dressing-undressing:				0.230	**0.104–0.508**
Independent or help	87	68.5%	26 (29.9%)		
Dependent	40	31.5%	26 (65%)		
Grooming:				0.394	**0.191–0.815**
Independent	66	52%	20 (30.3%)		
Help	61	48%	32 (52.5%)		
Going up-down stairs:				0.276	**0.123–0.616**
Independent or Help	48	37.8%	11 (22.95)		
Dependent	79	62.2%	41 (51.9%)		
Anal sphincter control:				0.424	0.176–1.019
Continent or Occasional incontinent	101	79.5%	37 (36.6%)		
Incontinent	26	20.5%	15 (57.7%)		
Walking:				0.346	**0.142–0.842**
Independent or Help	101	79.5%	36 (35.6%)		
Dependent	26	20.5%	16 (61.5%)		

On the other hand, we analyzed the interrelationships between these basic activities of daily living, which were performed at home, and basic instrumental activities, such as leaving home (Table 3). In this analysis, we observed that being dependent or requiring great help to perform the chair-to-bed transfer was associated with a lower probability of leaving home, both before (OR 0.266; CI 0.124–0.572) and after (OR 0.208; CI 0.097–0.445) confinement. Also, a greater dependence to perform the activity of up-down stairs was associated with a lower probability of leaving home, both before (OR 0.276; CI 0.118–0.644) and after (OR 0.240; CI 0.110–0.524) confinement.

**Table 3:** Interrelationships between the items of the Barthel Index and the ability to leave home before and after the nationwide COVID-19 lockdown.

Interrelation between instrumental activities and Barthel index items						
	Leaves home before confinement		OR (CI)	Leaves home after confinement		OR (CI)
Variable	YES	NO		YES	NO	
Walking on level surfaces						
Independent-minimal help	69(54.4%)	32(25.2%)	0.464(0.193–1.113)	57(44.9%)	44(34.6%)	0.409(0.166–1.004)
Does not walk - wheelchair	13(10.2%)	13(10.2%)		9(7.1%)	17(13.4%)	
Urinary incontinence:						
Yes	59(46.5%)	24(18.9%)	**0.446(0.209–0.951)**	47(37%)	36(28.3%)	0.582(0.278–1.217)
No	23(18.1%)	21(16.5%)		19(15%)	25(19.7%)	
Fecal incontinence:						
No	68(53.5%)	33(26%)	0.566(0.236–1.360)	58(45.7%)	43(33.8%)	**0.330 (0.131–0.828)**
Yes	14(11%)	12(9.5%)		8(6.3%)	18(14.2%)	
Toilet use:						
Independent	70(55.1%)	34(26.8%)	0.530(0.212–1.323)	60(47.3%)	44(34.6%)	**0.259(0.094–0.710)**
Dependent	12(9.5%)	11(8.6%)		6(4.7%)	17(13.4%)	
Up and down stairs: Independent-minimal help	39(30.7%)	9(7.1%)	**0.276(0.118–0.644)**	35(27.6%)	13(10.2%)	**0.240 (0.110–0.524)**
Dependent	43(33.9%)	36(28.3%)		31(24.4%)	48(37.8%)	
Bathing:						
Independent	47(37%)	19(15%)	0.544(0.261–1.136)	40(31.4%)	26(20.5%)	**0.483(0.238–0.980)**
Dependent	35(27.5%)	26(20.5%)		26(20.5%)	35(27.6%)	
Dressing-undressing:						
Independent-minimal help	61(48%)	26(20.5%)	0.471(0.218–1.019)	52(41%)	35(27.5%)	**0.362(0.166–0.789)**
Dependent	21(16.5%)	19(15%)		14(11%)	26(20.5%)	
Chair-to-bed transfers:						
Independent-minimal help	57(44.9%)	17(13.4%)	**0.266(0.124–0.572)**	50(39.4%)	24(18.9%)	**0.208(0.097–0.445)**
Dependent or great help	25(19.7%)	28(22%)		16(12.6%)	37(29.1%)	
Grooming:						
Independent	24(18.9%)	11(8.6%)	0.782(0.341–1.793)	19(15%)	16(12.6%)	0.880(0.403–1.920)
Dependent	58(45.7%)	34(26.8%)		47(37%)	45(35.4%)	
Feeding:						
Independent	59(46.5%)	30(23.6%)	0.780(0.356–1.709)	50(39.4%)	39(30.7%)	0.567(0.263–1.223)
Dependent-minimal help	23(18.1%)	15(11.8%)		16(12.6%)	22(17.3%)	

Likewise, persons during confinement were dependent for bathing (OR 0.483; CI 0.238–0.980), dressing-undressing (OR 0.362; CI 0.166–0.789), or toileting (OR 0.259; CI 0.094–0.710) were more limited in their ability to leave home after confinement than persons who were not dependent for these activities. The percentage of people dependent for these activities increased during confinement (Table 3).

These differences had an impact on survival. Home confinement due to the COVID-19 pandemic, which lasted 100 days in Spain, also played a role, a role that is shown in Table 4 for all the activities of the Barthel index.

**Table 4:** Influence of ability to perform basic activities of daily living and mode of living in the community on survival at 3 years. 1: Odds Ratio: association between ability to perform basic activities of daily living and survival in relation to ability to leave home before (OR upper line) and after (OR lower line) COVID-19 pandemic confinement. 2: Odds ratio: association between ability to perform basic activities of daily living and survival in relation to homebound status before (OR upper line) and after (OR lower line) COVID-19 pandemic confinement. 2: CI: Confidence Interval.

Barthel index activity	Ability to leave home, barthel’s index and survival					
	Leaves home					
	Before confinement		After confinement		OR^1^	CI^2^
	Alive	Dead	Alive	Dead		
Walking on level surfaces						
Independent-minimal help	47 (57.3%)	22 (26.8%)	42 (63.6%)	15 (22.7%)	0.546	0.164–1.817
Does not walk-wheelchair	7 (8.6%)	6 (7.3%)	7 (10.6%)	2 (3.1%)	1.250	0.233–6.696
Urinary incontinence:						
Continent or occasional	42 (51.2%)	17 (20.7%)	37 (56.1%)	10 (15.1%)	0.442	0.164–1.192
Incontinent	12 (14.7%)	11 (13.4%)	12 (18.2%)	7 (10.6%)	0.463	0.145–1.485
Fecal incontinence:						
No	49 (59.7%)	19 (23.2%)	46 (69.7%)	12 (18.2%)	**0.215**	**0.064–0.726**
Yes	5 (6.1%)	9 (11%)	3 (4.5%)	5 (7.6%)	**0.157**	**0.033–0.749**
Toilet use:						
Independent	52 (63.4%)	18 (22%)	48 (72.7%)	12 (18.2%)	**0.069**	**0.014–0.346**
Dependent	2 (2.4%)	10 (12.2%)	1 (1.5%)	5 (7.6%)	**0.050**	**0.005–0.469**
Up and down stairs:						
Independent-minimal help	32 (39%)	7 (8.6%)	29 (43.9%)	6 (9.1%)	**0.229**	**0.083–0.631**
Dependent	22 (26.8%)	21 (25.6%)	20 (30.3%)	11 (16.7%)	0.376	0.120–1.184
Bathing:						
Independent	21 (25.6%)	3 (3.7%)	18 (27.3%)	1 (1.5%)	**0.189**	**0.051–0.704**
Dependent	33 (40.2%)	25 (30.5%)	31 (47%)	16 (24.2%)	**0.108**	**0.013–0.881**
Dressing-undressing:						
Independent-minimal help	46 (56.1%)	15 (18.3%)	42 (63.6%)	10 (15.2%)	**0.201**	**0.070–0.577**
Dependent	8 (9.8%)	13 (15.8%)	7 (10.6%)	7 (10.6%)	**0.238**	**0.068–0.835**
Transfers bed-to-chair:						
Independent-minimal help	45 (54.8%)	12 (14.7%)	40 (60.6%)	10 (15.1%)	**0.150**	**0.053–0.423**
Dependent or great help	9 (11%)	16 (19.5%)	9 (13.7%)	7 (10.6%)	0.321	0.096–1.074
Grooming:						
Independent	36 (44%)	11 (13.4%)	32 (48.5%)	8 (12.1%)	**0.324**	**0.126–0.833**
Dependent	18 (21.9%)	17 (20.7%)	17 (25.7%)	9 (13.7%)	0.472	0.154–1.446
Feeding:						
Independent	43 (52.4%)	16 (19.5%)	39 (59%)	11 (16.7%)	**0.341**	**0.126–0.927**
Dependent-minimal help	11 (13.4%)	12 (14.7%)	10 (15.2%)	6 (9.1%)	0.470	0.140–1.582
**Barthel index activity**	**Lives homebound**					
	**Before confinement**		**After confinement**		**OR**	**CI**
	**Alive**	**Dead**	**Alive**	**Dead**		
Walking on level surfaces						
Independent-minimal help	18 (40%)	14 (31.1%)	23 (37.7%)	21 (34.4%)	0.233	0.054–1.012
Does not walk-wheelchair	3 (6.7%)	10 (22.2%)	3 (4.9%)	14 (23%)	**0.196**	**0.049–0.778**
Urinary incontinence:						
Continent or occasional	12 (26.7%)	12 (26.7%)	17 (27.9%)	19 (31.2%)	0.750	0.231–2.435
Incontinent	9 (20%)	12 (26.7%)	9 (14.7%)	16 (26.2%)	0.629	0.221–1.790
Fecal incontinence:						
Not, or occasional	15 (33.3%)	18 (40%)	18 (29.5%)	25 (41%)	1.200	0.320–4.505
Yes	6 (13.3%)	6 (13.3%)	8 (13.1%)	10 (16.4%)	1.111	0.366–3.370
Toilet use:						
Independent	16 (35.6%)	18 (40%)	20 (32.8%)	24 (39.3%)	0.938	0.240–3.669
Dependent	5 (11.1%)	6 (13.3%)	6 (9.8%)	11 (18.1%)	0.655	0.206–2.084
Up and down stairs:						
Independent-minimal help	5 (11.1%)	4 (8.9%)	8 (13.1%)	5 (8.2%)	0.640	0.147–2.783
Dependent	16 (35.6%)	20 (44.4%)	18 (29.5%)	30 (49.2%)	0.375	0.106–1.323
Bathing:						
Independent	7 (15.6%)	4 (8.9%)	10 (16.4%)	6 (9.8%)	0.400	0.098–1.631
Dependent	14 (31.1%)	20 (44.4%)	16 (26.2%)	29 (47.6%)	0.331	0.102–1.079
Dressing-undressing:						
Independent-minimal help	15 (33.3%)	11 (24.4%)	19 (31.2%)	16 (26.2%)	0.338	0.098–1.171
Dependent	6 (13.3%)	13 (29%)	7 (11.4%)	19 (39.2%)	**0.310**	**0.104–0.925**
Transfers bed-to-chair:						
Independent-minimal help	10 (22.2%)	7 (15.6%)	15 (24.6%)	9 (14.7%)	0.453	0.133–1.547
Dependent or great help	11 (24.4%)	17 (37.8%)	11 (18.1%)	26 (42.6%)	**0.254**	**0.086–0.7**52
Grooming:						
Independent	10 (22.2%)	9 (20%)	14 (22.9%)	12 (19.7%)	0.660	0.201–2.170
Dependent	11 (24.4%)	15 (33.3%)	12 (19.7%)	23 (37.7%)	0.447	0.158–1.265
Feeding:						
Independent	16 (35.6%)	14 (31.1%)	20 (32.8%)	19 (39.2%)	0.438	0.120–1.591
Dependent-minimal help	5 (11.1%)	10 (22.2%)	6 (9.8%)	16 (26.2%)	0.356	0.115–1.102

Before confinement, among people who maintained the ability to leave home, being independent in “up-down stairs” (OR 0.229; CI 0.083–0.631), “dressing-undressing” (OR 0.201; CI 0.070–0.577) and “performing chair-to-bed transfers” (OR 0.150; CI 0.053–0.423) was associated with a higher likelihood of survival (Table 4). So was being independent in the non-mobility-associated activities “bathing”, “grooming,” “bathroom use”, and “fecal incontinence”. However, among people living homebound, maintaining independence in the different activities of the Barthel index was not associated with a higher probability of survival.

After COVID-19 pandemic confinement, among those who maintained the ability to leave home, greater dependence on dressing-undressing, bathing, toilet use, or fecal incontinence was associated with a lower likelihood of survival. Difference due to confinement, which also had an effect among people living homebound, among whom lower survival was observed when dependent in the activities of walking, dressing-undressing or chair-to-bed transfers, an association that was not observed before confinement (Table 4).

In addition to analyzing individually the role played by each activity of the Barthel index in these interrelationships and in survival, their weight was also analyzed as a whole, by means of the level of functional dependence. This level was obtained by adding the scores obtained for each activity of the Barthel index, scores that reflected the level of dependence or independence for the performance of each activity. This determination made it possible to establish that before confinement due to the COVID-19 pandemic, 70.1% of the people in this cohort had moderate functional dependence (Barthel 40–60), and 29.9% had severe functional dependence (Barthel <40). Within the group of people living homebound, when assessing the level of dependence as a factor associated with living homebound, 55.6% (n = 25) had moderate dependence and 44.4% (n = 20) had severe dependence (Table 5).

**Table 5:** Changes in functional capacity during confinement due to the COVID-19 pandemic, evolutionary course in the three-year follow-up of the Orcasitas cohort and survival. 1: association between postconfinement Barthel level and three-year survival.

Effects of confinement on functional capacity and survival in relation to the mode of living in the community					
**Barthel index level before COVID-19 confinement**					
Way of cohabitation in the community before confinement	Severe Dependency (0–35)	Moderate Dependence (40–60)	Total	OR	CI
Leaves home	18 (14.2%)	64 (50.4%)	82 (64.6%)	**0.352**	**0.160–0.772**
Homebound	20 (15.7%)	25 (19.7%)	45 (35.4%)		
**Barthel index level after COVID-19 confinement**					
Way of cohabitation in the community after confinement	Severe Dependency (0–35)	Moderate Dependence (40–60)	Not dependent (>60)	Total	Statistical
Leaves home	11 (8.7%)	21 (16.5%)	34 (26.8%)	66 (60%)	**χ**^**2**^ **= 6.174;** **p = 0.034**
Homebound	22 (17.3%)	18 (14.2%)	21 (16.5%)	61 (40%)	
**Leaves home Barthel index level after COVID-19 confinement**					
Barthel before confinement	Severe Dependency (0–35)	Moderate Dependence (40–60)	Not dependent (>60)	Total	Statistical
Severe	9 (11%)	6 (7.3%)	3 (3.7%)	18 (22%)	**χ**^**2**^ **= 14.785;** **p = 0.001**
Moderate	8 (9.8%)	18 (21.9%)	38 (46.3%)	64 (78%)	
**Survival at three years among people leaves home after COVID-19 confinement** ^**1**^					
Survival	Severe Dependency (0–35)	Moderate Dependence (40–60)	Not dependent (>60)	Total	Statistical
Alive	5 (7.6%)	16 (24.2%)	28 (42.4%)	49 (74.2%)	χ^2^ = 5.978; p = 0.050
Deceased	6 (9.1%)	5 (7.6%)	6 (9.1%)	17 (25.7%)	
**Homebound Barthel index level after COVID-19 confinement**					
Barthel before confinement	Severe Dependency (0–35)	Moderate Dependence (40–60)	Not dependent (>60)	Total	Statistical
Severe	13 (28.9%)	6 (13.3%)	1 (2.2%)	20 (44.4%)	**χ**^**2**^ **= 16.787;** **p<0.001**
Moderate	3 (6.7%)	9 (20%)	13 (28.9%)	25 (55.6%)	
**Survival at three years among people living homebound after COVID-19 confinement** ^**1**^					
Survival	Severe Dependency (0–35)	Moderate Dependence (40–60)	Not dependent (>60)	Total	Statistical
Alive	7 (11.3%)	6 (9.6%)	13 (21%)	26 (42%)	χ^2^ = 4.878; p = 0.087
Deceased	15 (24.2%)	13 (21%)	8 (12.9%)	36 (58%)	
**Barthel index level at three-year follow-up (June 2023)**					
Way of cohabitation in the community after confinement	Severe Dependency (0–35)	Moderate Dependence (40–60)	Not dependent (>60)	Total	Statistical
Leaves home	8 (14.5%)	18 (32.7%)	8 (14.5%)	34 (61.8%)	χ^2^ = 0.250; p = 0.883
Homebound	6 (10.9%)	11 (20%)	4 (7.3%)	21 (38.2%)	
**Changes in functional capacity in the three-year period June-2020 to June-2023**					
Barthel after confinement (June 2020)	Severe Dependency (0–35)	Moderate Dependence (40–60)	Not dependent (>60)	Total	Likelihood ratio
Severe	7 (12.7%)	4 (7.3%)	0 (0%)	11 (20%)	**15.758;** **p = 0.006**
Moderate	4 (7.3%)	7 (12.7%)	2 (3.6%)	13 (23.6%)	
Not dependent	3 (5.5%)	18 (32.7%)	10 (18.2%)	31 (56.4%)	

However, after confinement, 30.7% (n = 39) were classified as having moderate functional ADL-dependence (Barthel 40–60), 26% (n = 33) as having severe dependence (Barthel <40) and 43.3% (n = 55) ceased to be functionally ADL-dependent (Barthel >60) (Table 5). During confinement a change occurred and people with moderate functional dependence were more likely that this improvement in their functional capacity allowed them to stop being functionally dependent (OR 11.408; CI 3.730–34.891). A total of 57.3% (n = 51) of people with moderate dependence and 10.5% (n = 4) of people with severe dependence ceased to be ADL-dependent after the pandemic.

Analyzing the interrelationships between this outcome, community living and survival, among those who, after confinement, maintained the ability to leave home (n = 66), improving functional capacity until they were no longer dependent (n = 34) did not imply a greater probability of survival (OR 0.409; CI 0.130–1.285). In the 2020–2023 period, 17.6% (n = 6) of persons who, after COVID-19 pandemic confinement, ceased to be dependent and remained able to leave home (n = 34), and 34.4% (n = 11) of persons who continued to leave home and remained functionally dependent (n = 32) died.

However, among those who were living homebound after confinement (n = 61), improving their functional capacity during confinement did imply a higher probability of survival (OR 0.296; CI 0.098–0.892). In this group, 38.1% (n = 8) of those who managed to improve their functional capacity during confinement for the COVID-19 pandemic and ceased to be dependent died during this period, compared to 67.5% (n = 27) among those who remained dependent (n = 40).

On the other hand, maintaining the capacity for the activity of leaving home after confinement did not imply having obtained a greater level of functional improvement during confinement (OR 0.494; CI 0.242–1.011).

**Fig 2:**
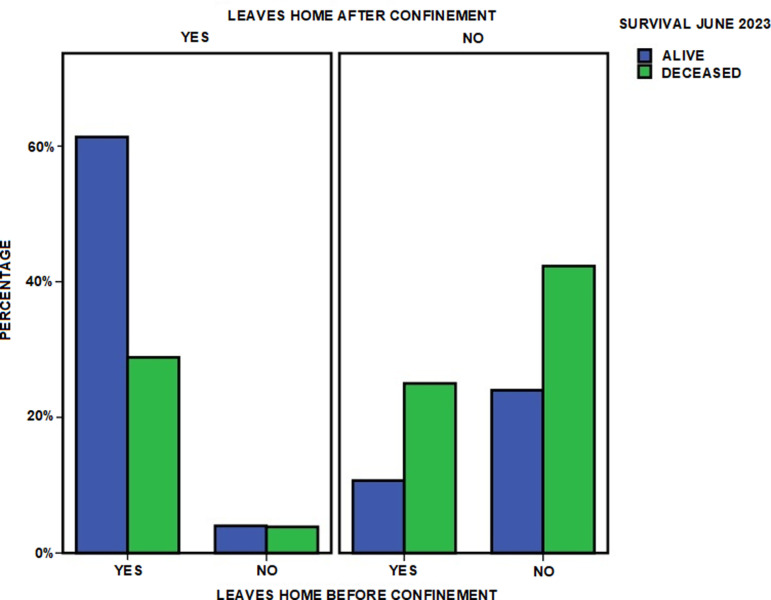
Influence of COVID-19 confinement on the ability to leave home and survival. Survival at three years of follow-up in relation to the changes produced during COVID-19 confinement in the mode of living in the community of persons with functional dependence in the Orcasitas cohort.

Independently of these changes due to confinement, having before confinement the ability to leave the house (OR 2.204; CI 1.049–4.631) and, above all, maintaining this ability after confinement (OR 3.880; CI 1.834–8.211), were associated with greater survival at three years (Table 6 and Fig 2).

**Table 6:** Changes in instrumental activities and autonomy during the nationwide COVID-19 lockdown and their relationships with the Barthel level, income level, and three-year survival.

Variable	Interrelation between instrumental activities, socioeconomic factors, and survival					
	Leaves home before confinement		OR (CI)	Leaves home after confinement		OR (CI)
	YES	NO		YES	NO	
Survival:						
Alive	54(42.5%)	21(16.5%)	**2.204** **(1.049–4.632)**	49(10.3%)	26(19.7%)	**3.880** **(1.834–8.211)**
Deceased	28(22.1%)	24(18.9%)		17(41.7%)	35(28.3%)	
**Variable**	**Leaves home before confinement**		**OR (CI)**	**Leaves home after confinement**		**OR (CI)**
	**YES**	**NO**	**YES**	**NO**		
Barthel level:						
Severy dependence	18(14.2%)	20(15.7%)	**0.352** **(0.160–0.772)**	13(10.3%)	25(19.7%)	**0.353** **(0.160–0.780)**
Moderate dependence	64(50.4%)	25(19.7%)		53(41.7%)	36(28.3%)	
Income level:						
Less 11,200 euros/year	36(28.3%)	24(19%)	0.685 (0.330–1.421)	23(18.1%)	37(29.1%)	**0.347** **(0.169–0.714)**
Greater 11,200 euros/year	46(36.2%)	21(16.5%)		43(33.8%)	24(19%)	
Survival June 2023:						
Alive	54(42.5%)	21(16.5%)	**2.204** **(1.049–4.631)**	49(38.6%)	26(20.5%)	**3.880** **(1.834–8.211)**
Deceased	28(22.1%)	24(18.9%)		17(13.4%)	35(27.5%)	
**Variable**	**Leaves home to walk before confinement**		**OR (CI)**	**Leaves home to walk after confinement**		**OR (CI)**
	**YES**	**NO**		**YES**	**NO**	
Barthel level:						
Severy dependence	14(11.6%)	22(8.2%)	**0.296** **(0.132–0.666)**	10(8.3%)	26(21.5%)	0.476 (0.204–1.108)
Moderate dependence	58(47.9%)	27(22.3%)		38(31.4%)	47(38.8%)	
Income level:						
Less 11,200 euros/year	33(27.3%)	24(19.8%)	0.881 (0.426–1.824)	17(14.1%)	41(33.9%)	**0.428** **(0.202–0.907)**
Greater 11,200 euros/year	39(32.2%)	25(20.7%)		31(25.6%)	32(26.4%)	
Survival June 2023:						
Alive	47(38.8%)	26(21.5%)	1.663 (0.792–3.492)	36(29.7%)	37(30.6%)	**2.919** **(1.314–6.485)**
Deceased	25(20.7%)	23(19%)		12(10%)	36(29.7%)	
**Variable**	**Purchase independently before confinement**		**OR (CI)**	**Purchase independently after confinement**		**OR (CI)**
	**YES**	**NO**		**YES**	**NO**	
Barthel level:						
Severy dependence	4(3.2%)	33(26.8%)	0.574 (0.177–1.863)	2(1.6%)	34(28.1%)	0.441 (0.092–2.123)
Moderate dependence	15(12.2%)	71(57.8%)		10(8.3%)	75(62%)	
Income level:						
Less 11,200 euros/year	6(5%)	52(42.2%)	0.462 (0.163–1.307)	1(0.8%)	57(47.1%)	0.462 (0.163–1.307)
Greater 11,200 euros/year	13(10.6%)	52(42.2%)		11(9.1%)	52(43%)	
Survival June 2023:						
Alive	16(13%)	57(46.4%)	4.398 (1.208–16.010)	10(8.3%)	63(52.1%)	3.641 (0.763–17.461)
Deceased	3(2.4%)	47(38.2%)		2(1.6%)	46(38%)	
**Variable**	**Supervised purchase before confinement**		**OR (CI)**	**Supervised purchase after confinement**		**OR (CI)**
	**YES**	**NO**	**YES**	**NO**		
Barthel level:						
Severy dependence	5(4.1%)	31(25.4%)	0.995 (0.323–3.062)	3(2.5%)	33(27.3%)	0.612 (0.160–2.338)
Moderate dependence	12(9.8%)	74(60.7%)		11(9.1%)	74(61.1%)	
Income level:						
Less 11,200 euros/year	7(5.7%)	51(41.8%)	0.741 (0.262–2.095)	4(3.3%)	54(44.6%)	0.393 (0.116–1.330)
Greater 11,200 euros/year	10(8.2%)	54(44.3%)		10(8.3%)	53(43.8%)	
Survival June 2023:						
Alive	12(9.8%)	61(50%)	1.731 (0.569–5.269)	11(9.1%)	62(51.2%)	2.661 (0.702–10.093)
Deceased	5(4.1%)	44(36.1%)		3(2.5%)	45(37.2%)	

#### Analysis of the networks that determine the evolutionary courses of functional impairment according to the community dwelling and mobility.

Regarding the association between using mobility assistance devices and survival, among people who used at least one mobility aid when leaving their home (n = 68), 38.2% (n = 26) died in the period 2020–2023. This percentage was 47.1% (n = 16) among people who were homebound and used at least one mobility aid inside their home (OR 1.436, CI 0.625–3.301).

Going deeper into this analysis, the combination of both variables, community dwelling and use of mobility aids, showed differences in relation to survival (likelihood ratio 19.667; p = 0.003). Living in confinement implied a higher mortality (72.7%; n = 8) when these assistive devices were not used (n = 11) than when they were used (n = 34) (deaths 47.1%; n = 16). At the same time, when they were able to leave home using alternatively a mobility aid (n = 26), 46.2% (n = 12) died. When there was the ability to leave home using only one type of mobility aid, and this was a wheelchair (n = 8), 75% (n = 6) died. When, however, a crutches-cane (n = 25) or a walker (n = 9) was used to leave the house as the only mobility aid, the observed mortality was lower (28%, n = 7 and 11.1%, n = 1, respectively). Finally, among those who could leave home without using any mobility assistance device (n = 14), only 14.3% (n = 2) died during this period.

**Fig 3:**
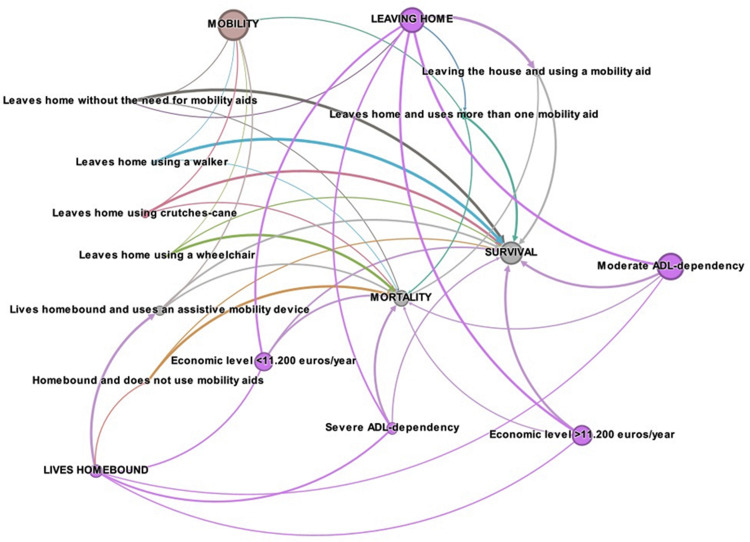
Network analysis for mobility. Network analysis of mobility, reflected by the interrelationships between the mode of living in the community, estimated by the ability to leave home or the situation of homebound living, and the use of assistive mobility devices according to the mode of living. Influence of mobility, level of dependency and economic level on survival at three years of follow-up. The values behind this network analysis (points, edges) are given in S1 Text.

These results suggest the existence of multiple pathways in functional dependence, and that being on one pathway or another carries a different mortality risk. These pathways have been represented in Fig 3, a figure in which, according to the mode of living in the community and the level of functional support required by means of a mobility aid, the differences in survival-mortality of each pathway can be appreciated, as well as the influence on these results of the level of dependence and economic level, and their combined effect on survival. The values behind this network analysis (points, edges) are collected in S1 Text. Each path in this network of functional dependency, each situation, is associated with a different probability of survival, which could be differentiated for approach from health planning.

Finally, having severe functional dependence and using a mobility aid (n = 29) was associated with a lower probability of survival (OR 0.242; CI 0.097–0.602). In this three-year period, 65.5% (n = 19) of the persons in this situation died. Among those with moderate functional dependence (n = 73), 31.5% (n = 23) died.

#### Social resources in relation to the level of dependence for ADL, housing situation, or the use of mobility assistance devices.

In relation to being functionally ADL-dependent, half (55.6%) of these dependent people had been assigned an assistant for housework for a few hours each week by the public services, a benefit that was partially subsidized. Being male or female did not influence the availability of this assistance (OR 1.000; CI 0.424–2.354). Assistance with personal care or housework in 36.3% of patients was supplemented or replaced by a private assistant. A full-time internal caregiver was present in 19.2% of the patients (Table 1).

**Fig 4:**
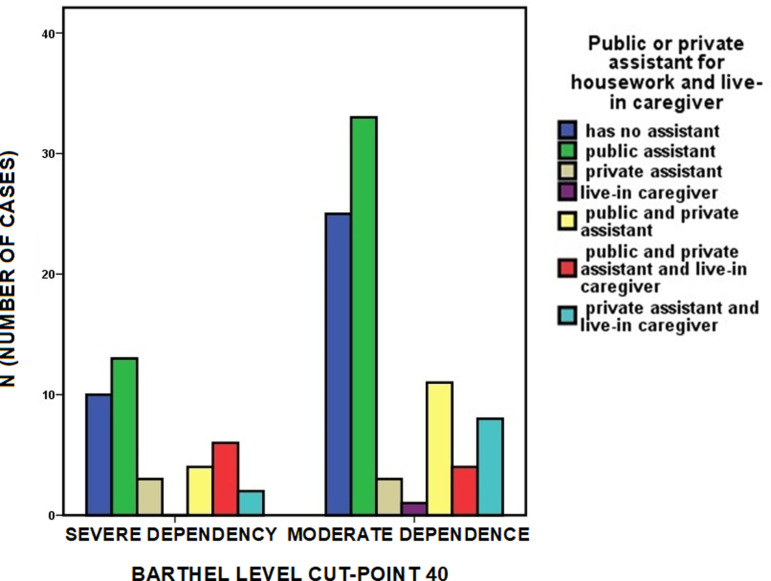
Level of functional dependence and availability of assistants. Availability of assistants for housework or live-in caregivers depending on the level of dependency. Coexistence of different types of assistants in relation to the level of dependency.

Analyzing this service with respect to the level of dependency, 42.1% (n = 16) of people with severe functional dependence (n = 38) and 45.5% (n = 40) of people with moderate dependence (n = 88) did not have a public assistant. Among people with severe functional dependence who had a public assistant, 23.7% (n = 9) also had a private assistant, and 15.8% (n = 6) had an internal caregiver (Fig 4). The data behind this figure, and all the figures in this manuscript, are given in S1 Text.

**Fig 5:**
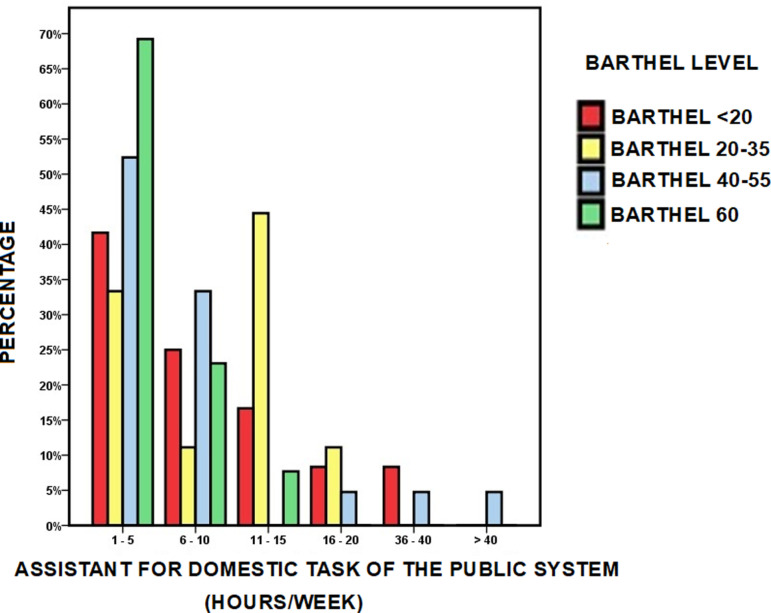
Level of functional ADL-dependency and weekly hours of public assistance for housework. Assistants assigned for housework by social services. Number of hours per week for each level of dependence on the Barthel index.

Most dependent people had an assistant assigned to them for household tasks and personal care of the dependent person for less than five hours a week (Fig 5). This situation was independent of the person’s level of ADL-dependency (z = -1.165; p = 0.244), their marital status (z = -0.521; p = 0.603) and their economic capacity (z = -0.675; p = 0.500).

In relation to the housing situation in the community, having a public assistant was more frequent when living independently (51.4%; n = 36) than when living with children (34.3%; n = 24) or with other people (14.3%; n = 10). However, having a private caregiver was more frequent when living with people other than children (44.4%; n = 20) than when living with children (35.6%; n = 16) or independently (20%; n = 9). This result was more evident with respect to having an internal caregiver, an assistant that 66.7% (n = 16) of dependent persons living with others, 29.2% (n = 7) of those living with their children, and 4.2% (n = 1) of those living independently had. Not having a public assistant for housework was more frequent when living with children (56%; n = 33) than when living independently (22%; n = 13) or with others (22%; n = 13).

Regarding the impact of the COVID-19 pandemic, during the confinement, 62.8% of ADL-dependent patients lost the public provision of home care, and 22.2% of them lost private attendants. All the internal caregivers maintained their activity. There was a change in caregivers during confinement for 18.9% of the public caregivers, 2.4% of the private caregivers and 0.8% of both types of caregivers.

On the other hand, having an assistant for housework assigned from public institutions was not associated with the use of crutches-cane (OR 1.067; CI 0.339–3.354), walker (OR 1.714; CI 0.431–6.826) or wheelchair (OR 0.804; CI 0.230–2.808). Among people who used crutches-cane or walker, having a public or private assistant to help with housework, or having an internal caregiver, was not associated with survival. Nor was it associated with living homebound (OR 1.300, CI 0.402–4.205) or having the ability to leave home (OR 1.789, CI 0.706–4.533).

In relation to these results, S2 Text, contains, in Table 1s, the complete data that reproduce these results. Also included in S4 Text, Fig 1s, is the analysis of the interrelationships between level of dependency, community dwelling, economic status, use of mobility aids, and availability of assistants for housework or an internal caregiver.

#### Community dwelling as a risk factor. Differences in survival as a function of morbidity and disease burden.

Community dwelling habits we assessed, as previously mentioned, by two indicators: having the ability to leave the house and carry out such activity on a regular basis; and living homebound, leaving the house only in obligatory situations.

Regarding health determinants, 26.8% of the people participating in the study had five or more chronic diseases; this situation was more frequent in men than in women (χ^2^ = 6.174; p = 0.046); this result was not associated with the level of economic income (χ^2^ = 0.617; p = 0.735) or the level of dependency (χ^2^ = 1.535; p = 0.464). Among chronic diseases, arterial hypertension was the most prevalent, followed by dyslipidemia, obesity, and diabetes (Table 7).

**Table 7:** Health data of the functional ADL-dependent population of the Orcasitas cohort. 1: Fisher’s exact statistic. 2: Chronic obstructive pulmonary disease.

Sociosanitary data of orcasitas cohort					
Chronic diseases. comparative analysis of living homebound – Leaving home					
Variable	Leaves home		Homebound		Odds ratio (CI)
	Alive	Deceased	Alive	Deceased	
Diabetes mellitus	25 (77.8%)	8 (24.2%)	8 (34.8%)	15 (65.2%)	**5.859** **(1.817–18.889)**
Hypertension	49 (69%)	22 (31%)	15 (36.6%)	26 (63.4%)	**3.860** **(1.716–8.682)**
Dyslipidemia	39 (69.6%)	17 (30.4%)	10 (43.5%)	13 (56.5%)	**2.982** **(1.094–8.123)**
Obesity	29 (67.4%)	14 (32.6%)	13 (61.9%)	8 (38.1%)	1.274 (0.429–3.781)
COPD^2^	8 (50%)	8 (50%)	0 (0%)	4 (100%)	-
Heart failure:	10 (52.6%)	9 (47.4%)	6 (42.9%)	8 (57.1%)	1.481 (0.369–5.946)
Asthma	6 (75%)	2 (25%)	0 (0%)	1 (100%)	-
Ischemic heart disease	15 (71.4%)	6 (28.6%)	5 (41.7%)	7 (58.3%)	3.500 (0.790–15.495)
Stroke	12 (75%)	4 (25%)	3 (30%)	7 (70%)	**7.000 (1.200–40.829)**
Kidney failure	11 (68.7%)	5 (31.3%)	2 (20%)	8 (80%)	**8.800 (1.348–57–428)**
**Chronic diseases and survival when living homebound or having the ability to leaves home**					
**Variable**	**Leaves home**		**Homebound**		**Statistical**
	**Alive**	**Deceased**	**Alive**	**Deceased**	
Diabetes mellitus:					
Yes	25 (46.3%)	8 (28.6%)	8 (38.1%)	15 (62.5%)	**χ**^**2**^ **= 6.174; p = 0.046**
No	29 (53.7%)	20 (71.4%)	13 (61.9%)	9 (37.5%)	χ^2^ = 2.670; p = 0.102
Hypertension					
Yes	49 (40.7%)	22 (78.6%)	15 (74.1%)	26 (95.8%)	χ^2^ = 2.351; p = 0.125
No	5 (9.3%)	6 (21.4%)	3 (28.6%)	1 (4.2%)	**χ**^**2**^ **= 5.078; p = 0.039**^**1**^
Dyslipidemia:					
Yes	39 (72.2%)	17 (60.7%)	10 (47.6%)	13 (54.2%)	χ^2^ = 1.128; p = 0.288
No	15 (27.8%)	11 (39.3%)	11 (52.4%)	11 (45.8%)	χ^2^ = 0.192; p = 0.661
Obesity					
Yes	29 (53.7%)	14 (50%)	13 (61.9%)	8 (33.3%)	χ^2^ = 0.101; p = 0.750
No	25 (46.3%)	14 (50%)	8 (38.1%)	16 (66.7%)	χ^2^ = 3.673; p = 0.055
COPD:					
Yes	8 (14.8%)	8 (28.6%)	0 (0%)	4 (16.7%)	χ^2^ = 2.222; p = 0.136
No	46 (85.2%)	20 (71.4%)	21 (100%)	20 (83.3%)	χ^2^ = 3.841; p = 0.111^1^
Heart failure:					
Yes	10 (18.5%)	9 (32.1%)	6 (28.6%)	8 (33.3%)	χ^2^ = 1.923; p = 0.166
No	44 (81.5%)	19 (67.9%)	15 (71.4%)	16 (66.7%)	χ^2^ = 0.118; p = 0.731
Asthma:					
Yes	6 (11.1%)	2 (7.1%)	0 (0%)	1 (4.2%)	χ^2^ = 0.330; p = 0.566
No	48 (88.9%)	26 (92.9%)	21 (100%)	23 (95.8%)	χ^2^ = 0.895; p = 1.000^1^
Ischemic heart disease:					
Yes	15 (27.8%)	6 (21.4%)	5 (23.8%)	7 (29.2%)	χ^2^ = 0.390; p = 0.532
No	39 (72.2%)	22 (78.6%)	16 (76.2%)	17 (70.8%)	χ^2^ = 0.164; p = 0.685
Stroke:					
Yes	12 (22.2%)	4 (14.3%)	3 (14.3%)	7 (29.2%)	χ^2^ = 0.740; p = 0.390
No	42 (77.8%)	24 (85.7%)	18 (85.7%)	17 (70.8%)	χ^2^ = 1.435; p = 0.231
Kidney failure:					
Yes	11 (20.4%)	5 (17.9%)	2 (9.5%)	8 (33.3%)	χ^2^ = 0.074; p = 0.785
No	43 (79.6%)	23 (82.1%)	19 (90.5%)	16 (66.7%)	χ^2^ = 3.673; p = 0.055

When the data were analyzed comparing living homebound versus having the ability to leave home, having diabetes and living homebound was associated with a lower probability of survival compared to those who could leave home (OR 5.859; CI 1.817–18.889). The same result was observed when having hypertension (OR 3.860; CI 1.716–8.682), dyslipidemia (OR 2.982; CI 1.094–8.123), renal failure (OR 8.800; CI 1.348–57.428), or a history of stroke (OR 7.000; CI 1.200–40.829), but not when having obesity, heart failure, or ischemic heart disease. 100% (n = 4) of people with chronic obstructive pulmonary disease living homebound died in the period 2020–2023 (Table 7).

**Fig 6:**
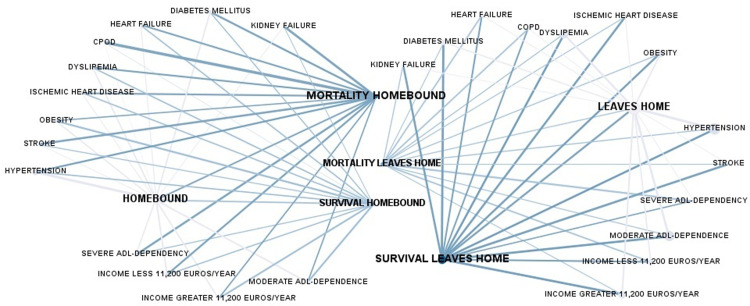
Survival-mortality network analysis for chronic diseases. Survival-mortality network analysis in relation to the chronic diseases present in the population with functional dependence of the Orcasitas cohort, according to living homebound or having the ability to leave home. Influence of the level of functional dependence and economic level on survival in both groups.

These results are also shown in Fig 6, where the thickness of the line, which represents the percentage of cases, shows the association with mortality or survival for each disease, both in the homebound group and in the group that can leave the house. At the same time, visualizing both groups together, leaving home - living homebound, the different shading of the lines in each group also represents the percentage of cases that make this course. This comparison allows us to observe that, within the group living homebound, there is a tendency to a worse evolution of chronic diseases, with mortality predominating, while in the group leaving home this tendency is reversed and survival predominates. The representation by means of a network system makes it possible to visualize the marked differences in survival between the two groups, living homebound and leaving home, highlighting the role of the leaving home factor with respect to survival and the role of the living homebound factor with respect to mortality, in relation to the same chronic diseases. The data behind Fig 6 are collected in S1 Text.

Parallel, among people living homebound a higher burden of chronic disease was associated with a lower probability of survival (OR 6.786; CI 1.280–35.966), a result that was not observed among people who maintained the ability to leave their home (Table 8). Polypharmacy did not influence survival, regardless of the mode of living in the community.

**Table 8:** Community living, socioeconomic and socio-health determinants and survival at three-year follow-up.

Community living, socio-health determinants and survival				
Variable	Leaves home (n = 82)		OR	CI
	Alive	Dead		
Barthel level:			**0.230**	**0.077–0.690**
Severe dependency	7 (8.6%)	11 (13.4%)		
Moderate dependence	47 (57.3%)	17 (20.7%)		
Income level:			**0.351**	**0.137–0.901**
Less 11,200 euros/year	19 (23.2%)	17 (20.7%)		
Greater 11,200 euros/year	35 (42.7%)	11 (13.4%)		
Educational level:			**4.545**	**1.014–19.845**
No education	50 (61%)	22 (27%)		
Has any level of education	3 (3.7%)	6 (7.3%)		
Age:			2.476	0.948–6.468
Over 90 years of age	14 (17.1%)	13 (15.8%)		
Under 90 years of age	40 (48.8%)	15 (18.3%)		
Sex:			**3.180**	**1.035–9.770**
Male	7 (8.6%)	9 (11%)		
Female	47 (57.3%)	19 (23.1%)		
Crutches-cane:			1.077	0.432–2.684
Yes	28 (34.1%)	14 (17.1%)		
No	26 (31.7%)	14 (17.1%)		
Walker:			0.839	0.327–2.152
Yes	19 (23.2%)	11 (13.4%)		
No	35 (42.7%)	17 (20.7%)		
Wheelchair:			**0.192**	**0.071–0.521**
Yes	11 (13.4%)	16 (19.5%)		
No	43 (52.5%)	12 (14.6%)		
Chronic disease burden:			0.648	0.221–1.898
More than 5 chronic diseases	16 (19.5%)	6 (7.3%)		
Less than 5 chronic diseases	38 (46.2%)	22 (27%)		
Polypharmacy (n = 81):			3.623	0,797–16.469
More than 5 drugs	50 (61.7%)	23 (28.4%)		
Less than 5 drugs	3 (3.7%)	5 (6.2%)		
**Variable**	**Lives homebound (n = 45)**		**OR**	**CI**
	**Alive**	**Dead**		
Barthel level:			**0.286**	**0.082–0–994**
Severe dependency	6 (13.3%)	14 (31.1%)		
Moderate dependence	15 (33.4%)	10 (22.2%)		
Income level:			0.450	0.136–1.488
Less 11,200 euros/year	9 (20%)	15 (33.4%)		
Greater 11,200 euros/year	12 (26.6%)	9 (20%)		
Educational level:			1.900	0.311–11.607
No education	19 (42.2%)	20 (44.4%)		
Has any level of education	2 (4.4%)	4 (9%)		
Age:			1.500	0.427–5.271
Over 90 years of age	6 (13.3%)	9 (20%)		
Under 90 years of age	15 (33.4%)	15 (33.4%)		
Sex:			3.000	0.677–13.285
Male	3 (6.7%)	8 (17.7%)		
Female	18 (40%)	16 (35.6%)		
Crutches-cane:			3.077	0.767–12.336
Yes	8 (17.8%)	4 (9%)		
No	13 (28.9%)	20 (44.4%)		
Walker:			2.250	0.635–7.973
Yes	9 (20%)	6 (13.3%)		
No	12 (26.7%)	18 (40%)		
Wheelchair:			1.214	0.343–4.298
Yes	7 (15.6%)	7 (15.6%)		
No	14 (31.1%)	17 (37.7%)		
Chronic disease burden:			**6.786**	**1.280–35.966**
More than 5 chronic diseases	2 (4.55)	10 (22.2%)		
Less than 5 chronic diseases	19 (42.2%)	14 (31.1%)		
Polypharmacy:			1.818	0.153–21.617
More than 5 drugs	20 (44.4%)	22 (49%)		
Less than 5 drugs	1 (2.2%)	2 (4.4%)		

However, when data were analyzed within each group, living homebound or leaving home, and not comparatively between the two groups, having one of the chronic diseases collected in the EAPEC was not associated with a lower probability of survival. Except in two situations, for hypertension among those living homebound and, unexpectedly, for the absence of diabetes mellitus among those who had the ability to leave home (Table 7).

In relation to disease burden and polypharmacy, persons living homebound did not have a higher disease burden or have a higher number of drugs prescribed than persons who maintained the ability to leave their home. However, having five or more chronic diseases and living homebound were associated with a lower probability of survival (OR 6.786; CI 1.280–35.966) (Table 8).

#### Ability to leave home versus being homebound as a criterion of vulnerability.

Living homebound was in this study a risk factor for mortality compared to having the ability to leave home, a risk that was independent of the disease burden and the number of drugs prescribed and was also associated with a worse outcome in most chronic diseases, in terms of mortality.

In this cohort of people with functional dependence, 64.6% (n = 82) left home regularly, and 35.4% (n = 45) lived homebound. About sex, 66% (n = 66) of the women and 59.3% (n = 16) of the men lived homebound (OR 1.335; CI 0.558–3.192). No differences in this situation were observed in relation to educational level (OR 1.231; CI 0.408–3.712). Neither was it associated with age, after distributing this cohort into two groups with a cut-off point of 90 years (OR 1.019; CI 0.471–2.205). Some 64.3% (n = 27) of those over 90 years of age and 64.7% (n = 55) of those under 90 years of age left their home on a routine basis.

Parallel, going out of the house before or after confinement was not influenced by the social situation of living with or not living with their children (OR 0.818; CI 0.395–1.697 and OR 0.670; CI 0.332–1.352, respectively). Nor with being married or not (OR 0.828; CI 0.379–1.808 and OR 0.961; CI 0.452–2.044, respectively); or being widowed or not (OR 1.358; CI 0.639–2.885 and OR 1.211; CI 0.585–2.507, respectively).

However, the ability to leave home was associated with the level of functional ADL-dependence (OR 0.352; CI 0.160–0.772), showing that 52.6% of people with severe dependence never left home, compared to 28.1% among people with moderate dependence (Table 6). A situation that was maintained after confinement (OR 0.353; IC 0.160–0.780).

On the other hand, confinement for the COVID-19 pandemic allowed us to observe how the level of economic income, which before confinement was not associated with the ability to leave home (OR 0.685; CI 0.330–1.421), influenced this ability after confinement. Having a lower economic capacity was associated after confinement with no longer leaving home (OR 0.347; CI 0.169–0.714) (Table 6).

With respect to other instrumental activities, the level of ADL-dependence was associated with the activity of leaving the house for non-compulsory activities, such as taking a walk, before confinement (OR 0.296; CI 0.132–0.666), but was not after confinement, a result that was associated with an increase in the percentage of people with moderate dependence who stopped performing this activity. (Table 6 and S2 Text, Table 2s).

This result showed another impact of confinement on these individuals. After the end of confinement, a loss of autonomy and independence, components of social capital, was observed. This loss was reflected in a significant decrease in the percentage of dependent people who went out of the house, which went from 64.57% to 51.97%, and affected both people who went out alone, decreasing their percentage from 12.6% to 7.9%, and those who went out accompanied by another person, whose percentage decreased from 53.5% to 44.9%. The percentage of persons who left the home for non-compulsory activities, such as leisure, also fell significantly, from 59.5% to 39.7%. Although not significantly, the percentage of people who made the necessary purchases for their daily sustenance autonomously or under supervision also decreased (Table 6 and S2 Text, Table 2s).

Also associated with survival was the ability to make the necessary purchases for food (OR 4.398; CI 1.208–16.010) before confinement for the COVID-19 pandemic. Among those who performed this activity (n = 19), 84.2% (n = 16) were still alive at 3 years of follow-up, compared to 54.8% (n = 57) among those who could not perform this activity (n = 104). Performing these purchases in a supervised manner (OR 1.731; CI 0.589–5.269) or maintaining both capacities after confinement (OR 3.641; CI 0.763–17.461 and OR 2.661; CI 0.702–10.093, respectively), was not associated with survival (S2 Text, Table 2s).

By contrast, living homebound increased the risk of mortality, with 53.3% (n = 24) of the dependent persons who were in this situation dying in the period 2020–2023, compared with 34.1% (n = 28) among those who were able to leave their home. When the activity of leaving home was necessarily accompanied by another person (n = 68), 41.2% (n = 28) died in this period. This percentage increased to 49.1% when analyzed in relation to people who continued to leave home accompanied after confinement (n = 57) (S2 Text, Table 2s).

In line with these results, a higher level of functional ADL-dependence was associated with a higher probability of mortality, both in people with functional dependence who had the ability to leave their home (OR 0.230; CI 0.077–0.690), and in those who lived homebound (OR 0.286; CI 0.082–0.994) (Table 8). Among those living homebound, 70% (n = 14) of those with severe dependence (n = 20) and 40% (n = 10) of those with moderate dependence died within the three-year follow-up period. Similarly, 61.1% (n = 12) of those who were able to leave their homes and were severely dependent (n = 18) died, and 26.6% (n = 17) of those who were moderately dependent (n = 64).

On other hand, among dependent persons who maintained the ability to leave home, a lower probability of survival was observed among males (OR 3.180; CI 1.035–9.770), a difference in relation to sex that was not observed among those who lived homebound (Table 8).

Furthermore, completing this analysis of community living as a vulnerability criterion, we analyzed the association between the use of mobility aids and survival, observing that, among those who left home independently, without the need to be accompanied by another person and without the need to use mobility aids (n = 14), 14.3% (n = 2) died in this three-year period. A second group of people with functional dependence included those who were accompanied by another person when they left home, but who did not need to use a mobility aid to leave home. In this group (n = 11), 18.2% (n = 2) died during the three years of follow-up.

Lastly, among those who left home accompanied by another person and required mobility aid to do so (n = 68), the results showed differences according to the device used. When a wheelchair was used to leave the house (n = 28), a lower probability of survival was observed (OR 0.245; CI 0.088–0.686), with 60.7% (n = 17) of its users dying, compared to 27.5% (n = 11) among those who left the house accompanied, but did not use a wheelchair (n = 40). Leaving home carrying a walker and accompanied by another person (OR 1.030; CI 0.384–2.766) or using crutches-cane (OR 1.154; CI 0.439–3.036) was not associated with survival. These interrelationships are represented graphically in Fig 3.

In parallel, persons who retained the ability to leave home after confinement by the COVID-19 pandemic (n = 66), but used a wheelchair for this purpose (n = 22), had a lower probability of survival than those not using this device (n = 44), with 45.5% (n = 10) dying, compared to 15.9% (n = 7) among those not using a wheelchair (OR 0.227; CI 0.071–0.728).

With respect to people living homebound, the use of mobility assistance devices was not associated with survival. Among the homebound, 31.1% (n = 14) used a wheelchair, 33.3% (n = 15) used a walker and 26.7% (n = 12) used crutches/cane to move around their homes. Among wheelchair users living homebound, 50% (n = 7) had a moderate level of dependence and 50% (n = 7) had a severe level of dependence.

Likewise, having a public or private assistant to aid in the performance of housework, or an internal caregiver, was not associated with increased survival among those who maintained the ability to leave the home (S2 Text, Table 1s), nor did it do so among those who lived confined to their home.

#### Autonomy and mobility. Role of assistive mobility devices.

##### 1. The ability to walk and the use of mobility aids as a nexus for the development of activities inside and outside the home

Within personal autonomy, the ability to walk has a specific relevance. This relevance is founded because it is the activity of the Barthel index that connects the ability to perform activities inside the home with the possibility of performing activities outside the home. Being able to walk enables the person to be able to go out on the outside independently, or with the assistance of another person or a mobility aid. Although in this study being independent or needing minimal assistance to walk was not associated with a greater likelihood of going out of the home (Table 3), greater independence in walking activity was associated with an increased likelihood of survival (OR 0.346; CI 0.142–0.842) (Table 4). Furthermore, after confinement, living homebound and not having the ability to walk was also associated with a lower probability of survival (OR 0.196; CI 0.049–0.778) (Table 4).

Regarding walking activity and the use of mobility assistance devices, 83.3% (n = 45) of the people who used crutches-cane (n = 54), 68.9% (n = 31) of the people who used a walker (n = 45) and 53.7% (n = 22) of those who used a wheelchair (n = 41) were people who reported needing assistance to be able to walk. Among people dependent on others for walking, the use of these devices was 5.6% (n = 3), 20% (n = 9) and 39% (n = 16), respectively.

Furthermore, and in relation to mobility, in this cohort, 12.1% (n = 14) of those who left home regularly and 24.4% (n = 11) of those who were homebound did not use any mobility aid. Mobility was assisted by an assistive device in the remaining 80.3% (n = 102), observing that when leaving home 24.5% (n = 25) exclusively used a crutch-cane, 8.8% (n = 9) a walker and 7.8% (n = 8) a wheelchair. Others (25.5%; n = 26) alternatively used one of these mobility aids when leaving home. Finally, 33.3% (n = 34) were persons with functional dependence who lived homebound and used a mobility aid in the home.

##### 2. The use of crutches-cane as a factor associated with functional capacity to perform ADLs and survival

Before confinement for the COVID-19 pandemic, 42.5% of the dependent persons in the Orcasitas cohort made daily use of crutches/cane to assist in walking, and 57.5% did not use them (Table 1). No differences were observed in the use of crutches-cane in relation to sex (OR 0.619; CI 0.263–1.455), level of income (OR 0.722; CI 0.356–1.466), or level of education (OR 1.143; CI 0.381–3.430), but it was more frequently used by people with moderate dependence (87%; n = 47) than severe dependence (13%; n = 7) (OR 0.202; CI 0.080–0.506).

Regarding the role that the use of crutches-cane could have on the ability to perform basic activities of daily living, when crutches-cane were used before confinement, a lower probability of dependence was observed in all the activities of the Barthel index, except in the activities of “bathing” and “anal sphincter control”. These same results were observed when their use was maintained after confinement, being associated, in addition, to a lower probability of presenting incontinence in the activity “anal sphincter control” when using crutches-cane (Table 9).

**Table 9:** Changes in the ability to perform basic activities of daily living included in the Barthel index during COVID-19 pandemic confinement in relation to the use of assistive mobility devices.

Mobility aids and Barthel index						
Barthel index activity	Uses crutches-cane before confinement		OR (CI)	Uses crutches-cane after confinement		OR (CI)
	YES	NO		YES	NO	
Walking on level surfaces						
Independent-minimal help	51(40.8%)	50(39.7%)	0.128	44(34.6%)	57(44.9%)	**0.308**
Does not walk-wheelchair	3(2.4%)	23(18.3%)	(0.036–0.453)	5(4%)	21(16.5%)	**(0.108–0.883)**
Dressing-undressing:						
Independent-minimal help	45(35.4%)	42(33.1%)	0.271	43(33.9%)	44(34.6%)	**0.181**
Dependent	9(7.1%)	31(24.4%)	(0.115–0.636)	6(4.7%)	34(26.8%)	**(0.069–0.474)**
Transfers bed-to-chair:						
Independent-minimal help	43(33.9%)	31(24.4%)	0.189	41(32.3%)	33(26%)	**0.143**
Dependent or great help	11(8.6%)	42(33.1%)	(0.084–0.424)	8(6.3%)	45(35.4%)	**(0.0.59–0.345)**
Up and down stairs:						
Independent-minimal help	26(20.5%)	22(17.3%)	0.465	25(19.7%)	23(18.1%)	**0.401**
Dependent	28(22%)	51(40.2%)	(0.224–0.965)	24(18.9%)	55(43.3%)	**(0.191–0.843)**
Urinary incontinence:						
Continent or occasional	42(33.1%)	41(32.3%)	0.366	40(31.5%)	43(33.9%)	**0.276**
Incontinent	12(9.4%)	32(25.2%)	(0.166–0.807)	9(7.1%)	35(27.5%)	**(0.118–0.647)**
Fecal incontinence:						
No	46(36.2%)	55(43.3%)	0.531	44(34.6%)	57(44.9%)	**0.308**
Yes	8(6.3%)	18(14.2%)	(0.212–1.334)	5(4%)	21(16.5%)	**(0.108–0.883)**
Toilet use:						
Independent	50(39.4%)	54(42.5%)	0.227	46(36.2%)	58(45.7%)	**0.189**
Dependent	4(3.1%)	19(15%)	(0.072–0.714)	3(2.4%)	20(15.7%)	**(0.053–0.676)**
Bathing:						
Independent	19(15%)	16(12.6%)	0.517	15(11.8%)	20(15.7%)	0.782
Dependent	35(27.5%)	57(44.9%)	(0.235–1.136)	34(26.8%)	58(45.7%)	(0.354–1.726)
Grooming:						
Independent	35(27.5%)	31(24.4%)	0.401	34(26.8%)	32(25.5%)	**0.307**
Dependent	19(15%)	42(33.1%)	(0.194–0.828)	15(11.8%)	46(36.2%)	**(0.144.0.654)**
Feeding:						
Independent	43(33.9%)	46(36.2%)	0.436	40(31.5%)	49(38.6%)	**0.380**
Dependent-minimal help	11(8.6%)	27(21.3%)	(0.193–0.985)	9(7.1%)	29(22.8%)	**(0.161–0.895)**
**BARTHEL INDEX ACTIVITY**	**Uses wheelchair before confinement**		**OR (CI)**	**Uses wheelchair after confinement**		**OR (CI)**
	**YES**	**NO**		**YES**	**NO**	
Walking on level surfaces						
Independent-minimal help	25(19.7%)	76(59.8%)	4.864	24(18.9%)	77(60.6%)	**5.133**
Does not walk-wheelchair	16(12.6%)	10(7.9%)	(1.958–12.086)	16(12.6%)	10(7.9%)	**(2.059–12.796)**
Dressing-undressing:						
Independent-minimal help	20(15.7%)	67(52.8%)	3.703	18(14.2%)	69(54.3%)	**4.685**
Dependent	21(16.5%)	19(15%)	(1.669–8.212)	22(17.3%)	18(14.2%)	**(2.083–10.538)**
Transfers bed-to-chair:						
Independent-minimal help	15(11.8%)	59(46.5%)	3.788	12(9.5%)	62(48.8%)	**5.787**
Dependent or great help	26(20.5%)	27(21.2%)	(1.733–8.278)	28(22%)	25(19.7%)	**(2.538–13.143)**
Up and down stairs:						
Independent-minimal help	7(5.5%)	41(32.3%)	4.425	6(4.7%)	42(33.1%)	**5.289**
Dependent	34(26.8%)	45(35.4%)	(1.769–11.071)	34(26.8%)	45(35.4%)	**(2.016–13.874)**
Urinary incontinence:						
Continent or occasional	22(17.3%)	61(48%)	2.107	20(15.7%)	63(49.6%)	**2.625**
Incontinent	19(15%)	25(19.7%)	(0.975–4.553)	20(15.7%)	24(18.9%)	**(1.206–5.715)**
Fecal incontinence:						
No	28(22%)	73(57.6%)	2.607	26(20.5%)	7559.1%)	**3.365**
Yes	13(10.2%)	13(10.2%)	(1.077–6.308)	14(11%)	12(9.4%)	**(1.381–8.201)**
Toilet use:						
Independent	29(22.8%)	75(59.1%)	2.821	27(21.3%)	77(60.6%)	**3.707**
Dependent	12(9.4%)	11(8.7%)	(1.120–7.105)	13(10.2%)	10(7.9%)	**(1.457–9.431)**
Bathing:						
Independent	8(6.3%)	27(21.3%)	1.888	7(5.5%)	28(22%)	**2.237**
Dependent	33(26%)	59(46.5%)	(0.770–4.627)	33(26%)	59(46.5%)	**(0.882–5.678)**
Grooming:						
Independent	14(11%)	52(40.9%)	2.950	12(9.4%)	54(42.6%)	**3.818**
Dependent	27(21.3%)	34(26.8%)	(1.356–6.414)	28(22%)	33(26%)	**(1.710–8.523)**
Feeding:						
Independent	26(20.5%)	63(49.6%)	1.580	23(18.1%)	66(52%)	**2.323**
Dependent-minimal help	15(11.8%)	23(18.1%)	(0.714–3.499)	17(13.4%)	21(16.5%)	**(1.048–5.151)**

Regarding the activities of the Barthel index that Granger associated with mobility, among those who used crutches-cane, 48.1% were independent for going up and down stairs, 79.6% for chair-to-bed transfers, 83.3% for dressing and undressing, and 94.4% for walking. Among those who did not use this device, these percentages were 30.1%, 42.5%, 48.3% and 68.5%, respectively.

In relation to survival, functionally dependent persons who did not use crutches-cane being dependent for any activity on the Barthel index was associated with a lower probability of survival. However, among functionally dependent persons who used crutches-cane, the level of dependence in performing basic activities of daily living included in the Barthel index was not associated with survival (Table 10).

**Table 10:** Use of assistive mobility devices, ability to perform basic activities of daily living included in the Barthel index and survival at 3-year follow-up. 1: Upper line: Odds Ratio in relation to survival among crutches-cane users. Lower line: Odds Ratio in relation to survival among non-crutches-cane users. 2: Upper line: Odds Ratio in relation to survival among wheelchair users. Lower line: Odds Ratio in relation to survival among non-wheelchair users.

MOBILITY AIDS, BARTHEL INDEX AND SURVIVAL						
**BARTHEL INDEX ACTIVITY**	**USE OF CRUTCHES-CANE**					
	**YES (n = 54)**		**NO (n = 73)**		**OR** ^**1**^	**CI**
	**ALIVE**	**DEAD**	**ALIVE**	**DEAD**		
Walking on level surfaces						
Independent-minimal help	33 (61.1%)	18 (33.3%)	32 (43.8%)	18 (24.7%)	0.917	0.831–1.012
Does not walk-wheelchair	3 (5.6%)	0 (0%)	7 (9.6%)	16 (21.9%)	**0.246**	**0.085–0.710**
Urinary incontinence:						
Continent or occasional	27 (50%)	15 (27.8%)	27 (37%)	14 (19.2%)	1.667	0.391–7.113
Incontinent	9 (16.6%)	3 (5.6%)	12 (16.4%)	20 (27.4%)	**0.311**	**0.119–0.816**
Fecal incontinence:						
No	30 (55.5%)	16 (29.6%)	34 (46.6%)	21 (28.8%)	1.600	0.289–8.859
Yes	6 (11.1%)	2 (3.7%)	5 (6.8%)	13 (17.8%)	**0.238**	**0.074–0.762**
Toilet use:						
Independent	34 (63%)	16 (29.6%)	34 (46.6%)	20 (27.4%)	0.471	0.061–3.648
Dependent	2 (3.7%)	2 (3.7%)	5 (6.8%)	14 (19.2%)	**0.210**	**0.066–0.671**
Up and down stairs:						
Independent-minimal help	20 (37.1%)	6 (11.1%)	17 (23.3%)	5 (6.8%)	0.400	0.123–1.302
Dependent	16 (29.6%)	12 (2.2%)	22 (30.1%)	29 (39.7%)	**0.223**	**0.071–0.698**
Bathing:						
Independent	15 (27.8%)	4 (7.4%)	13 (17.8%)	3 (4.1%)	0.400	0.110–1.459
Dependent	21 (38.9%)	14 (25.9%)	26 (35.6%)	31 (42.5%)	**0.194**	**0.050–0.754**
Dressing-undressing:						
Independent-minimal help	30 (55.5%)	15 (27.8%)	31 (42.5%)	11 (15%)	1.000	0.219–4.564
Dependent	6 (11.1%)	3 (5.6%)	8 (11%)	23 (31.5%)	**0.123**	**0.043–0.356**
Transfers bed-to-chair:						
Independent-minimal help	30 (55.5%)	13 (24.1%)	25 (34.2%)	6 (8.2%)	0.520	0.134–2.013
Dependent or great help	6 (11.1%)	5 (9.3%)	14 (19.2%)	28 (38.4%)	**0.120**	**0.040–0.360**
Grooming:						
Independent	15 (27.8%)	4 (7.4%)	13 (17.8%)	3 (4.1%)	0.400	0.110–1.459
Dependent	21 (38.9%)	14 (25.9%)	26 (35.6%)	31 (42.5%)	**0.194**	**0.050–0.754**
Feeding:						
Independent	29 (53.7%)	14 (25.9%)	30 (41.1%)	16 (21.9%)	0.845	0.212–3.372
Dependent-minimal help	7 (13%)	4 (7.4%)	9 (12.3%)	18 (24.7%)	**0.267**	**0.098–0.728**
**BARTHEL INDEX ACTIVITY**	**WHEELCHAIR USE**					
	**YES (n = 41)**		**NO (n = 86)**		**OR** ^**2**^	**CI**
	**ALIVE**	**DEAD**	**ALIVE**	**DEAD**		
Walking on level surfaces						
Independent-minimal help	13 (31.7%)	12 (29.3%)	52 (60.5%)	24 (27.9%)	0.420	0.112–1.565
Does not walk-wheelchair	5 (12.2%)	11 (26.8%)	5 (5.8%)	5 (5.8%)	0.462	0.122.1.746
Urinary incontinence:						
Continent or occasional	11 (26.8%)	11 (26.8%)	43 (50%)	18 (20.9%)	0.583	0.167–2.040
Incontinent	7 (17.1%)	12 (29.3%)	14 (16.3%)	11 (12.8%)	0.533	0.203–1.395
Fecal incontinence:						
Not, or occasional	14 (34.1%)	14 (34.1%)	50 (58.1%)	23 (26.7%)	0.444	0.111–1.787
Yes	4 (9.8%)	9 (22%)	7 (8.1%)	6 (7%)	0.537	0.162–1.777
Toilet use:						
Independent	16 (39%)	13 (31.7%)	52 (60.5%)	23 (26.7%)	**0.163**	**0.030–0.877**
Dependent	2 (4.9%)	10 (24.4%)	5 (5.8%)	6 (7%)	0.369	0.102–1.331
Up and down stairs:						
Independent-minimal help	4 (9.8%)	3 (7.3%)	33 (38.4%)	8 (9.3%)	0.525	0.101–2.721
Dependent	14 (34.1%)	20 (48.9%)	24 (27.9%)	21 (24.4%)	**0.277**	**0.105–0.730**
Bathing:						
Independent	6 (14.6%)	2 (4.9%)	22 (25.6%)	5 (5.8%)	0.190	0.033–1.097
Dependent	12 (29.3%)	21 (51.2%)	35 (40.7%)	24 (27.9%)	**0.331**	**0.110–0.997**
Dressing-undressing:						
Independent-minimal help	11 (26.8%)	9 (22%)	50 (58.1%)	17 (19.8%)	0.409	0.116–1.449
Dependent	7 (17.1%)	14 (34.1%)	7 (8.1%)	12 (14%)	**0.198**	**0.067–0.585**
Transfers bed-to-chair:						
Independent-minimal help	10 (24.4%)	5 (12.2%)	45 (52.3%)	14 (16.3%)	**0.222**	**0.057–0.865**
Dependent or great help	8 (19.5%)	18 (43.9%)	12 (14%)	15 (17.4%)	**0.249**	**0.095–0.655**
Grooming:						
Independent	7 (17.1%)	7 (17.1%)	39 (45.3%)	13 (15.1%)	0.688	0.188–2.520
Dependent	11 (26.8%)	16 (39%)	18 (20.9%)	16 (18.6%)	**0.375**	**0.149–0.942**
Feeding:						
Independent	14 (34.1%)	12 (29.3%)	45 (52.3%)	18 (20.9%)	0.312	0.078–1.239
Dependent-minimal help	4 (9.8%)	11 (26.8%)	12 (14%)	11 (12.8%)	0.436	0.163–1.167

Regarding the level of functional dependence, the use of crutches/cane was not associated with survival when the data were disaggregated into moderate (0.853; CI 0.344–2.116) and severe (OR 3.259; CI 0.604–17.591) level of dependence.

##### 3. Impact of COVID-19 pandemic confinement on the functional capacity of crutches-cane users to perform ADLs

After confinement for the COVID-19 pandemic 14.8% (n = 8) of those previously using crutches-cane stopped using them, while 4.1% (n = 3) started using this device.

Maintaining the use of crutches-cane as a mobility aid during the months of confinement in the usual home was associated with a greater probability of improving functional capacity (OR 3.621; 1.409–9.308). An 84.8% (n = 39) of the people who used crutches-cane improved their functional capacity during confinement, and 15.2% (n = 7) worsened their functional capacity. During confinement, it was also observed that 15.2% (n = 7) of people using crutches-cane and 39.4% (n = 26) of those who did not use them worsened their functional capacity.

Moreover, these results were maintained when the level of dependence was considered. In the group of people with moderate dependence, 82.1% (n = 32) improved their Barthel score and 17.9% (n = 7) worsened it; among those who did not use crutches-cane, 61% (n = 25) improved and 39% (n = 16) worsened their score (OR 2.926; 1.044–8.202). In the group with severe dependence, 100% (n = 7) of those who used crutches-cane improved their score during confinement, while among those who did not use this mobility device, 60% (n = 15) improved their score and 40% (n = 10) worsened it (OR 1.667; CI 1.210–2.295).

On the other hand, during confinement, maintaining the use of crutches-cane (n = 42) allowed 69% (n = 29) of its users to improve their Barthel index score and exceed the cut-off point of 60 established as a criterion for functional dependence, thus ceasing to be dependent. However, among those who did not use crutches-cane during confinement (n = 47), only 46.8% (n = 22) exceeded this cut-off (OR 0.394; CI 0.165–0.941). This difference was not observed in the group of people with severe functional dependence (OR 0.172; CI 0.020–1.522).

However, improving functional ability to the point of ceasing to be dependent was not associated with a higher probability of survival among people who used crutch-cane (OR 1.122; CI 0.353–3.567), although it was among people who during confinement did not use crutch-cane (OR 0.091; CI 0.024–0.343).

Parallel, and although survival at the end of the follow-up period was 66.7% (n = 36) among those using crutches/cane and 53.4% (n = 39) among those not using them, using crutches/cane from before confinement (OR 1.744; CI 0.841–3.615) or maintaining their use after confinement (OR 1.768; CI 0.839–3.723) was not associated with survival. Except in one circumstance: when using crutches-cane before confinement for the COVID-19 pandemic and still had the ability to leave home after confinement (n = 33). In this subgroup, 21.2% (n = 7) died in the three-year period following confinement, whereas 52.4% (n = 11) of those using crutches-cane and living homebound after confinement (n = 21) died (OR 4.086; CI 1.236–13.508).

##### 4. Impact of COVID-19 pandemic confinement on the ability of crutches-cane users to perform IADLs

In relation to mobility, the use of crutches-cane to assist in walking favored the ability of dependent persons to leave the home before confinement (OR 2.888; CI 1.311–6.362). Among those who used this support measure, 77.8% went out of the house before confinement, compared to 54.8% among those who did not use crutches-cane. Of the total number of dependent persons who left home before confinement, 51.2% used crutches-cane (Fig 3, Table 11).

**Table 11:** Mode of living in the community and its relationship to the use of mobility assistive devices. Effect of confinement due to the COVID-19 pandemic and influence on survival at 3-year follow-up. 1: OR: Odds ratio in relation to pre-confinement (upper line) and post-confinement (lower line) outcomes. 2: CI: pre-confinement (upper line) and post-confinement (lower line) confidence interval.

Changes during COVID-19 pandemic confinement in relation to mobility, community living, and use of mobility aids. effect on survivaL						
**Ability to leave home and mobility aids**						
**Variable**	**Before confinement**		**After confinement**		**OR** ^**1**^	**CI** ^**2**^
	**Leaves home**	**Homebound**	**Leaves home**	**Homebound**		
Wheelchair use:						
Yes	27 (32.9%)	14 (34.1%)	18 (27.3%)	23 (37.7%)	1.087	0.498–2.374
No	55 (67.1%)	31 (65.9%)	48 (72.7%)	38 (62.3%)	0.620	0.293–1.311
Crutches-cane use:						
Yes	42 (51.2%)	12 (22.7%)	33 (50%)	16 (26.2%)	**2.888**	**1.311–6.362**
No	40 (48.8%)	33 (73.3%)	33 (50%)	45 (73.8%)	**2.813**	**1.332–5.937**
Walker use:						
Yes	30 (36.6%)	15 (33.3%)	21 (21.8%)	17 (27.9%)	1.154	0.537–2.481
No	52 (63.4%)	30 (66.7%)	45 (68.2%)	44 (72.1%)	1.208	0.563–2.589
**Ability to leave home, mobility aids and survival**						
**Variable**	**Leaves home**				**OR** ^**1**^	**CI** ^**2**^
	**Before confinement**		**After confinement**			
	**Alive**	**Dead**	**Alive**	**Dead**		
Wheelchair use:						
Yes	11 (20.4%)	16 (42.8%)	12 (24.5%)	10 (58.8%)	**0.192**	**0.071–0.521**
No	43 (79.6%)	12 (57.2%)	37 (75.5%)	7 (41.2%)	**0.227**	**0.071–0.728**
Crutches-cane use:						
Yes	28 (51.8%)	14 (50%)	25 (51%)	8 (47%)	1.077	0.432–2.684
No	26 (48.2%)	14 (50%)	24 (49%)	9 (53%)	1.172	0.388–3.538
Walker use:						
Yes	19 (35.2%)	11 (13.4%)	17 (34.7%)	4 (23.5%)	0.839	0.327–2.152
No	35 (64.8%)	17 (20.7%)	32 (65.3%)	13 (76.5%)	1.727	0.487–6.121
**Variable**	**Leaves homebound**				**OR** ^**1**^	**CI** ^**2**^
	**Before confinement**		**After confinement**			
	**Alive**	**Dead**	**Alive**	**Dead**		
Wheelchair use:						
Yes	7 (33.3%)	7 (29.2%)	6 (23.1%)	12 (34.3%)	1.214	0.343–4.298
No	14 (66.7%)	17 (70.8%)	20 (76.9%)	23 (65.7%)	0.575	0.182–1.814
Crutches-cane use:						
Yes	8 (38.1%)	4 (16.7%)	8 (30.8%)	8 (22.9%)	3.077	0.767–12.336
No	13 (61.9%)	20 (83.3%)	18 (69.2%)	27 (77.1%)	1.500	0.476–4.724
Walker use:						
Yes	9 (42.8%)	6 (25%)	8 (30.8%)	9 (25.7%)	2.250	0.635–7.973
No	12 (57.1%)	18 (75%)	18 (69.2%)	26 (74.3%)	1.284	0.416–3.959
**Interrelationships use of mobility aids - ability to leave home**						
**Variable**	**Survival**				**OR** ^**1**^	**CI** ^**2**^
	**Before confinement**		**After confinement**			
	**Alive**	**Dead**	**Alive**	**Dead**		
Wheelchair use:						
Leaves home	11 (61.1%)	16 (69.6%)	12 (66.7%)	10 (45.5%)	0.688	0.188–2.520
Leaves homebound	7 (38.9%)	7 (30.4%)	6 (33.3%)	12 (54.5%)	3.592	0.980–13.164
No wheelchair use:						
Leaves home	43 (75.4%)	12 (41.4%)	37 (64.9%)	7 (23.3%)	**4.351**	**1.676–11.294**
Leaves homebound	14 (24.6%)	17 (58.6%)	20 (35.1%)	23 (76.7%)	**3.800**	**1.480–9.759**
Crutches-cane use:						
Leaves home	28 (77.8%)	14 (77.8%)	25 (75.8%)	8 (50%)	1.000	0.256–3.899
Leaves homebound	8 (22.2%)	4 (22.2%)	8 (24.2%)	8 (50%)	3.125	0.884–11.046
No Crutches-cane:						
Leaves home	26 (66.7%)	14 (41.2%)	24 (57.1%)	9 (25%)	**2.857**	**1.100–7.415**
Leaves homebound	13 (33.3%)	20 (58.8%)	18 (42.9%)	27 (75%)	**4.000**	**1.515–10.561**

This result was observed among those who left home independently, an activity performed by 47.6% (10), compared to 15.8% (6) among those who did not use this assistance device (OR 4.848; CI 1.428–16.458). As among those who went out accompanied by other people, an activity performed by 75% (n = 33), compared to 52.2% (n = 35) among those who did not use crutches-cane (OR 2,742; IC 1,191–6,315). In both cases, with respect to the dependent persons who never left the house, a group in which 25.6% (n = 11) were persons who used crutches-cane and 74.4% (n = 32) were persons who did not use crutches-cane.

However, using crutches-cane was not associated with leaving home for non-compulsory activities, such as taking a walk, either before or after confinement. Before confinement 67.9% (n = 36) of those using crutches-cane and 52.9% (n = 36) of those not using crutches-cane performed this activity (OR 1.882; CI 0.891–3.977). After confinement 46.9% (n = 23) of crutches-cane users and 34.7% (n = 25) of non-users continued to perform this activity (OR 1.663; CI 0.792–3.492).

After confinement, the percentage of dependent persons using crutches-cane and leaving home decreased to 61.1%, with the percentage of persons leaving home without using crutches-cane also decreasing (45.2%). The percentage of people living homebound increased, especially among people who, before confinement, did not use crutches-cane (OR 2.813; CI 1.332–5.937).

##### 5. Use of a walker as a mobility assistance device and interrelation with other variables

In relation to the use of a walker as a mobility assistance device, its use was not associated with any of the items of the Barthel index. This result was maintained when the data were analyzed with respect to the level of dependence, except for the item “anal sphincter continence”, which in patients with severe dependence showed that those who used a walker had more frequent anal sphincter control (OR 0.077; CI 0.09–0.684).

On the other hand, the use of a walker did not imply a greater probability of going out of the house (OR 1.154; CI 0.537–2.481), nor of being able to go shopping autonomously (OR 0.463; (0.143–1.497) or supervised by a family member (OR 2.455; CI 0.870–6.928).

Nor did their use before confinement imply differences in functional improvement during confinement (OR 1.233; CI 0.707–2.580). And maintaining its use after confinement did not imply a difference in functional capacity with respect to those who did not use this device (OR 1.073; CI 0.498–2.312).

Finally, neither was the use of a walker associated with survival (OR 1.227; CI 0.582–2.583). These results as shown in S3 Text.

##### 6. The use of wheelchairs as a factor associated with functional capacity and survival

###### 6.a. Wheelchair dependence and ability to perform ABVD. Implications for survival at three years.

Before confinement for the COVID-19 pandemic, 32.3% (n = 41) of the persons in this cohort used a wheelchair as a mobility assistance device. Of these, 56.1% (n = 23) were persons with moderate functional ADL-dependence and 43.9% (n = 18) were persons with severe functional ADL-dependence. The need to use a wheelchair was associated with a higher level of dependence (OR 2.583; CI 1.167–5.714), with 47.4% (n = 18) of people with severe dependence and 25.8% (n = 23) of people with moderate dependence requiring its use.

Dependence on a wheelchair for mobility was associated with greater dependence in performing basic activities of daily living (Table 9). With respect to people who did not use a wheelchair, this dependence increased after confinement, becoming visible, moreover, in activities that, previously to confinement, did not show this association, such as the activities of “feeding” and “bladder sphincter control”. Only the activity of “bathing” showed no association between being dependent for this activity and being a wheelchair user.

Furthermore, when these analyses were performed separately for each level of dependence, moderate and severe, differences were observed between both levels of functional dependence.

People with moderate functional dependence who used a wheelchair had more difficulties in performing chair-to-bed transfers, with 43.5% (n = 10) being independent or requiring minimal assistance for this activity, compared to 81.8% (n = 54) among people who did not use a wheelchair (OR 5.850; CI 2.078–16.466). This result was also observed with the activity of going up and down stairs, an activity for which 21.7% (n = 5) of wheelchair users and 56.1% (n = 37) of non-users were independent (OR 4.593; CI 1.523–13.849). Finally, 69.6% (n = 16) of those who used a wheelchair could independently or with minimal assistance perform the dressing-undressing activity, compared to 89.4% (n = 59) among those who did not use this mobility aid (OR 3.688; CI 1.128–12.053). No association was observed between a higher level of dependence in indicators such as being autonomous in using the toilet (OR 2.325; CI 0.479–11.282), urinary incontinence (OR 1.784; CI 0.608–5.229), the ability to walk autonomously (OR 2.568; CI 0.626–10.541), or being independent for feeding (OR 0.947; CI 0.272–3.295) and being a person with moderate functional dependence who uses a wheelchair.

However, among people with severe ADL-dependence, the use of a wheelchair was not associated with Barthel Index items reflecting mobility problems. Using or not using a wheelchair did not imply having greater dependence for performing chair-to-bed transfers (72.2%, n = 13 vs. 75%, n = 15, OR 0.867; CI 0.204–3.676), up-down stairs (88.9%, n = 16 vs. 80%, n = 16, OR 2.000; CI 0.320–12.510), or dressing-undressing (77.8%, n = 14 vs. 60%, n = 12, OR 2.333; CI 0.560–9.717). Nor was it associated with the rest of the Barthel index activities.

Over the cohort, and with respect to survival, using a wheelchair and being dependent in chair-to-bed transfer implied a lower probability of survival (OR 0.222; CI 0.057–0.865). In this population subgroup, 69.2% died in the three-year period 2020–2023, compared with 55.5% among persons who used a wheelchair but were independent for chair-to-bed transfer (Table 10). Also, those wheelchair users who presented a greater dependence on toilet use had a lower probability of survival (OR 0.163; CI 0.030–0.877).

Dependency to perform chair-to-bed transfers was also associated with higher mortality (OR 0.249, CI 0.095–0.655) among dependent non-wheelchair users (Table 10), with 55.5% (n = 15) of dependent persons (n = 27) dying in this period, compared to 20.3% (n = 12) among those who were independent or needed minimal assistance to perform this activity (n = 59).

Similar results were observed in other activities of the Barthel index associated with mobility in this population group that did not use a wheelchair, with 63.2% of the dependent persons dying in the activity “dressing-undressing” during this three-year period, compared to 12.3% among those who were independent for this activity (OR 0.198; CI 0.067–0.585). Also, in the activity of “going up and down stairs”, where 46.7% of the dependent persons for this activity died, compared to 19.5% among those who were independent (OR 0.277; CI 0.105–0.730). Finally, “walking independently” was not associated with survival (OR 0.462, CI 0.122–1.746), although higher mortality was observed among persons who “did not walk independently” and did not use a wheelchair (50%) than among persons who were independent for walking or were assisted by crutches or a walker (31.6%).

###### 6.b. Wheelchair dependency, ability to perform IADLs, community dwelling and survival.

Among the group of people leaving home, 32.9% did so in a wheelchair. The use of a wheelchair was not associated with an increased likelihood of leaving home, neither before confinement (OR 1.087, CI 0.498–2.374), nor after confinement (OR 1.194, CI 0.563–2.532). Before confinement, 34.1% of the dependent persons who used a wheelchair reported that they never left the house, except when necessary (medical consultation or administrative procedures requiring their presence) and 65.9% did leave the house. Among people with functional dependence who did not use a wheelchair, before confinement 36% lived homebound and 64% did leave their home (Table 11).

Since the COVID-19 pandemic confinement, the percentage of wheelchair-dependent persons living homebound increased from 31.1% to 56.1% (OR 2.464; CI 1.009–6.017). Among people who were living homebound but did not use a wheelchair, this increase after confinement was 36% to 44.2% (OR 1.404, CI 0.761–2.590).

Parallel, after confinement, 43.9% of wheelchair users and 55.8% of those who did not use a wheelchair continued to leave their home. Among wheelchair users who left home, 68.3% (n = 28) went out accompanied by another person. No person with functional dependence and the need to use a wheelchair left home independently.

Purchasing the products necessary for their sustenance autonomously, deciding which products needed to be purchased, was not possible for 94.9% (n = 37) of the dependent persons who used a wheelchair. Moreover, 89.7% (n = 35) could not carry out this activity under the supervision of a family member.

The mode of living in the community, homebound or able to leave home, was not associated either before (OR 0.688; CI 0.188–2.520) or after (OR 3.592; CI 0.980–13.164) confinement with the survival of persons using a wheelchair (Table 11). But this mode of living did influence the survival of those who did not use a wheelchair, both before (OR 4.351; CI 1.676–11.294) and after (OR 3.800; CI 1.480–9.759) confinement. About those who left home, 59.3% of those who left home in a wheelchair died in this three-year period, compared to 21.8% among those who left home but did not use a wheelchair (OR 5.212; CI 1.918–14.159).

###### 6.c. Assignment of social resources in relation to the functional capacity associated with needing a wheelchair.

Being a person who uses a wheelchair generally implies the need to be helped by other people. However, in this cohort, this situation was not associated with a higher probability of having an assistant assigned for housework and personal care from public institutions (OR 1.197; CI 0.563–2.543). Among those who used a wheelchair, 58.5% (n = 24) were assigned this assistant and 41.5% (n = 17) were not (S2 Text, Table 1s). Analyzing this result according to level of dependence, 77.8% (n = 14) of wheelchair users who had a public assistant had severe dependence (n = 18) and 43.5% (n = 10) had moderate dependence (n = 23) (OR 4.550; CI 1.141–18.151). Likewise, among those with a public assistant, 34.3% (n = 24) were wheelchair users and 65.7% (n = 46) were not.

Neither was being a wheelchair user associated with having hired a private assistant (OR 1.264; CI 0.582–2.748), a result that was maintained in relation to moderate (OR 1.257; CI 0.455–3.475) and severe (OR 0.978; CI 0.272–3.519) level of dependence. Among wheelchair users with severe dependence 44.4% (n = 8) had a private attendant and 55.6% (n = 10) did not. Among those with moderate dependence 36.4% (n = 8) had this assistant and 63.6% (n = 14) did not.

Regarding the need for a full-time caregiver at home, most wheelchair users (65.9%; n = 27) did not have an internal caregiver (S2 Text, Table 1s). However, 34.1% who had an internal caregiver represented 58.3% of the total number of people who had an internal caregiver in this cohort (n = 24). The remaining 41.7% (n = 10) were people who had an internal caregiver but did not use a wheelchair (OR 3.837; CI 1.524–9.661). This result was maintained among persons with severe functional dependence (OR 5.727; CI 1.004–32.673), but not among persons with moderate functional dependence (OR 3.063; CI 0.963–9.736). Among wheelchair users who had an internal caregiver, 46.7% (n = 7) of those with moderate dependence and 77.8% (n = 7) of those with severe dependence had an internal caregiver.

###### 6.d.- Influence of dependency level on mortality associated with dependence on a wheelchair for mobility. Changes during COVID-19 pandemic confinement.

**Fig 7:**
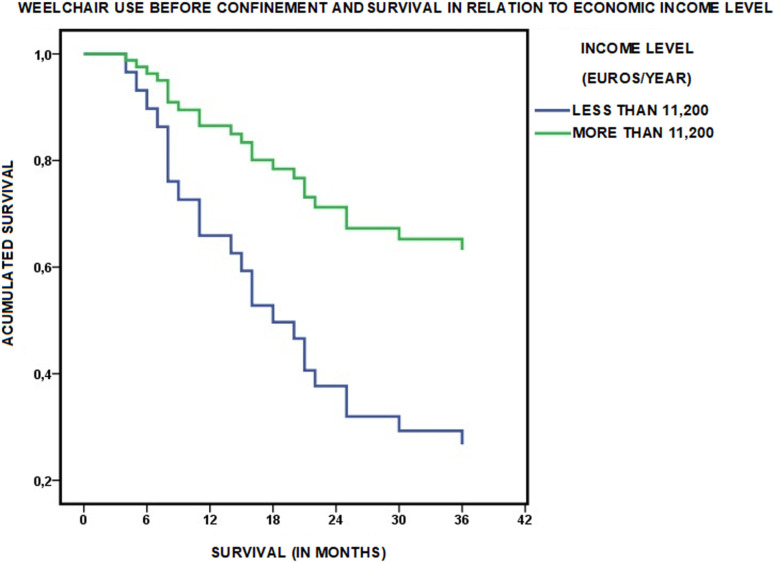
Cox regression: mortality risk associated with income level in wheelchair users. Association between level of economic income and survival at three years of follow-up in persons with functional ADL-dependence who were wheelchair users.

Using Cox regression, being dependent on a wheelchair for mobility was associated with increased mortality (HR 1.913; CI 1.106–3.309), noting that 56.1% (n = 23) of wheelchair users died in the period 2020–2023. In the group of people with functional dependence who did not use a wheelchair, 33.7% (n = 29) died in the same period. This mortality was mediated by the level of economic income, with a higher risk of mortality in those with a lower level of economic income (HR 2.376; CI 1.357–4.161) (Fig 7).

However, among wheelchair users, their level of dependence was not associated with survival (HR 1.946; CI 0.816–4.640). In the three-year period 2020–2023, out of the total number of deceased wheelchair users, 43.5% (n = 10) were moderately dependent and 56.5% (n = 13) were severely dependent, compared to 72.2% (n = 13) and 27.8 (n = 5) who were still alive (OR 0.296; CI 0.079–1.108). Out of the total number of deaths in the group with moderate functional dependence (n = 27), these deaths accounted for 37%.

In contrast, among dependent persons who did not use a wheelchair having severe dependence was associated with higher mortality at 3 years (OR 0.231; CI 0.081–0.662). Among those with severe dependence who did not use a wheelchair (n = 20), 60% (n = 12) died, while among those with moderate dependence (n = 66), 25.8% (n = 17) died.

Finally, 24.4% (n = 10) of the people who used wheelchairs before confinement improved their functional capacity during confinement until they were no longer functionally dependent, a percentage that was 52.3% (n = 45) among people who did not use wheelchairs. Using a wheelchair before confinement was a limiting factor for functional improvement during confinement (OR 3.402; CI 1.485–7.795).

This result was influenced by the level of functional ADL-dependence (OR 10.929; CI 1.231–97.038), observing that 94.4% (n = 17) of the people with severe dependence who used a wheelchair (n = 18) continued to be functionally ADL-dependent after confinement. However, among those with moderate functional dependence (n = 23), 39.1% (n = 9) improved their functional capacity and were no longer functionally ADL-dependent. In addition, 33.3% (n = 3) of them stopped using wheelchairs after confinement.

#### Economic level as a risk factor for mortality. Interrelationships between economic level, use of mobility aids, ability to leave home, and survival.

A higher level of dependency, lower economic capacity, dependence on a wheelchair for mobility, and living homebound were associated with a lower probability of survival. But these1 results required further analysis.

In relation to the level of dependency estimated by the ability to perform basic activities of daily living, among dependent persons with income above 11,200 euros/year, being “independent for dressing-undressing” (OR 0.237; CI 0.076–0.739) and being “independent for chair-to-bed transfers” (OR 0.283; CI 0.095–0.842) were associated with a higher probability of survival but being “independent for going up and down stairs” (OR 0.319; CI 0.100–1.0225) was not.

When the level of economic income was less than 11,200 euros/year being “dependent to go up-down stairs” (OR 0.266; CI 0.084–0.847), being “dependent to dressing-undressing” (OR 0.217; CI 0.066–0.714) and being “dependent to perform chair to -bed transfers” (OR 0.143; CI 0.045–0.456), was associated with a lower probability of survival at three years.

As a relevant factor, greater economic capacity was associated with a higher probability of survival (HR 2.47 (Exp(B) 0.405; CI 0.232–0.708). This result was modulated by the level of dependency. Thus, this result mainly affected people with severe dependence and economic income of less than 11,200 euros/year, a group in which 79.2% died, compared to 36.1% among those with this level of economic income but moderate dependence (OR 0.149; CI 0.045–0.492). However, when the economic capacity was greater than 11,200 euros/year, the level of dependence did not influence survival at three years (OR 0.479; CI 0.141–1.624).

**Fig 8:**
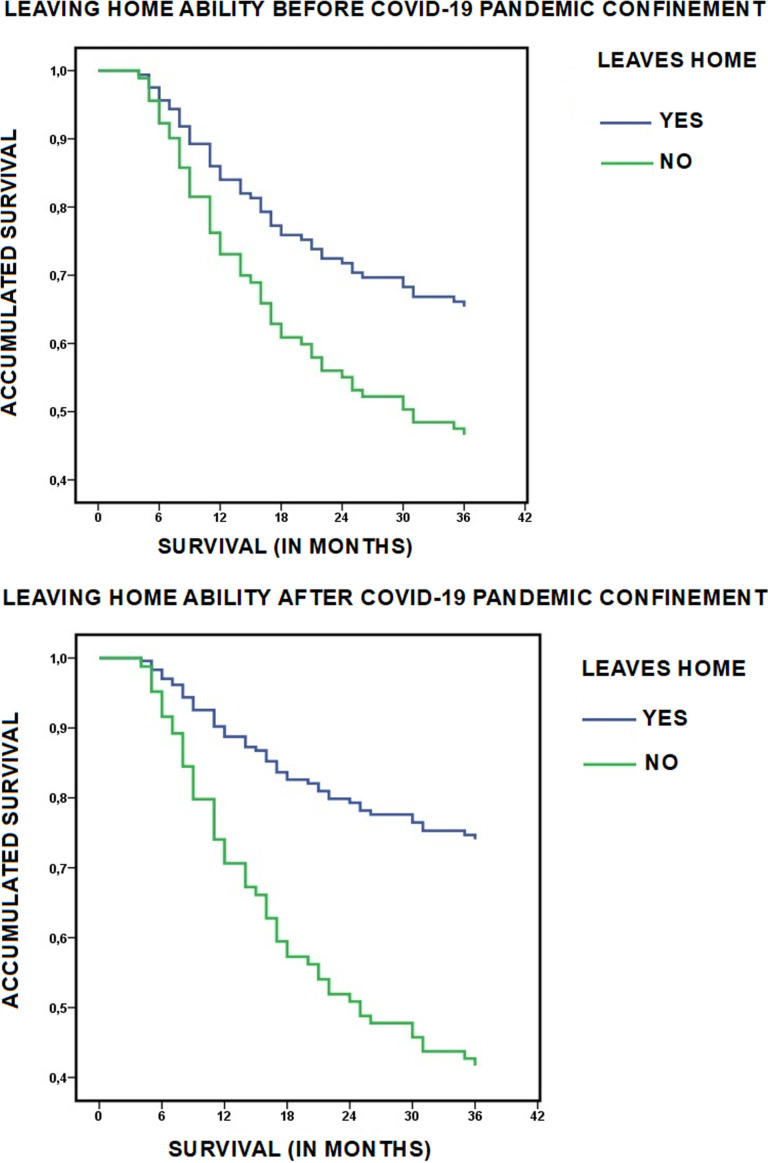
Panel A: Cox regression: mortality risk associated with mode of living in the community. Survival at three years follow-up in relation to maintaining before COVID-19 pandemic confinement the ability to leave home. Panel B: Cox regression: mortality risk associated with mode of living in the community. Survival at three years follow-up in relation to maintaining after COVID-19 pandemic confinement the ability to leave home.

On the other hand, using Cox regression, having the ability to leave home before (HR Exp(B) 0.556; CI 0.322–0.959) and after confinement (HR Exp(B) 0.343; CI 0.192–0.613) were associated with a lower risk of mortality (Fig 8, panels A and B). This result remained when the data were adjusted for level of dependency or level of income.

However, when both factors were combined, having a lower economic status was associated with a lower probability of survival among persons who before COVID-19 pandemic confinement had the ability to leave their home (OR 0.351; CI 0.137–0.901), but this result was not observed among persons living homebound (Table 6). Before the pandemic, 53.3% of people living homebound (n = 45) had an income of less than 11,200 euros/year and 46.7% had an income of more than 11,200 euros/year.

**Fig 9:**
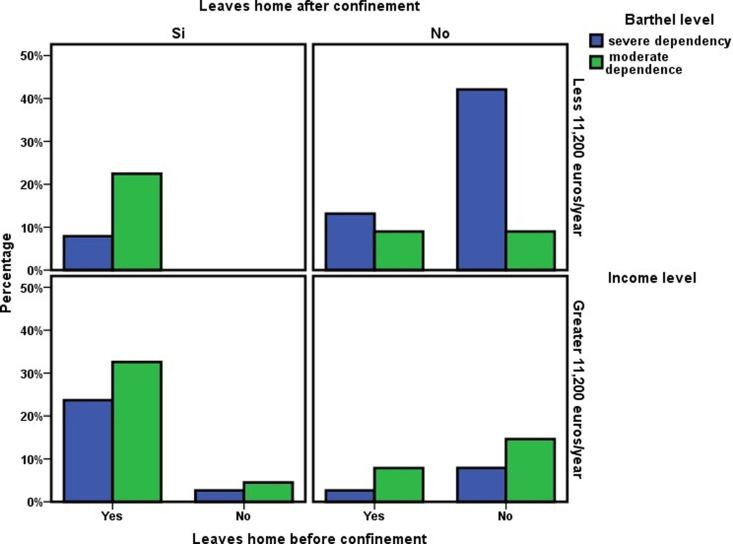
Influence of economic capacity on the mode of living in the community. Interrelationships between level of economic income and level of dependency with living in the community being able to leave home before and after confinement or living homebound.

Furthermore, during confinement, the baseline conditions of this cohort were modified, and it was observed that, after confinement, the number of homebound persons increased (n = 61). This increase occurred to a greater extent among people with an income of less than 11,200 euros/year, who accounted for 60.6% of those homebound, compared to 39.3% with a higher income, and having an income of less than 11,200 euros/year was associated with a greater probability of living homebound (OR 0.347; CI 0.169–0.714) (Fig 9). After confinement, 36.1% (n = 13) of people with a financial income of less than 11,200 euros/year who previously left home stopped doing so (OR 2.413; CI 1.159–5.023) (Table 6).

This change influenced the fact that, after confinement, living homebound and having a low income were associated with a lower probability of survival, with 84.6% (n = 11) of those in this situation dying, compared to 25% (n = 2) of those living homebound who had a higher income (OR 0.061; CI 0.007–0.546).

On the other hand, having a financial income of less than 11,200 euros/year and living homebound after confinement implied a lower probability of survival than those who continued to leave home (OR 6.697; CI 2.084–21.525). In the three-year period 2020–2023, 70.3% (n = 26) of people with this economic level who were living homebound (n = 37) and 26.1% (n = 6) of those who continued to leave home died. However, when income was above 11,200 euros/year these differences between leaving home or living homebound were not observed.

Likewise, among those who left home before confinement but stopped leaving home after confinement, 100% of those with severe dependence and 46.7% of those with moderate dependence died.

Finally, among people who after confinement remained functionally dependent and had a financial income of less than 11,200 euros/year (n = 37), 67.6% (n = 25) died, compared to 37.1% (n = 13) among people with an income of more than 11,200 euros/year (n = 35) (OR 0.284; CI 0.107–0.749). However, among those who improved their functional capacity during confinement and ceased to be dependent, this difference in survival according to economic capacity was not observed (OR 0.640; CI 0.189–2.170).

However, in the last group, made up of those who ceased to be functionally dependent after confinement, 38.2% (n = 21) were living homebound and 61.8% (n = 34) retained the ability to leave the home. Although 57.1% (n = 12) of those living homebound had a financial income of less than 11,200 euros/year and 67.6% (n = 23) of those leaving home had a higher income, the differences were not significant (OR 0.359; CI 0.117–1.104). Three years later, in this group that recovered their functional capacity during confinement, 38.1% (n = 8) of those living homebound and 17.6% (n = 6) among those able to leave home had died (OR 2.872; CI 0.826–9.986).

In contrast to these survival outcomes, remaining functionally dependent after confinement, living homebound and having a lower level of economic income were associated with a lower probability of survival (OR 0.127; CI 0.029–0.562). Among those with incomes below 11,200 euros/year (n = 25), 84% (n = 21) died, while among those with incomes above 11,200 euros/year (n = 15), 40% (n = 6) died. However, in contrast to the pre-pandemic situation, economic capacity did not influence survival among those who, after confinement, maintained the ability to leave home.

**Fig 10:**
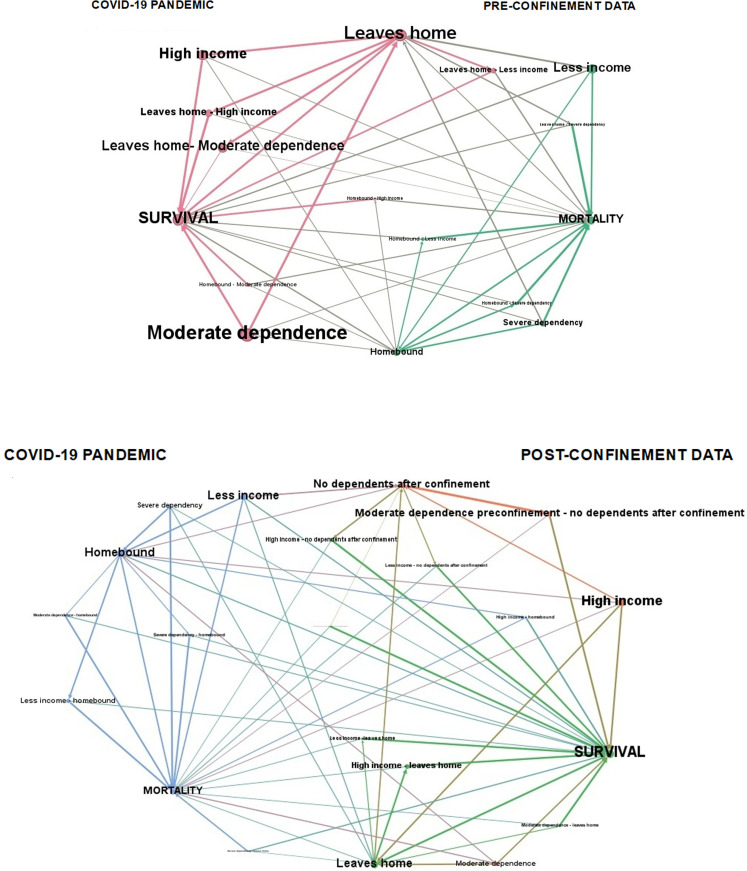
Panel A: Network analysis before COVID-19 confinement. Network analysis of the interrelationships between level of dependence, economic level, and mode of living in the community of people with functional dependence in the Orcasitas cohort. Analysis of the baseline situation before confinement due to the COVID-19 pandemic and influence on survival at three years. Panel B: Network analysis after COVID-19 confinement. Network analysis of the interrelationships between level of dependency, economic status, and mode of living in the community after COVID-19 pandemic confinement and their influence on survival at three years. Analysis of changes associated with COVID-19 pandemic confinement.

The picture of this cohort changed because of the COVID-19 pandemic, as can be seen in Table 5 and Fig 10, panels A and B. Panel A of Fig 10, with pre-pandemic data, shows the expected long-term evolutionary course if the pandemic had not occurred. However, the pandemic occurred, and this cohort changed, showing in panel B of Fig 10 the evolution of the same cohort, but with post-pandemic data. Between the two there is only a four-month difference. In this panel B a new cohort appears, that of people who, being dependent before the pandemic, ceased to be dependent after the pandemic because of the changes they made in their lives, and, as reflected in the graph and the values behind it collected in S1 Text, this change really changed their lives, clearly improving their probability of survival.

Confinement by the COVID-19 pandemic clearly had a beneficial effect on this cohort, in terms of survival. These changes, and their evolution in the three years of follow-up of this cohort, are shown in Fig 2s in S4 Text.

In the same line, within those who maintained after confinement the possibility of leaving the house for non-compulsory activities, such as walking, having a greater economic capacity was associated with greater survival at three years (OR 2.919; CI 1.314–6.485). In parallel, when after confinement there was no ability to leave the house for non-compulsory activities and the economic income was less than 11,200 euros/year, the probability of survival was lower (OR 6.697; CI 2.084–-21.525).

Finally, the factors analyzed only managed to explain a part of the percentage of variance in survival, with most of this variance remaining associated with other factors that were not analyzed in this study.

#### The Orcasitas cohort at three years of follow-up:

In June 2023, 25.4% (n = 14) of those who were no longer dependent after confinement and 67.9% (n = 38) of those who maintained functional dependence after confinement had died (OR 3.506; CI 1.556–7.897). Among those still alive, who represented 55.9% (n = 71) of the initial cohort, 43.3% (n = 55) were still residing at home, and 12.6% (n = 16) had entered a nursing home or changed residence. Four people left the study in June 2023. Change of residence was not associated with level of functional dependence, level of economic income or use of a wheelchair. Among those who completed the three-year follow-up period, 56.4% (n = 31) were persons who ceased to be dependent after confinement and 44.6% (n = 24) were persons who remained dependent after confinement. Regarding the mode of living in the community, 61.8% (n = 34) lived homebound and 38.2% (n = 21) maintained the ability to leave their home (Table 5).

Of the initial cohort of people with severe dependence (n = 38), only 18.4% (n = 7) were still alive after three years of follow-up. The rest of the people who in June 2023 presented severe dependence were people who presented functional impairment in these three years (n = 15). Within the group that ceased to be dependent after confinement, 56.4% completed the follow-up, 32.3% continued to maintain this situation, while 67.7% (n = 21) decreased their functional capacity, with 85.7% having moderate dependence and 14.3% severe dependence (Table 5). The effect of confinement on functional capacity in this cohort, its evolutionary course in the three years of follow-up and the results in terms of mortality reflected in Table 5 are shown in Fig 3s in S4 Text.

#### Trend analysis and post-hoc analysis.

In the trend analysis, having severe functional dependence, lower economic level, living homebound and using a wheelchair favored higher mortality. In contrast, having moderate functional dependence, a higher economic level, having the ability to leave the house, doing so without the need for mobility aids or using crutches-cane, favored survival (Fig 2s of S4 Text). The values behind this network analysis (points, edges) are given in S1 Text.

In post-hoc analyses using linear regression, having improved functional capacity during confinement, being a woman and having an economic capacity greater than 11,200 euros/year were associated with higher survival. Improving functional capacity explained 7.6% of the variance. This percentage increased to 17.6% when sex and economic capacity were included in the model (S2 Text, Table 3s). In another model, the level of dependency and sex explained 16.4% of this variance. Adding the ability to leave home and dependence for chair-to-bed transfer to the model explained 24.8% of the variance in survival (S2 Text, Table 4s).

## Discussion

In this study, confinement for the COVID-19 pandemic showed the role that various socio-health and socioeconomic factors play in the response to a crisis, as well affecting survival. It also showed that, in socioeconomically disadvantaged environments, microeconomics plays a relevant role in survival. People with poorer economic conditions, less mobility and higher levels of dependency responded worse to the stressor of the COVID-19 pandemic and showed higher mortality. They were also those who, to the most extent, saw their social relations limited, living predominantly homebound.

However, there is another reading as well. Unexpectedly, at least in terms of the level of response, these people, despite their advanced age, low educational level, low economic status, living in a socioeconomically disadvantaged neighborhood, being functionally dependent, and the social and family isolation of home confinement due to the COVID-19 pandemic, were able to respond to the pandemic.

Based on a very older population, with a mean age of 86 years, this study showed that there is no age limit for making changes [36]. Especially if the trigger for the need to change is a pandemic that puts our lives at risk [37]. Having the ability to change implied an advantage in terms of survival over those who were limited in this possibility.

Fragility, vulnerability and dependence rely on multiple factors [38] that end up creating a network that has its own modulating switches [39–41]. The first is personal idiosyncrasy [42], modulated by our genetics, lifestyles [43], the vicissitudes of life and comorbidities [44,45], factors that, by activating/deactivating heuristics, continually overwrite this idiosyncrasy, creating our personal history and conditioning the results.

However, the individual is not a whole, he is immersed in an environment, in a society, where rules of life and patterns of action are established and shared with other people, and end up forming a network, which often modulates his future perspectives [46,47]. This network feeds and feedback on its components, and creates its own heuristics, which activate/deactivate the changes that emerge in response to situations [48].

The confinement for several months in the usual home, decreed to deal with the COVID-19 pandemic, implied a change in the baseline conditions of this cohort, and triggered response mechanisms [49]. Factors such as the loss of the assistance provided by having an assistant at home to help with housework and personal care, the loss of the care provided in day centers, and having to assume functions that they had not previously performed [50], motivated these individuals to make changes.

On the other hand, the limitation of mobility to the home environment was expected to have an effect. With respect to functional capacity to perform activities within the home, this effect was assessed using the Barthel index. In response to confinement due to the COVID-19 pandemic, these individuals made changes that led to an improvement in their functional capacity at home, showing that dependence in non-institutionalized patients may have a functional reserve that can be addressed [51].

The effect of confinement was also evident in the changes observed in the ability to perform activities outside the home, activities that were severely restricted during the COVID-19 pandemic, leaving an increase in the number of functionally dependent persons living homebound as post-confinement sequel.

Twelve months after the onset of the COVID-19 pandemic, this cohort maintained a higher level of survival than that observed in persons with similar characteristics who were institutionalized [52–54]. A result that supports, in relation to health planning [55,56], considering confinement in a crisis [57], as a temporary measure, since living homebound is associated with worse health outcomes, with higher overall mortality and higher specific mortality for most chronic diseases, as was observed in this study.

In this study, confinement was the switch that motivated the changes [58] and mobility, the situation of living homebound or being able to leave home, the level of dependency and economic capacity were the main conditioning nodes of the results.

The concept of mobility includes many variables [59]. Having mobility implies being able to move around. However, moving around can be done in different ways. Furthermore, it can be performed for different purposes. Maintaining mobility within the home does not necessarily imply having the ability to leave the home.

In this cohort, most people required mobility assistance devices. Before confinement, almost half of the dependent persons used crutches-cane, and their use during confinement favored that these persons made changes that implied an improvement in their functional capacity, and even that, after confinement, half of them ceased to be functionally dependent. However, they continued to use crutches/cane, thereby indicating that their need was not only associated with functional dependence, but also with other factors.

Factors that were probably involved in the difference in terms of survival that were observed among people who during confinement improved their functionality until they were no longer dependent, and that mostly benefited those who did not use mobility aids. Indicating that the use of these devices made a difference with respect to those who did not use them.

Despite this, the use of crutches-cane favored independence at home, especially in those activities of the Barthel index that Granger [21] classified as associated with mobility. The use of crutches-cane was associated with a greater probability of being independent or requiring minimal assistance in activities such as “going up-down stairs”, “dressing-undressing”, “chair-to-bed transfer” and, above all, “walking independently”.

Making the activity of walking easy, the use of crutches-canes made it possible to go one step further, going outside. Both before and after confinement, using crutches-cane implied a greater likelihood of leaving home, and of doing so both alone and accompanied by another person. However, among those living homebound only one in four used crutches-cane.

Mobility was a main axis for survival, with a profile of a dependent person who had more autonomy because they had better mobility [60], and this greater autonomy implied a greater probability of survival.

This profile provided short- and long-term advantages when using crutches-cane, and probably a better quality of life [61,62]. During the months of confinement due to the COVID-19 pandemic, their use made it possible to improve functional capacity and to replace, at least partially, the help provided by other people in the environment to carry out daily activities [63,64], help that was limited or non-existent due to the pandemic. Making dependent people less dependent. However, this increased autonomy favored by using crutches-cane was only associated with an increased likelihood of survival in specific situations, such as maintaining the ability to leave home after confinement due to the pandemic.

These effects were less likely to occur with the use of walkers or wheelchairs, as reported in other studies [63]. Using a walker did not provide these advantages, nor did it influence survival.

In relation to the use of a wheelchair, when mobility was not available and the use of a wheelchair was required to move around, the functional dependence reflected by the Barthel index was greater, and this dependence increased after confinement. As foreseeable, this dependence was greater in those activities of the Barthel index associated with mobility. However, differences were observed according to the level of functional dependence, which were analyzed.

Among people with moderate functional dependence, using a wheelchair implied greater difficulty in performing “chair-to-bed transfers”, “up-down stairs”, “dressing-undressing” and “walking independently” than those who did not use this device. However, among people with severe dependence, the performance of these Barthel index activities showed no differences between wheelchair users and non-users. This result could be justified if the persons who did not use a wheelchair had a greater degree of functional deterioration and were bedridden, or had an “armchair-to-bed” lifestyle, situations that were not assessed in this study.

Regardless of their level of functional dependence, people who required a wheelchair for mobility had less autonomy and could not leave their homes unless accompanied by another person. Nor could they usually participate in the purchase of the products necessary for their sustenance. These limitations, associated with a lower probability of survival, both because they lived homebound or semi-homebound and because of the worse evolution of their chronic diseases in this situation, were not reflected in the level of dependency estimated by means of the Barthel index. Often, this level was that of “moderate dependency”, implying that health planning assigned them a level of care that was not in accordance with their real situation.

This situation is shown in the availability of assistants to perform housework, which was not associated with the level of dependency, nor did it consider the use of a wheelchair. Although people with a severe dependency in a wheelchair most often had a public assistant, people with moderate functional dependency in wheelchair most often had a private assistant or an internal caregiver. Being in a wheelchair and requiring an internal caregiver, or the help of a private caregiver, contrasted with having a moderate level of functional dependence.

Finally, the level of functional dependence influenced the capacity of this population to respond to the limitations imposed by confinement. This was more so when, in addition, a wheelchair is used. However, after confinement some people with moderate functional dependence in wheelchairs no longer met criteria for functional dependence, although they continued to use wheelchairs. This situation reflected that there was other wheelchair use needs, or other types of limitations, that should be addressed in other studies.

These new lines of research also respond to the need to differentiate those people who, despite using a wheelchair, having hired an internal caregiver and having a higher risk of mortality, are classified in the current model as moderate functional dependents.

Regardless of the cause of wheelchair use, half of the persons in this cohort who used a wheelchair at the beginning of this study had died within three years. Regarding the level of functional dependence, one out of every two people with severe dependence and one out of every three with moderate dependence who used a wheelchair died during this three-year period.

Thus, in this study, being a wheelchair user was a risk factor for mortality, and this risk was independent of another risk observed in this study, that associated with living homebound.

Functional dependency is an amalgam of situations in whose evolutionary course multiple factors are involved [64]. Factors that can influence the need to use mobility assistance devices or end up leading the dependent person to stay at home [65]. Several studies show that this final stage, being a homebound person, is associated with higher mortality, independently of functional deterioration and comorbidities [66–67]. In this and other studies, a higher level of dependency was associated with a greater likelihood of being homebound [68]. Living homebound also implies a higher mortality risk associated with most chronic diseases and disease burden. But there was also an increased risk of mortality when living homebound and not using mobility aids, a situation that probably responded to the presence of people with a worse baseline situation.

After confinement, the social isolation of this population increased, a situation that reflected the increase in the number of people living homebound, and this factor contributed to increasing their fragility, leading to worse health outcomes and a poorer quality of life [67,69]. Another finding was that one out of every three people who were no longer functionally dependent after confinement continued to live in homebound confinement, a situation that reflected the existence of other situations that conditioned this way of life [70].

In this study, the ability to leave home was a good indicator of the progression of functional impairment, of the effects of nationwide COVID-19 lockdown on such impairment and of the influence of economic level on these results [71].

Having the ability to leave the house before confinement and being able to do so in a non-compulsory way, to go for walks, were good indicators of a higher probability of survival at three years [72]. Probability increased when this capacity was preserved after confinement.

However, there were nuances. Getting out of the house was possible in multiple ways. The way it was done influenced outcomes.

When it was done autonomously, independently of other people and without the need for any mobility assistance device, survival was higher, with nine out of ten people in this situation still alive at the end of the study period.

In addition to being able to leave the house autonomously, without the need for mobility assistance devices, it was also possible to leave the house accompanied by another person, who provided functional support. In this situation, mortality was also low, like when the accompaniment of another person was not required.

However, on other occasions, in addition to this support person leaving the house, a mobility assistance device was required. More than half of the people with functional ADL-dependence who left their home accompanied by another person and required a wheelchair to leave the house died during the three years of follow-up. Almost half of the people who used a walker or crutches/cane also died. The functional limitation associated with the use of mobility aids has been associated in other studies with a higher rate of falls [73] and hospitalizations [74], and with increased mortality [75].

Using an assistive mobility device showed in this study a tendency for a worse prognosis in terms of survival. Except in two specific situations: when people who used crutch-cane prior to confinement for the COVID-19 pandemic maintained the ability to leave home after confinement and when people who used crutch-cane were independent or needed minimal assistance to perform most of the Barthel index activities that Granger associated with mobility.

Mobility was a relevant factor for survival [42]. Modulating, together with the level of dependency and economic capacity, this survival.

Having severe functional dependence implied a lower probability of survival regardless of whether one lived homebound or had the ability to leave home.

On the other hand, dependents with higher income were more likely to maintain the ability to leave the house after confinement, and to go out for walks. In contrast, confinement meant that a higher percentage of people with low economic resources were homebound, a situation that increased their risk of mortality [66].

A lower economic capacity, as an isolated factor, or associated with a higher level of functional dependence and/or living homebound, implied a higher mortality [66,76,77]. But the effect of economic level on survival was only observed among people with functional dependence, disappearing in those who were not functionally dependent.

As a final summary, in this study, carried out on a population with functional dependence for the performance of basic activities of daily living, the relevance of analyzing the data and evaluating the results from a holistic perspective, following a model focused on the pyramid defined by the King’s Fund [20], became evident. This model included the relationship patterns between variables and the networks established in these interrelationships.

The analysis of these networks made it possible to observe how the combination of certain variables conditioned the results, and how these combinations ultimately led to worse outcomes in the functionally dependent persons more vulnerable [66,68,76,78]. It also made it possible to detect vulnerable groups that classical analyses might not have detected, raising new research questions and recommendations for health planning. Finally, analysis using this network model suggests that long-term care may have gaps that can be a source of social inequalities [79], like those observed in nursing homes during the pandemic [80] or in population-based studies [67,81–83].

As limitations of this study, the context of the COVID-19 pandemic influenced the results, which likely would have been different had the pandemic not occurred [43]. But every crisis generates opportunities. And the confinement by the COVID-19 pandemic gave us the opportunity to observe the behavior of a vulnerable population in the face of a crisis. This behavior changed the evolutionary course that, predictably, and according to the mortality data collected in the “Plan for attention to frailty and promotion of healthy longevity in the older persons” of the Madrid Health Service [43], was to be expected in people with moderate functional dependence, mortality that, far from increasing due to the pandemic, unexpectedly decreased, a result that was associated with having the capacity to respond to the pandemic.

With respect to mobility assistance devices, the reference population for crutches-cane was people with moderate dependence. However, both devices present differences in design, and their users may present differential characteristics that were not analyzed, which is a limitation of this study. Neither was the type of cane used evaluated, a device for which there are dozens of varieties on the market, and there may be different types of cane users, which could have influenced the results described above [84].

Another limitation present, common in studies of the older populations, was that the life expectancy of women is longer than that of men. This situation leads to an over-representation of women in studies on the older population [85]. In this study, the sample size in some subgroups suggests that the results observed in these analyses should be considered in other population studies.

Moreover, the evaluation of people living confined to their homes did not include the existence of people who are bedridden, or people who only live in chair-to-bed mode, with no mobility beyond this displacement. Thus, the results observed in this population group may be subject to biases in relation to the variables analyzed in this study.

In the same line, wheelchair use could include situations that are not associated with disease or severe functional impairment, such as the presence of an older partner as the primary caregiver. These situations could have influenced the results.

Finally, the factors analyzed only managed to explain a part of the percentage of variance in survival, with most of this variance remaining associated with other factors that were not analyzed in this study.

## Conclusions

In the face of current ageism, this study showed that being older is not incompatible with making life-enhancing changes. Neither had functional dependence, showing that, even in adverse conditions, people still have some margin for response.

However, having functional dependence implies an elevated risk of mortality in this socioeconomically disadvantaged population. This recognized risk factor is modulated by multiple factors, with economic capacity and educational level playing a relevant role.

The Orcasitas cohort provides a novel model, where the educational level factor is practically homogeneous. This situation has allowed us to observe the effect of other factors without interference associated with the cultural level. As a result, and despite also having a low economic income as a cohort, it was observed that this economic income is capable of modulating both dependence and survival. This shows that microeconomics plays a relevant role in health status and survival of ADL-dependent persons residing in socioeconomically disadvantaged areas.

The progression towards dependency implies a loss of social capital, which increases the vulnerability of non-institutionalized people with functional dependency. On this path, mobility, the loss of mobility, becomes one of the central axes in the evolution towards dependency. Having mobility implies being more autonomous, being able to leave the house and maintain social and personal capital. And its maintenance becomes an objective associated with better health and survival outcomes.

One of the main results of this study was to prove that people with functional dependence can follow many evolutionary pathways, and that each pathway had a different probability of mortality, a fact that is clearly useful in health planning. It also made it possible to evaluate the different variables that favored some people to increase their functional capacity more than others in response to confinement due to the COVID-19 pandemic, creating new targets for action on which to work to increase functional capacity.

The classic assessment using the Barthel index provides relevant information, but it is incomplete for an analysis that would allow the design of strategies for healthy aging. Health and social planning would benefit from the information provided by other indicators, and from the creation of algorithms based on the patterns and networks established within each community.

The results of this study allow us to infer that in moderate functional dependence there are two population groups, depending on whether they need to use a wheelchair, groups that should be approached in a differentiated manner from the point of view of health planning. The mortality observed among wheelchair users requires a specific approach for this group, recommending an individual evaluation and a differentiated follow-up.

On the other hand, these findings suggest the existence of an intermediate step in the evolution of functional ADL-dependence, a step that the Barthel index cannot differentiate and that would be located within moderate dependence. There would be moderate dependence without mobility problems, or with slight mobility problems that would require the use of crutches/cane. And a moderate dependency with significant mobility problems, which would require the use of a wheelchair. Both situations have a different behavior in relation to survival, with the use of a wheelchair being associated with greater mortality. Therefore, the baseline situation leading to the use of a wheelchair should be differentiated in the assessment and assignment of risk in people over 70 years of age, or over 65 years of age with objectifiable functional limitations in the consultation.

They also allow us to suggest that the use of crutches/cane is probably associated with a greater well-being of the person, by allowing her to be more autonomous in the performance of basic and instrumental activities of daily living, and to limit dependence on other people. Facilitating, among other activities, that the dependent person can leave the house.

Leaving the house was an activity that not only potentially favored the maintenance of the individual’s social role, but also had an impact on survival. This impact was amplified among those who maintained the ability to leave home after the COVID-19 pandemic. In relation to vulnerable groups, this result may have implications for the management of future pandemics. In contrast, living homebound presented a higher risk of overall mortality and mortality associated with chronic diseases.

The mixed assessment, using autonomy indicators and functional indicators, provided a more complete overview of dependent persons. And, as this study shows, it was more sensitive to the impact on individuals of intercurrent processes affecting them, such as the COVID-19 pandemic.

In relation to the COVID-19 pandemic, the mortality observed among people with functional dependence in the period May 2020 to December 2020, a period associated with COVID-19 waves associated with higher mortality among the older population, allows us to suggest that the confinement of high-risk groups during pandemics has a positive effect on survival.

In the management of these future pandemics, vulnerable populations should be monitored in a differentiated manner, and strategies for the maintenance of functional capacities should be established in health planning. Personalized medicine should include facets beyond pharmacological treatments.

The study carried out contributes to providing “targets” on which health planning should have an impact. It also makes it possible to identify factors whose evaluation can provide greater efficiency to the actions designed from the point of view of health planning. The joint use of these indicators would make it possible to establish more personalized “roadmaps”, which would increase the quality of the health and social care provided to people with functional dependency who are not institutionalized.

## Supporting information

S1 Text Data behind the figures. (PDF)

S2 Text Tables associated with the study. (PDF)

S3 Text Basic data of the study. (PDF)

S4 Text Other Figures. (PDF)

## Abbreviations

ADL: basic activities of daily living
IADL: instrumental activities of daily living
OR: Odds ratio
HR: Hazard ratio
CI: confidence interval
EAPEC: Strategy of Care for People with Chronic Diseases
IHIC: Individual Health Insurance Card
COPD: chronic obstructive pulmonary disease
