# Supplementary material for: Socio-health factors, ability to perform instrumental and basic activities of daily living, and use of assistive mobility devices during the COVID-19 pandemic: Interrelationships and impact on long-term survival
Source: PLoS One. 2025 May 19;20(5):e0318481. doi: 10.1371/journal.pone.0318481 (PMC12088529; doi:10.1371/journal.pone.0318481)
Supplement: S1 Text — (PDF) [file pone.0318481.s001.pdf]

Supplement 1, “Data behind the figures”

Socio-health factors, ability to perform instrumental and basic activities of daily living, and use of assistive mobility devices during the Covid-19 pandemic. Interrelationships and impact on long-term survival.

Authors:

Vicente Martín Moreno. María Inmaculada Martínez Sanz. Irene Sánchez González. Miguel Recuero Vázquez. Sara Guerra Maroto. Miriam Fernández Gallardo. Amanda Martín Fernández. Julia Herranz Hernando. María Palma Benítez Calderón. Eva Sevillano Fuentes. Elena Pérez Rico. Laura Calderón Jiménez. Elena Sánchez Rodríguez. Helena Alonso Samperiz. Irene León Saiz. On behalf of GIDO collaborative group (Orcasitas Dependency Research Group). Juana Marcos Guerra.

Address for correspondence:

Vicente Martín Moreno

e-mail: amanvic@hotmail.com

Data behind the figures:

Figures made with the Gephi Graph® 0.10.1 program consist of nodes and lines. Both nodes and lines have been made maintaining proportionality with respect to their weight within the graph. The size of the text describing the node shows the relevance of that node with respect to the whole data.

Figure 2: Influence of Covid-19 confinement on the ability to leave home and survival.

Survival at three years of follow-up in relation to the changes produced during Covid-19 confinement in the mode of living in the community of persons with functional dependence in the Orcasitas cohort.

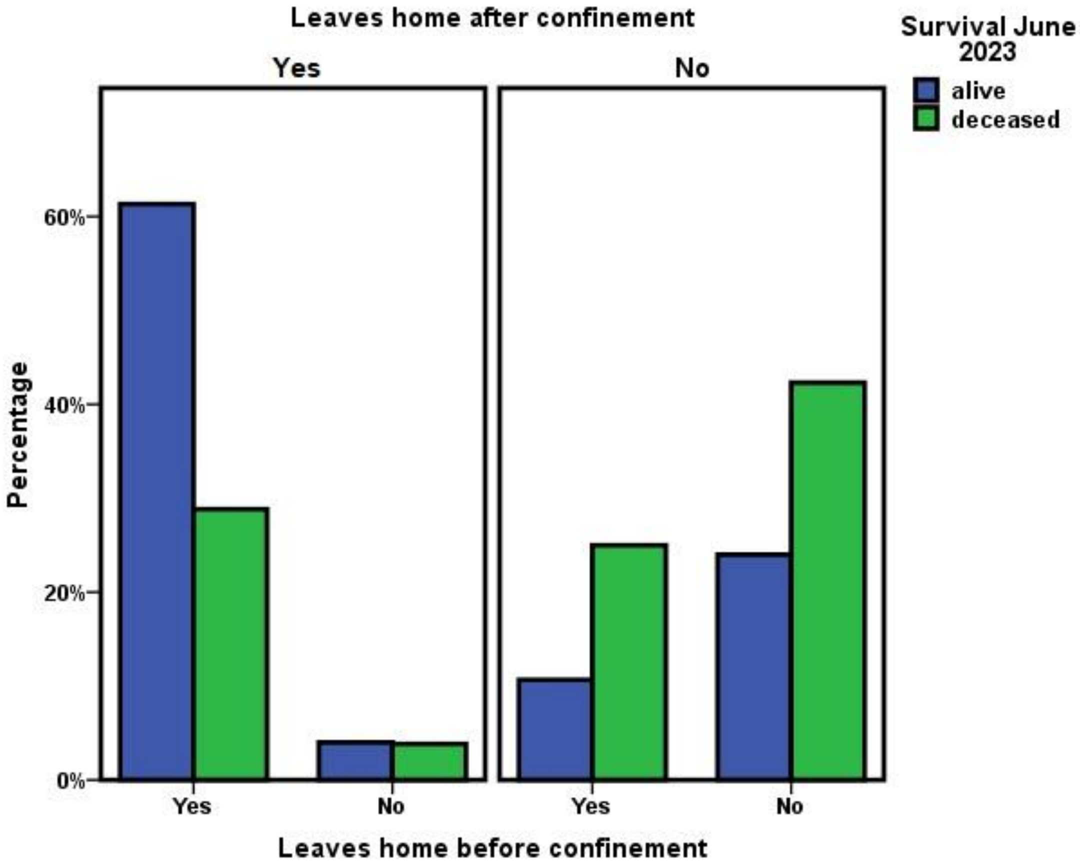

SPSS does not provide the results in tabular form to produce these graphs. We have provided the partial analyses as information, which also allow the graph to be interpreted correctly:

Contingency table: Leaves home before confinement

			Leaves home before confinement		
			Yes	No	Total
survival in June 2023	alive	Count	54	21	75
		Expected frequency	48.4	26.6	75.0
		% within survival in June 2023	72.0%	28.0%	100.0%
		% within Leaves home before confinement	65.9%	46.7%	59.1%
	deceased	Count	28	24	52
		Expected frequency	33.6	18.4	52.0
		% within survival in June 2023	53.8%	46.2%	100.0%
		% within Leaves home before confinement	34.1%	53.3%	40.9%
Total		Count	82	45	127
		Expected frequency	82.0	45.0	127.0
		% within survival in June 2023	64.6%	35.4%	100.0%
		% within Leaves home before confinement	100.0%	100.0%	100.0%

Chi-square tests

	Value	gl	Asymptotic sig. (bilateral)	Exact sig. (bilateral)	Exact sig. (unilateral)
Pearson's Chi-square	4.424[a]	1	0.035		
Continuity correction[b]	3.666	1	0.056		
Likelihood ratio	4.400	1	0.036		
Fisher's exact statistic				0.040	0.028
Linear by linear association	4.389	1	0.036		
N of valid cases	127				

a. 0 cells (.0%) have an expected frequency of less than 5. The minimum expected frequency is 18.43.
b. Calculated only for a 2x2 table.

Risk estimation

	Value	95% confidence interval	
		Lower	Upper
Survival Odds ratio in June 2023 (alive/deceased)	2.204	1.049	4.631
For the cohort ExitHomebeforeconfinement = Yes	1.337	1.002	1.784
For the cohort ExitHomebeforeconfinement= No	0.607	0.380	0.968
N de casos válidos	127		

Contingency table: Leaves home after confinement

			Leaves home after confinement		Total
			Yes	No	
survival in June 2023	alive	Count	49	26	75
		Expected frequency	39.0	36.0	75.0
		% within survival in June 2023	65.3%	34.7%	100.0%
		% within Leaves home after confinement	74.2%	42.6%	59.1%
	deceased	Count	17	35	52
		Expected frequency	27.0	25.0	52.0
		% within survival in June 2023	32.7%	67.3%	100.0%
		% within Leaves home after confinement	25.8%	57.4%	40.9%
Total		Count	66	61	127
		Expected frequency	66.0	61.0	127.0
		% within survival in June 2023	52.0%	48.0%	100.0%
		% within Leaves home after confinement	100.0%	100.0%	100.0%

Chi-square tests

	Value	gl	Asymptotic sig. (bilateral)	Exact sig. (bilateral)	Exact sig. (unilateral)
Pearson's Chi-square	13.108[a]	1	0.000		
Continuity correction[b]	11.833	1	0.001		
Likelihood ratio	13.333	1	0.000		
Fisher's exact statistic				0.000	0.000
Linear by linear association	13.004	1	0.000		
N of valid cases	127				

a. 0 cells (.0%) have an expected frequency of less than 5. The minimum expected frequency is 24.98.

b. Calculated only for a 2x2 table.

Risk estimation

	Value	95% confidence interval	
		Lower	Upper
Survival Odds ratio in June 2023 (alive/deceased)	3.880	1.834	8.211
For the cohort ExitHomeafterconfinement = Yes	1.998	1.309	3.052
For the cohort ExitHomeafterconfinement = No	0.515	0.358	0.741
N of valid cases	127		

Figure 3: Network analysis for mobility.

Network analysis of mobility, reflected by the interrelationships between the mode of living in the community, estimated by the ability to leave home or the situation of homebound living, and the use of assistive mobility devices according to the mode of living. Influence of mobility, level of dependency and economic level on survival at three years of follow-up.

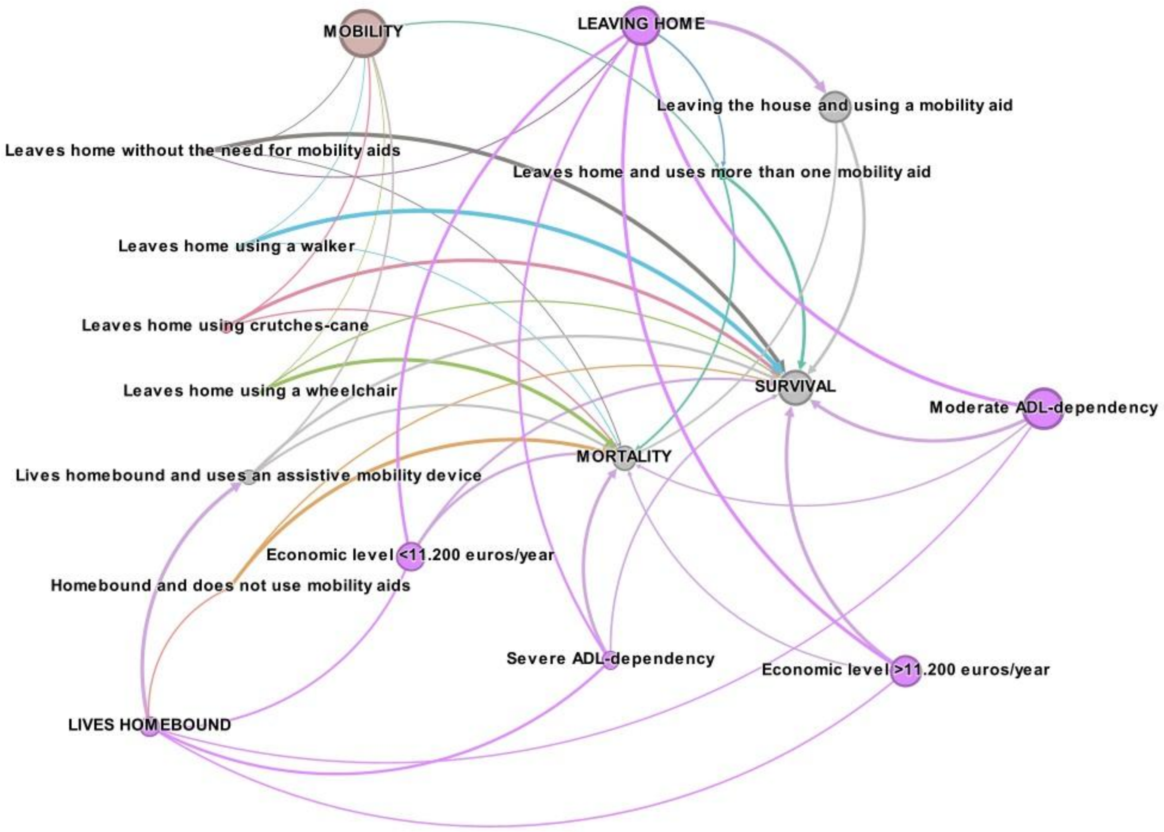

Data behind the figure 2:

Source. Id. Label. Weight
**Nodes n (%)**
1.- Economic income greater than 11.200 euros/year. n= 67 (52.8%)<br>2.- Economic income less than 11.200 euros/year. n=60 (47.2%)<br>3.- Moderate ADL-dependence. n=89 (70.1%)<br>4.- Severe ADL-dependency. n=38 (29.9%)<br>5.- Mobility (9+10+11+12+15). n=102 (80.3%)<br>6.- Leaving home (7+8). n=82 (64.6%)<br>7.- Leaves home without the need for mobility aids. n=14 (11%)<br>8.- Leaving the house and using a mobility aid. n=68 (53.5%)<br>9.- Leaves home using crutches-cane. n=25 (19.7%)<br>10.- Leaves home using a wheelchair. n=8 (6.3%)<br>11.- Leaves home using a walker. n=9 (7.1%)<br>12.- Leaves home and uses more than one mobility aid. n=26 (20.5%)<br>13.- Lives homebound (14+15). n=45 (35.4%)<br>14.- Homebound and does not use mobility aids. n=11 (8.7%)<br>15.- Lives homebound and uses more than one mobility aid in the home. n=34 (26.8%)<br>16.- Survival. n=75 (59.1%)<br>17.- Mortality. n=52 (40.9%)
**Networks (%)**
Economic income greater than 11.200 euros/year - Survival 70.1%
Economic income greater than 11.200 euros/year - Mortality 29.9%
Economic income less than 11.200 euros/year - Survival 46.7%
Economic income less than 11.200 euros/year - Mortality 53.3%
Moderate ADL-dependence - Survival 69.7%
Moderate ADL-dependence - Mortality 30.3%
Severe ADL-dependency - Survival 34.2%
Severe ADL-dependency - Mortality 65.8%
Leaves home without the need for mobility aids - Survival 85.7%
Leaves home without the need for mobility aids - Mortality 14.3%
Homebound and does not use mobility aids - Survival 27.3%
Homebound and does not use mobility aids - Mortality 72.7%
Leaves home using crutches-cane - Survival 72%
Leaves home using crutches-cane - Mortality 28%
Leaves home using a wheelchair - Survival 25%
Leaves home using a wheelchair - Mortality 75%
Leaves home using a walker - Survival 88.9%
Leaves home using a walker - Mortality 11.1%
Use more than one mobility aid when leaving the home - Survival 61.8%
Use more than one mobility aid when leaving the home - Mortality 38.2%
Lives homebound and uses more than one mobility aid in the home - Survival 52.9%
Lives homebound and uses more than one mobility aid in the home - Mortality 47.1%
Leaving the house and using a mobility aid - Survival 61.8%
Leaving the house and using a mobility aid - Mortality 38.2%
Leaving home - Leaving the house and using a mobility aid 51.2%
Leaving home - Leaves home without the need for mobility aids 17.5%
Leaving home - Use more than one mobility aid when leaving the home 31.7%

Mobility - Leaves home using crutches-cane 24.5%
Mobility - Leaves home using a wheelchair 7.8%
Mobility - Leaves home using a walker 8.8%
Mobility - Use more than one mobility aid when leaving the home 25.5%
Mobility - Lives homebound and uses more than one mobility aid in the home 33.3%
Leaving home - Leaving the house and using a mobility aid 82.9%
Leaves homebound - Lives homebound and uses more than one mobility aid in the home 75.5%
Leaves homebound - Homebound and does not use mobility aids 24.5%
Economic income greater than 11.200 euros/year - Leaving home 68.7%
Economic income greater than 11.200 euros/year - Lives homebound 31.3%
Economic income less than 11.200 euros/year - Leaving home 60%
Economic income less than 11.200 euros/year - Leaves homebound 40%
Moderate ADL-dependency – Lives homebound 28.1%
Severe ADL-dependency – Lives homebound 52.6%
Moderate ADL-dependency – Leaving home 71.9%
Severe ADL-dependency – Leaving home 47.4%
Mobility - Leaves home without the need for mobility aids 12.1%

Figure 4: Level of functional dependence and availability of assistants.

Availability of assistants for housework or live-in caregivers depending on the level of dependency. Coexistence of different types of assistants in relation to the level of dependency.

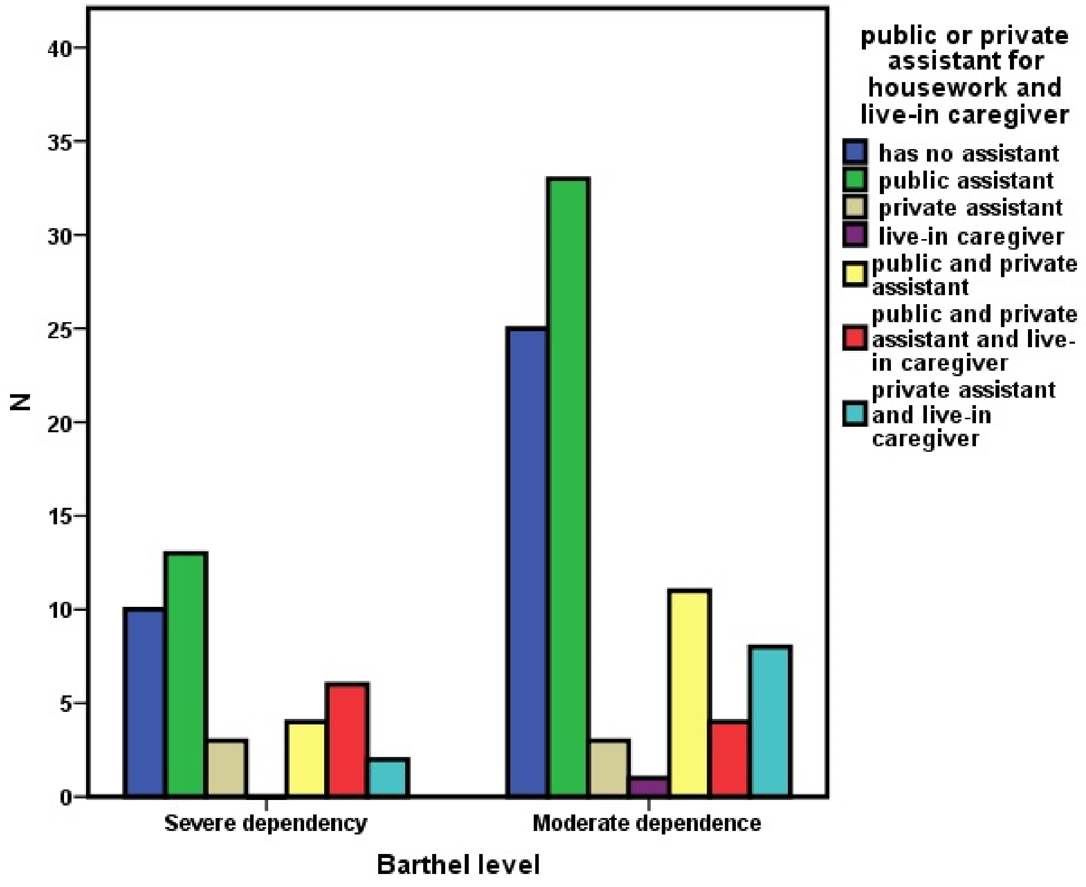

Contingency table public. private or internal caregiver * Barthel level before confinement

			Barthel level before confinement				Total
			Barthel < 20	Barthel 20-35	Barthel 40-55	Barthel 60	
public. private or internal caregiver	Not assistant	Count	2	8	12	13	35
		Expected frequency	5.4	5.4	13.1	11.1	35.0
		% within public. private or internal caregiver	5.7%	22.9%	34.3%	37.1%	100.0 %
		% within Barthel level before confinement	10.5%	42.1%	26.1%	33.3%	28.5%
	only has a public assistant caregiver	Count	6	7	17	16	46
		Expected frequency	7.1	7.1	17.2	14.6	46.0
		% within public. private or internal caregiver	13.0%	15.2%	37.0%	34.8%	100.0 %
		% within Barthel level before confinement	31.6%	36.8%	37.0%	41.0%	37.4%
	only has a private caregiver	Count	2	1	2	1	6
		Expected frequency	.9	.9	2.2	1.9	6.0
		% within public. private or internal caregiver	33.3%	16.7%	33.3%	16.7%	100.0 %
		% within Barthel level before confinement	10.5%	5.3%	4.3%	2.6%	4.9%
	only has an internal caregiver	Count	0	0	0	1	1
		Expected frequency	.2	.2	.4	.3	1.0
		% within public. private or internal caregiver	0.0%	0.0%	0.0%	100.0%	100.0 %
		% within Barthel level before confinement	0.0%	0.0%	0.0%	2.6%	.8%
		Count	3	1	6	5	15

	has public and private caregiver	Expected frequency	2.3	2.3	5.6	4.8	15.0
		% within public. private or internal caregiver	20.0%	6.7%	40.0%	33.3%	100.0 %
		% within Barthel level before confinement	15.8%	5.3%	13.0%	12.8%	12.2%
	has public. private and internal caregivers	Count	5	1	3	1	10
		Expected frequency	1.5	1.5	3.7	3.2	10.0
		% within public. private or internal caregiver	50.0%	10.0%	30.0%	10.0%	100.0 %
		% within Barthel level before confinement	26.3%	5.3%	6.5%	2.6%	8.1%
	has private and internal caregiver	Count	1	1	6	2	10
		Expected frequency	1.5	1.5	3.7	3.2	10.0
		% within public. private or internal caregiver	10.0%	10.0%	60.0%	20.0%	100.0 %
		% within Barthel level before confinement	5.3%	5.3%	13.0%	5.1%	8.1%
Total		Count	19	19	46	39	123
		Expected frequency	19.0	19.0	46.0	39.0	123.0
		% within public. private or internal caregiver	15.4%	15.4%	37.4%	31.7%	100.0 %
		% within Barthel level before confinement	100.0%	100.0%	100.0%	100.0%	100.0 %

Figure 5: Level of functional ADL-dependency and weekly hours of public assistance for housework.

Assistants assigned for housework by social services. Number of hours per week for each level of dependence on the Barthel index.

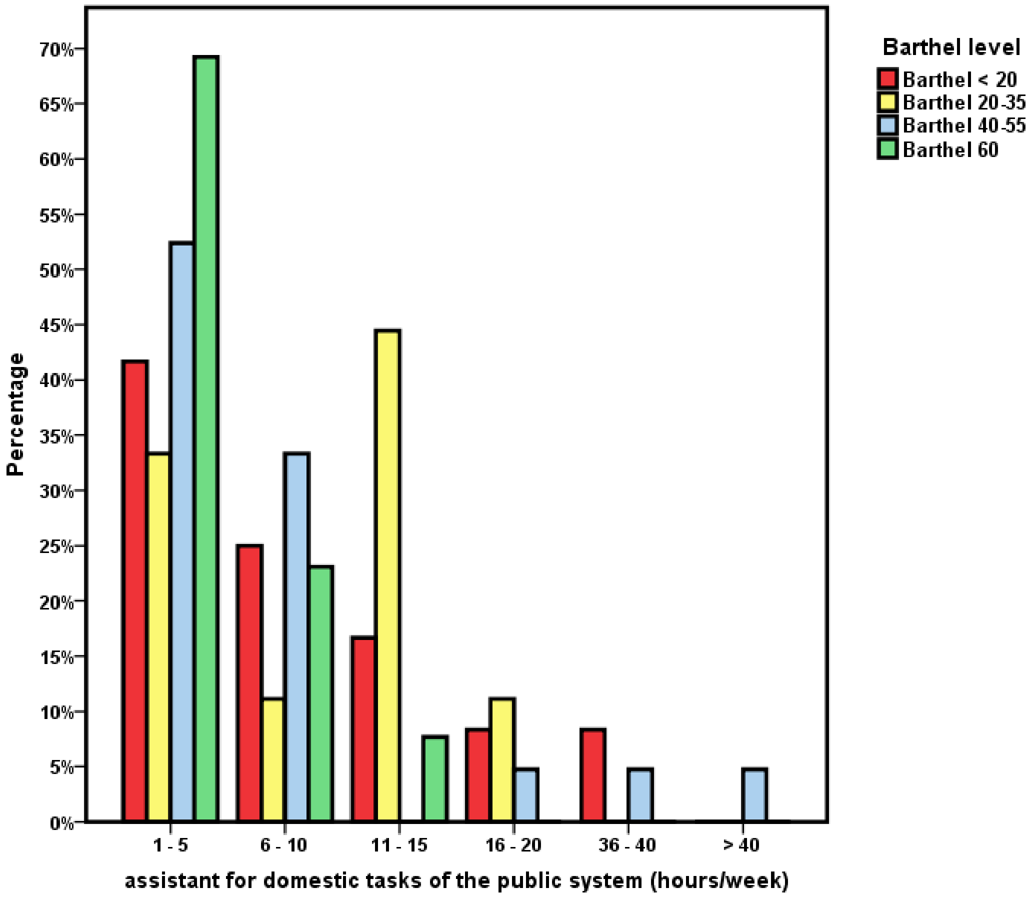

Contingency table hours of public assistant for housework per week * Barthel level before confinement

			Barthel level before confinement				Total
			Barthel < 20	Barthel 20-35	Barthel 40-55	Barthel 60	
hours of public assistant hours for housework per week	Not assistant	Count	7	10	25	17	59
		Expected frequency	8.8	8.8	21.4	20.0	59.0
		% within public assistant hours for housework per week	11.9%	16..9%	42.4%	28.8%	100.0%
		within % within Barthel level before confinement	36.8%	52.6%	54.3%	39.5%	46.5%
	1 - 5 hours	Count	5	3	11	18	37
		Expected frequency	5.5	5.5	13.4	12.5	37.0
		% within public assistant hours for housework per week	13..5%	8.1%	29.7%	48.6%	100.0%
		within % within Barthel level before confinement	26.3%	15.8%	23.9%	41.9%	29.1%
	6 - 10 hours	Recuento	3	1	7	6	17
		Expected frequency	2.5	2.5	6.2	5.8	17.0
		% within public assistant hours for housework per week	17.6%	5..9%	41.2%	35.3%	100.0%
		within % within Barthel level before confinement	15.8%	5.3%	15.2%	14.0%	13.4%
	11 - 15 hours	Count	2	4	0	2	8
		Expected frequency	1.2	1.2	2.9	2.7	8.0
		% within public assistant hours for housework per week	25.0%	50.0%	0.0%	25.0%	100.0%
		within % within Barthel level before confinement	10.5%	21.1%	0.0%	4.7%	6.3%
	16 - 20 hours	Count	1	1	1	0	3
		Expected frequency	.4	.4	1.1	1.0	3.0

		% within public assistant hours for housework per week	33.3%	33.3%	33.3%	0.0%	100.0%
		within % within Barthel level before confinement	5.3%	5.3%	2.2%	0.0%	2.4%
	36 - 39 hours	Count	1	0	1	0	2
		Expected frequency	.3	.3	.7	.7	2.0
		% within public assistant hours for housework per week	50.0%	0.0%	50.0%	0.0%	100.0%
		within % within Barthel level before confinement	5.3%	0.0%	2.2%	0.0%	1.6%
	>40 hours	Count	0	0	1	0	1
		Expected frequency	.1	.1	.4	.3	1.0
		% within public assistant hours for housework per week	0.0%	0.0%	100.0%	0.0%	100.0%
		within % within Barthel level before confinement	0.0%	0.0%	2.2%	0.0%	.8%
		Count	19	19	46	43	127
		Expected frequency	19.0	19.0	46.0	43.0	127.0
		% within public assistant hours for housework per week	15.0%	15.0%	36.2%	33.9%	100.0%
		within % within Barthel level before confinement	100.0%	100.0%	100.0%	100.0%	100.0%

Figure 6: Survival-mortality network analysis for chronic diseases.

Survival-mortality network analysis in relation to the chronic diseases present in the population with functional dependence of the Orcasitas cohort, according to living homebound or having the ability to leave home. Influence of the level of functional dependence and economic level on survival in both groups.

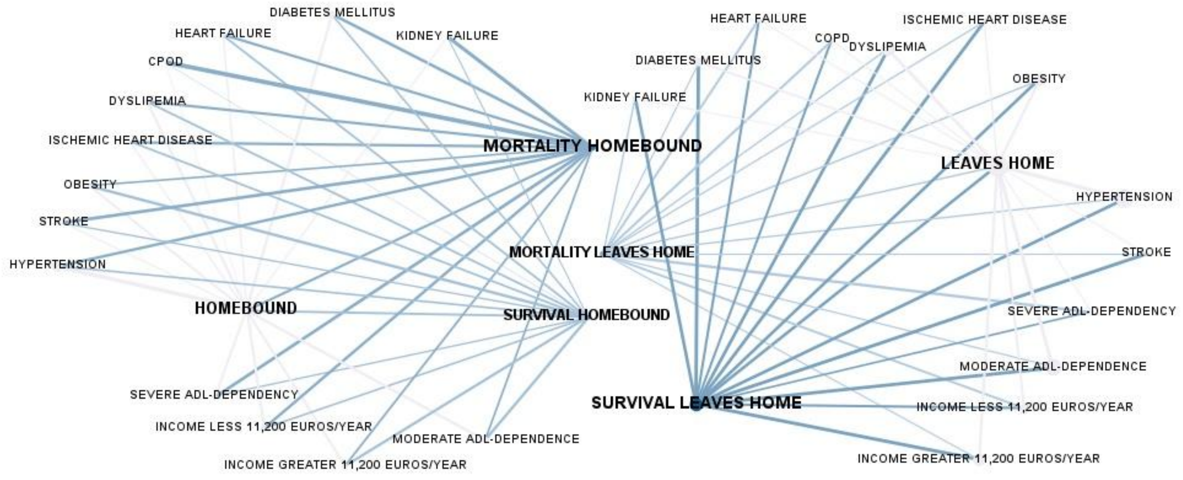

Source, Id, Label, Weight
**Nodes n (%)**
1.- Leaves home and Income greater 11.200 euros/year. n= 46 (36.2%)<br>2.- Leaves home and Income less than 11.200 euros/year. n=36 (28.3%)<br>3.- Homebound and Income greater 11.200 euros/year. n= 21 (16.5%)<br>4.- Homebound and Income less than 11.200 euros/year. n=24 (18.9%)<br>5.- Leaves home and Moderate ADL-dependence. n=64 (50.4%)<br>6.- Leaves home and Severe ADL-dependency. n=18 (14.2%)<br>7.- Homebound and Moderate ADL-dependence. n=25 (19.7%)<br>8.- Homebound and Severe ADL-dependency. n=20 (15.7%)<br>9.- Leaving home. n=82 (64.6%)<br>10.- Homebound. n=45 (35.4%)<br>11.- Leaves home and Survival. n=54 (42.5%)<br>12.- Leaves home and Mortality. n=28 (22.1%)<br>13.- Homebound and Survival. n=21 (16.5%)<br>14.- Homebound and Mortality. n=24 (18.9%)<br>15.- Leaves home and Diabetes Mellitus. n=33 (26%)<br>16.- Leaves home and Hypertension. n=71 (55.9%)<br>17.- Leaves home and Dyslipemia. n=56 (44.1%)<br>18.- Leaves home and Obesity. n=43 (33.9%)<br>19.- Leaves home and COPD. n=16 (12.6%)<br>20.- Leaves home and Heart failure. n=19 (15%)<br>21.- Leaves home and ischemic heart disease. n=21 (16.5%)<br>22.- Leave home and Stroke. n=16 (12.6%)<br>23.- Leaves home and Kidney failure. n=16 (12.6%)<br>15.- Homebound and Diabetes Mellitus. n=23 (18.1%)<br>16.- Homebound and Hypertension. n=41 (32.3%)

17.- Homebound and Dyslipemia. n=23 (18.1%)<br>18.- Homebound and Obesity. n=21 (16.5%)<br>19.- Homebound and COPD. n=4 (3.1%)<br>20.- Homebound and Heart failure. n=14 (11%)<br>21.- Homebound and Ischemic heart disease. n=12 (9.4%)<br>22.- Homebound and Stroke. n=10 (7.9%)<br>23.- Homebound and Kidney failure. n=10 (7.9%)
**Networks (%)**
Leaves home - SURVIVAL 65.9%
Leaves home – MORTALITY 34.1%
Homebound – SURVIVAL 46.7%
Homebound – MORTALITY 53.3%
Income greater than 11,200 euros/year – Leaves home SURVIVAL 76.1%
Income greater than 11,200 euros/year – Leaves home MORTALITY 23.9%
Income less than 11,200 euros/year – Leaves home SURVIVAL 52.8%
Income less than 11,200 euros/year – Leaves home MORTALITY 47.2%
Income greater than 11,200 euros/year – Homebound MORTALITY 42.9%
Income greater than 11,200 euros/year – Homebound SURVIVAL 57.1%
Income less than 11,200 euros/year – Homebound SURVIVAL 37.5%
Income less than 11,200 euros/year – Homebound MORTALITY 62.5%
Moderate ADL-dependency – Leaves home SURVIVAL 73.4%
Moderate ADL-dependency – Leaves home MORTALITY 26.6%
Severe ADL-dependency – Leaves home SURVIVAL 38.9%
Severe ADL-dependency - Leaves home MORTALITY 61.1%
DIABETES MELLITUS - Leaves home SURVIVAL 75.8%
DIABETES MELLITUS - Leaves home MORTALITY 24.2%
HYPERTENSION - Leaves home SURVIVAL 69%
HYPERTENSION -– Leaves home MORTALITY 31%
DYSLIPEMIA – Leaves home SURVIVAL 69.6%
DYSLIPEMIA – Leaves home MORTALITY 30.4%
OBESITY – Leaves home SURVIVAL 67.4%
OBESITY – Leaves home MORTALITY 32.6%
COPD – Leaves home SURVIVAL 50%

COPD – Leaves home MORTALITY 50%
HEART FAILURE – Leaves home SURVIVAL 52.6%
HEART FAILURE – Leaves home MORTALITY 47.4%
ISCHEMIC HEART DISEASE – Leaves home SURVIVAL 71.4%
ISCHEMIC HEART DISEASE – Leaves home MORTALITY 28.6%
STROKE – Leaves home SURVIVAL 75%
STROKE – Leaves home MORTALITY 25%
KIDNEY FAILURE – Leaves home SURVIVAL 68.8%
KIDNEY FAILURE – Leaves home MORTALITY 31.3%
Moderate ADL-dependence – Homebound Survival 60%
Moderate ADL-dependence – Homebound Mortality 40%
Severe ADL-dependency – Homebound Survival 30%
Severe ADL-dependency – Homebound Mortality 70%
Homebound – Homebound Survival 46.7%
Homebound – Homebound Mortality 53.3%
Homebound - Income greater than 11,200 euros/year 46.7%
Homebound - Income less than 11,200 euros/year 53.3.%
Leaves home - Income greater than 11,200 euros/year 56.1%
Leaves home - Income less than 11,200 euros/year 43.9%
Homebound – Moderate ADL-dependence 55.6%
Homebound – Severe ADL-dependency 44.4%
Leaves home - Moderate ADL-dependence 78%
Leaves home - Severe ADL-dependency 22%
Leaves home - DIABETES MELLITUS 40.2%
Leaves home – HYPERTENSION 86.6%
Leaves home – DYSLIPEMIA 68.3%
Leaves home – OBESITY 52.4%
Leaves home – COPD 19.5%
Leaves home - HEART FAILURE 23.3%
Leaves home - ISCHEMIC HEART DISEASE 25.6%

Leaves home – STROKE 19.5%
Leaves home - KIDNEY FAILURE 19.5%
Homebound – KIDNEY FAILURE 22.2%
Homebound – DYSLIPEMIA 51.1%
Homebound – OBESITY 46.7%
Homebound – HEART FAILURE 31.1%
Homebound – HYPERTENSION 84.4%
Homebound – DIABETES MELLITUS 51.1%
Homebound – STROKE 22.2%
Homebound – CPOD 8.9%
Homebound – ISCHEMIC HEART DISEASE 26.7%
HEART FAILURE – SURVIVAL Homebound 42.9%
HEART FAILURE – MORTALITY Homebound 57.1%
OBESITY – MORTALITY Homebound 38.1%
OBESITY – SURVIVAL Homebound 61.9%
DYSLIPEMIA – SURVIVAL Homebound 43.5%
DYSLIPEMIA – MORTALITY Homebound 56.5%
ISCHEMIC HEART DISEASE – SURVIVAL Homebound 41.7%
ISCHEMIC HEART DISEASE – Homebound MORTALITY 58.3%
KYDNEY FAILURE – SURVIVAL Homebound 20%
KYDNEY FAILURE – MORTALITY Homebound 80%
HYPERTENSION – MORTALITY Homebound 60.5%
HYPERTENSIÓN – SURVIVAL Homebound 39.5%
STROKE – MORTALITY Homebound 70%
STROKE – SURVIVAL Homebound 30%
CPOD – MORTALITY Homebound 100%
CPOD – SURVIVAL Homebound 0%
DIABETES MELLITUS – SURVIVAL Homebound 34.8%
DIABETES MELLITUS – Homebound MORTALITY 65.2%

Figure 7: Cox regression: mortality risk associated with income level in wheelchair users.

Association between level of economic income and survival at three years of follow-up in persons with functional ADL-dependence who were wheelchair users.

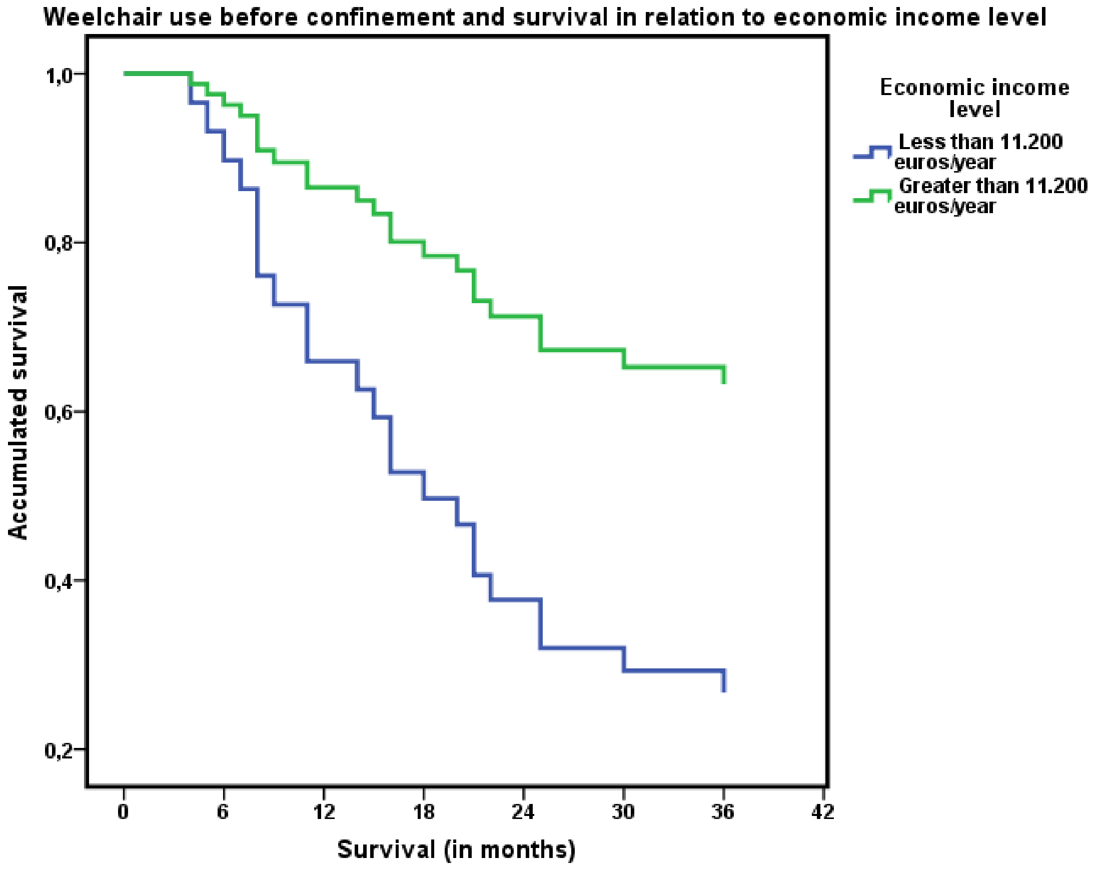

Omnibus tests on model coefficients[a]

-2 log likelihood	Global (score)			Change from previous step			Change from previous block		
	Chi-square	gl	Sig.	Chi-square	gl	Sig.	Chi-square	gl	Sig.
149.852	5.860	1	0.015	5.898	1	0.015	5.898	1	0.015

a. Initial block number 1. Method = Enter

Variables in the equation

	B	ET	Wald	gl	Sig.	Exp(B)	95.0% CI for Exp(B)	
							Lower	Upper
Economic income level	1.057	0.456	5.366	1	0.021	2.878	1.177	7.038

Figure 8. panel A: Cox regression: mortality risk associated with mode of living in the community.

Survival at three years of follow-up in relation to having pre-pandemic Covid-19 confinement ability to leave home.

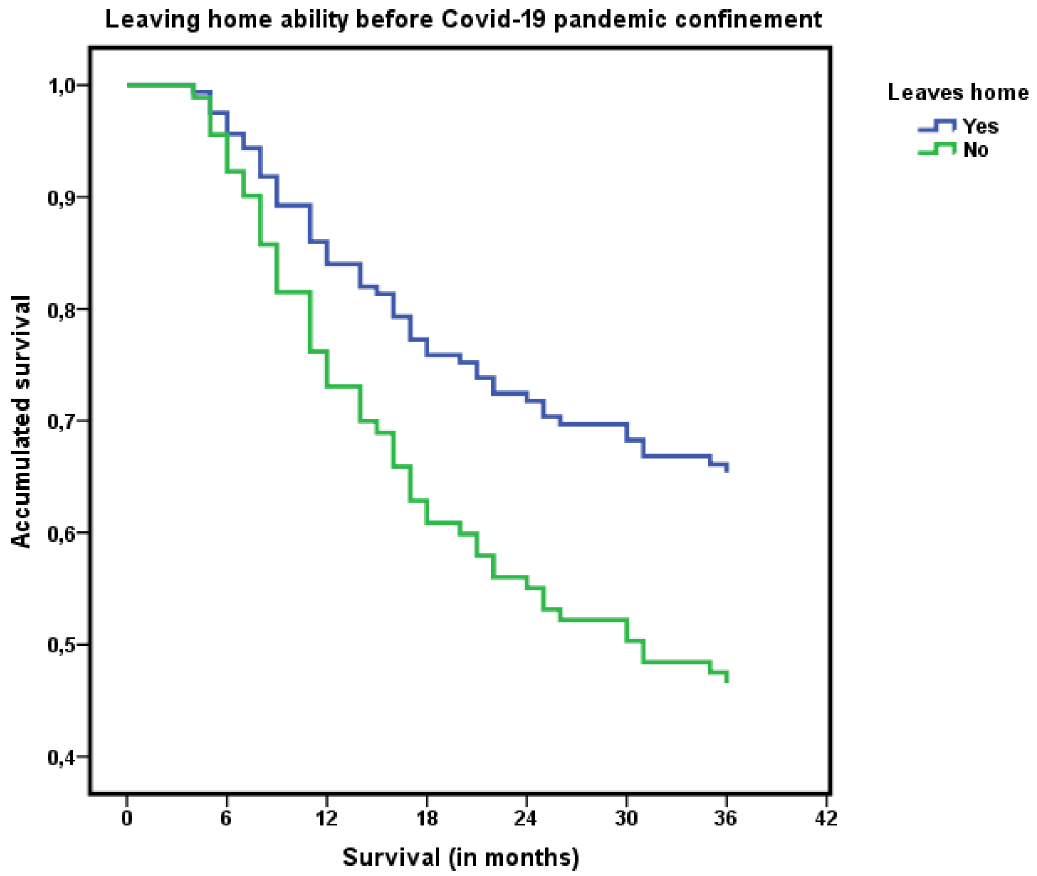

Omnibus tests on model coefficients[a]

	Global (score)			Change from previous step			Change from previous block		
-2 log likelihood	Chi-square	gl	Sig.	Chi-square	gl	Sig.	Chi-square	gl	Sig.
474.852	4.575	1	0.032	4.321	1	0.038	4.321	1	0.038

a. Initial block number 1. Method = Enter

Variables in the equation

							95.0% CI for Exp(B)	
	B	ET	Wald	gl	Sig.	Exp(B)	Lower	Upper
Leaves home before confinement	-0.587	0.278	4.446	1	0.035	0.556	0.322	0.959

Figure 8. panel B: Cox regression: mortality risk associated with mode of living in the community.

Survival at three years follow-up in relation to maintaining after Covid-19 pandemic confinement the ability to leave home.

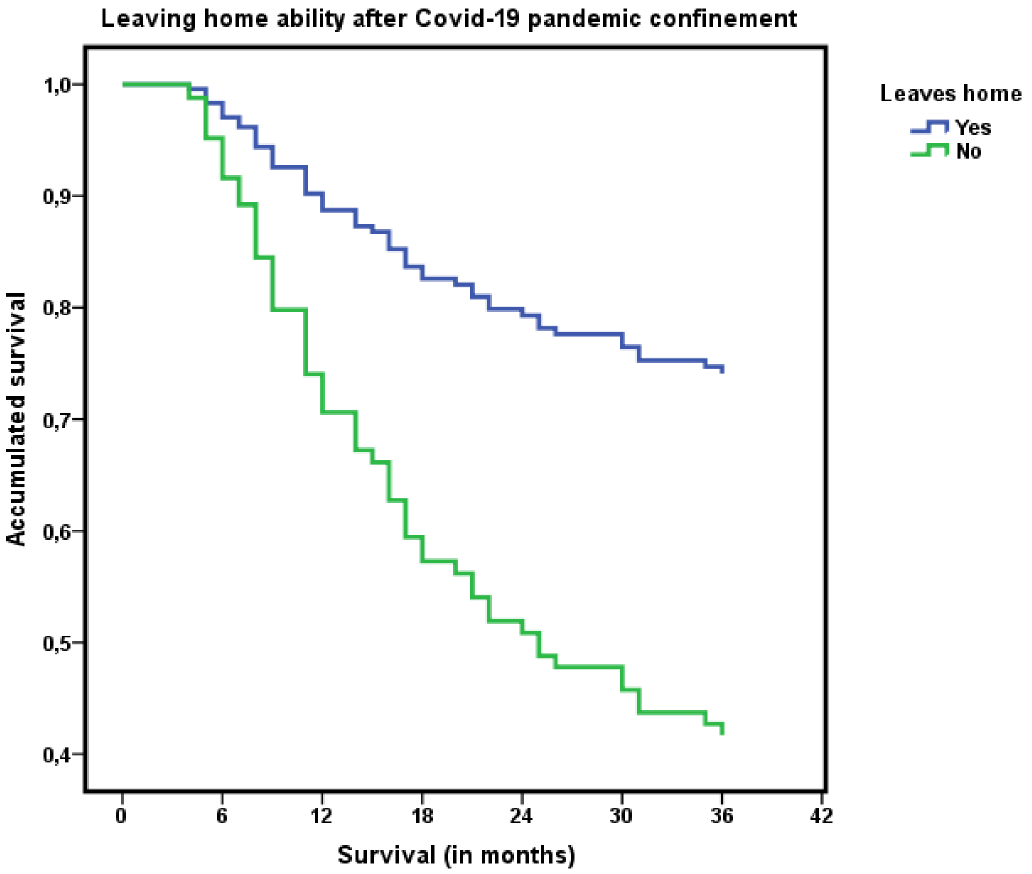

Omnibus tests on model coefficients[a]

	Global (score)			Change from previous step			Change from previous block		
-2 log likelihood	Chi-square	gl	Sig.	Chi-square	gl	Sig.	Chi-square	gl	Sig.
465.084	14.282	1	0.000	14.089	1	0.000	14.089	1	0.000

a. Initial block number 1. Method = Enter

Variables en la ecuación

							95.0% IC para Exp(B)	
	B	ET	Wald	gl	Sig.	Exp(B)	Inferior	Superior
Leaves home after confinement	-1.070	0.297	13.018	1	0.000	0.343	0.192	0.613

Figure 9: Influence of economic capacity on the mode of living in the community.

Interrelationships between the ability to leave the house before and after confinement. the level of economic income and the level of dependency.

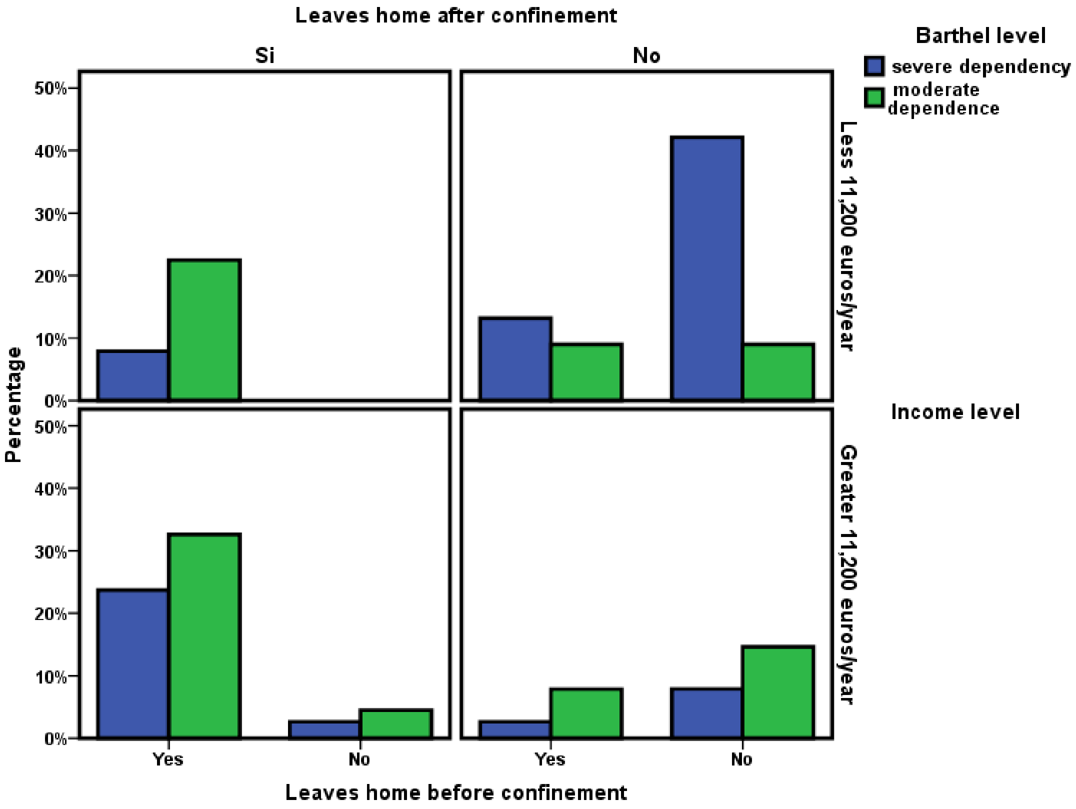

SPSS does not provide the results in tabular form to produce these graphs. We have provided the partial analyses as information. which also allow the graph to be interpreted correctly:

Barthel analysis severe dependency - financial income less than 11.200 euros/year.

Contingency table Leaves home before confinement – Leaves home after confinement

			Leaves home after confinement		Total
			Yes	No	
Leaves home before confinement	Yes	Count	3	5	8
		Expected frequency	1.0	7.0	8.0
		% inside of Leaves home before confinement	37.5%	62.5%	100.0%
		% inside Leaves home after confinement	100.0%	23.8%	33.3%
	No	Count	0	16	16
		Expected frequency	2.0	14.0	16.0
		% inside of Leaves home before confinement	0.0%	100.0%	100.0%
		% inside of Leaves home after confinement	0.0%	76.2%	66.7%
Total		Count	3	21	24
		Expected frequency	3.0	21.0	24.0
		% inside of Leaves home before confinement	12.5%	87.5%	100.0%
		% inside of Leaves home after confinement	100.0%	100.0%	100.0%

Chi-square tests

	Value	gl	Asymptotic sig. (bilateral)	Exact sig. (bilateral)	Exact sig. (unilateral)
Pearson's Chi-square	6.857[a]	1	0.009		
Continuity correction[b]	3.857	1	0.050		
Likelihood ratio	7.500	1	0.006		
Fisher's exact statistic				0.028	0.028
Linear by linear association	6.571	1	0.010		
N of valid cases	24				

a. 2 cells (50%) have an expected frequency of less than 5. The minimum expected frequency is 1.00.

b. Calculated only for a 2x2 table.

Barthel analysis severe dependency - income more than 11.200 euros/year.

Contingency table Leaves home before confinement – Leaves home after confinement

			Leaves home after confinement		Total
			Yes	No	
Leaves home before confinement	Yes	Count	9	1	10
		Expected frequency	7.1	2.9	10.0
		% within Leaves home before confinement	90.0%	10.0%	100.0%
		% within Leaves home after confinement	90.0%	25.0%	71.4%
	No	Count	1	3	4
		Expected frequency	2.9	1.1	4.0
		% within Leaves home before confinement	25.0%	75.0%	100.0%
		% within Leaves home after confinement	10.0%	75.0%	28.6%
Total		Count	10	4	14
		Expected frequency	10.0	4.0	14.0
		% within Leaves home before confinement	71.4%	28..6%	100.0%
		% within Leaves home after confinement	100.0%	100.0%	100.0%

Chi-square tests

	Value	gl	Asymptotic sig. (bilateral)	Exact sig. (bilateral)	Exact sig. (unilateral)
Pearson's Chi-square	5.915[a]	1	0.015		
Continuity correction[b]	3.159	1	0.076		
Likelihood ratio	5.751	1	0.016		
Fisher's exact statistic				0.041	0.041
Linear by linear association	5.493	1	0.019		
N of valid cases	14				

a. 3 cells (75%) have an expected frequency of less than 5. The minimum expected frequency is 1.14.

b. Calculated only for a 2x2 table.

Barthel analysis moderate dependency - income higher than 11.200 euros/year.

Contingency table Leaves home before confinement – Leaves home after confinement

			Leaves home after confinement		Total
			Yes	No	
Leaves home before confinement	Yes	Count	29	7	36
		Expected frequency	22.4	13.6	36.0
		% within Leaves home before confinement	80.6%	19.4%	100.0%
		% within Leaves home after confinement	87.9%	35.0%	67.9%
	No	Count	4	13	17
		Expected frequency	10.6	6.4	17..0
		% within Leaves home before confinement	23.5%	76.5%	100.0%
		% within Leaves home after confinement	12.1%	65.0%	32.1%
Total		Count	33	20	53
		Expected frequency	33.0	20.0	53.0
		% within Leaves home before confinement	62.3%	37.7%	100.0%
		% within Leaves home after confinement	100.0%	100.0%	100.0%

Chi-square tests

	Value	gl	Asymptotic sig. (bilateral)	Exact sig. (bilateral)	Exact sig. (unilateral)
Pearson's Chi-square	15.982[a]	1	0.000		
Continuity correction[b]	13.647	1	0.000		
Likelihood ratio	16.234	1	0.000		
Fisher's exact statistic				0.000	0.000
Linear by linear association	15.680	1	0.000		
N of valid cases	53				

a. 0 cells (.0%) have an expected frequency of less than 5. The minimum expected frequency is 6.42.

b. Calculated only for a 2x2 table.

Barthel analysis moderate dependency - income less than 11.200 euros/year.

Contingency table Leaves home before confinement – Leaves home after confinement

			Leaves home after confinement		Total
			Yes	No	
Leaves home before confinement	Yes	Count	20	8	28
		Expected frequency	15.6	12.4	28.0
		% within Leaves home before confinement	71.4%	28.6%	100.0%
		% within Leaves home after confinement	100.0%	50.0%	77.8%
	No	Count	0	8	8
		Expected frequency	4.4	3.6	8.0
		% within Leaves home before confinement	0.0%	100.0%	100.0%
		% within Leaves home after confinement	0.0%	50.0%	22.2%
Total		Count	20	16	36
		Expected frequency	20.0	16.0	36.0
		% within Leaves home before confinement	55.6%	44.4%	100.0%
		% within Leaves home after confinement	100.0%	100.0%	100.0%

Chi-square tests

	Value	gl	Asymptotic sig. (bilateral)	Exact sig. (bilateral)	Exact sig. (unilateral)
Pearson's Chi-square	12.857[a]	1	0.000		
Continuity correction[b]	10.127	1	0.001		
Likelihood ratio	15.958	1	0.000		
Fisher's exact statistic				0.000	0.000
Linear by linear association	12.500	1	0.000		
N of valid cases	36				

a. 2 cells (50%) have an expected frequency of less than 5. The minimum expected frequency is 3.56.

b. Calculated only for a 2x2 table.

Figure 10, panel A: Network analysis before Covid-19 confinement.

Network analysis of the interrelationships between level of dependence, economic level, and mode of living in the community of people with functional dependence in the Orcasitas cohort. Analysis of the baseline situation before confinement due to the Covid-19 pandemic and influence on survival at three years.

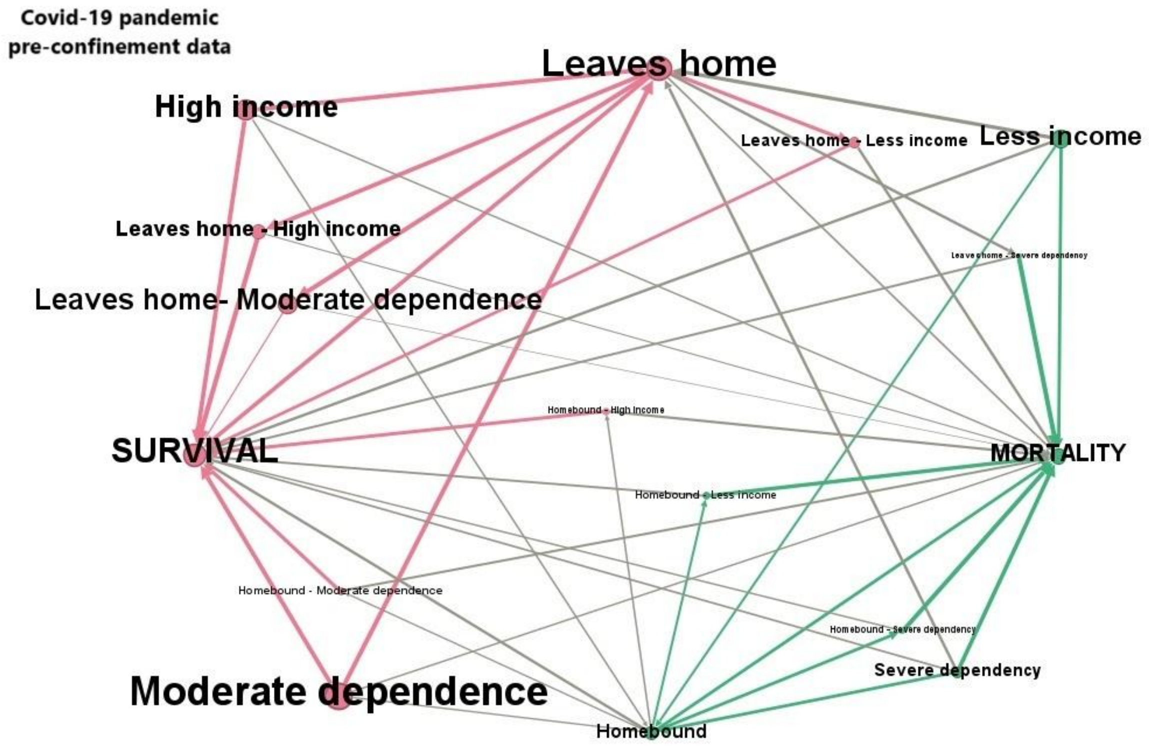

Source, Id, Label, Weight
**Nodes n (%)**
1.- High income. n= 67 (52.8%)<br>2.- Less income. n=60 (47.2%)<br>3.- Moderate dependence. n=89 (70.1%)<br>4.- Severe dependency. n=38 (29.9%)<br>5.- Leaves home. n=82 (64.6%)<br>6.- Lives homebound. n=45 (35.4%)<br>7.- Survival. n=75 (59.1%)<br>8.- Mortality. n=52 (40.9%)<br>9.- Leaves home – Less income. n=36 (28.3%)<br>10.- Leaves home – High income. n=46 (36.2%)<br>11.- Homebound – Less income. n=24 (18.9%)<br>12.- Homebound – High income. n=21 (16.5%)<br>13.- Leaves home – Severe dependency. n=18 (14.2%)<br>14.- Leaves home – Moderate dependence. n=64 (50.4%)<br>15.- Homebound – Severe dependency. n=20 (15.7%)<br>16.- Homebound – Moderate dependence. n=25 (19.7%)

**Networks (%)**
Homebound – Survival 46.7%
Less income – Leaves home 60%
Less income – Homebound 40%
High income – Homebound 31.3%
High income – Leaves home 68.7%
Severe dependency – Leaves home 47.4%
Severe dependency – Homebound 52.6%
Moderate dependence – Homebound 28.1%
Moderate dependence – Leaves home 71.9%
Leaves home – Leaves home and less income 60%
Leaves home – Leaves home and high income 68.7%
Leaves home and less income – Survival 52.8%
Homebound – Homebound and les income 40%
Homebound – Homebound and high income 31.3%
Leaves home and less income – Mortality 47.2%
Leaves home and high income – Mortality 23.9%
Leaves home and high income – Survival 76.1%
Homebound and less income – Survival 37.5%
Homebound and les income – Mortality 62.5%
Homebound and high income – Mortality 42.9%
Homebound and high income – Survival 57.1%
Leaves home and moderate dependence – Mortality 17%
Leaves home and severe dependency – Survival 38.9%
Leaves home and severe dependency – Mortality 73.4%
Leaves home and moderate dependence – Survival 26.6%
Homebound and severe dependency – Survival 30%
Homebound and severe dependency – Mortality 70%
Homebound and moderate dependence – Mortality 40%
Homebound and moderate dependence – Survival 60%
Homebound – Homebound and moderate dependence 28.1%
Homebound – Homebound and severe dependency 52.6%
Leaves home – Leaves home and moderate dependence 71.9%
Leaves home – Leaves home and severe dependency 47.4%
Less income – Mortality 53.3%

Less income – Survival 46.7%
High income – Survival 70.1%
High income – Mortality 29.9%
Severe dependency – Mortality 65.8%
Severe dependency – Survival 34.2%
Moderate dependence – Survival 69.7%
Moderate dependence – Mortality 30.3%

Figure 10, panel B: Network analysis after Covid-19 confinement.

Network analysis of the interrelationships between level of dependency, economic status, and mode of living in the community after Covid-19 pandemic confinement and their influence on survival at three years. Analysis of changes associated with Covid-19 pandemic confinement.

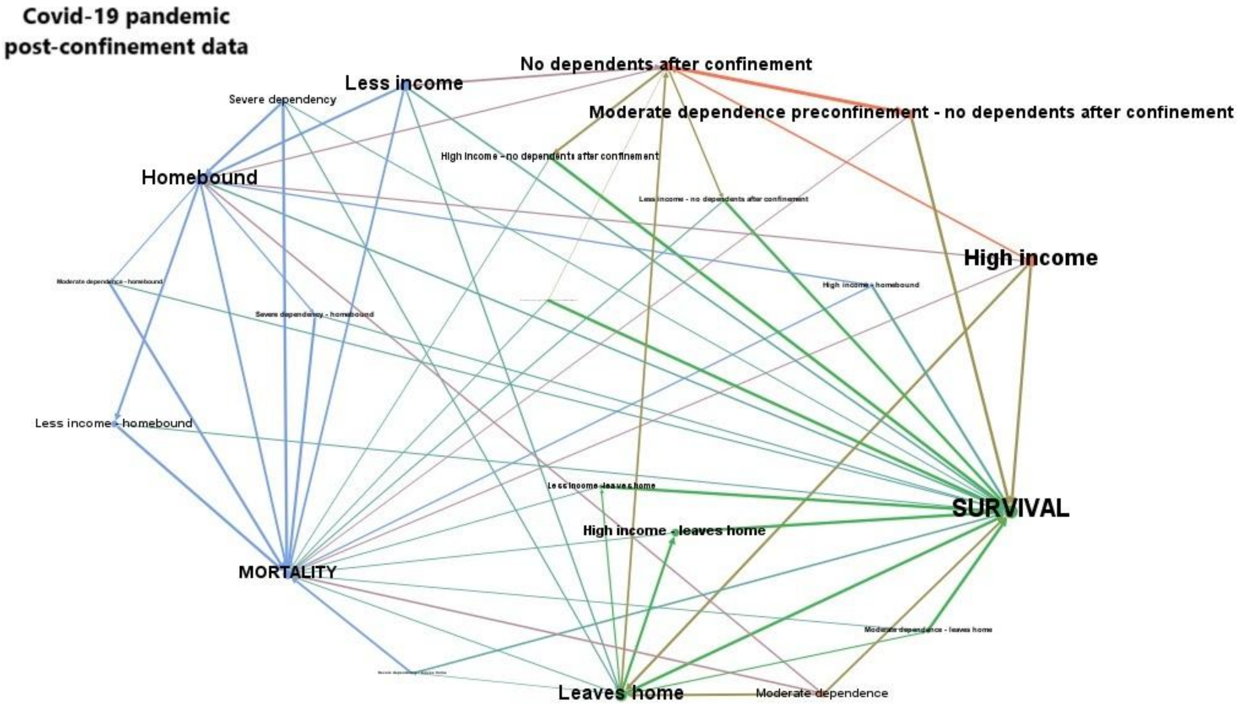

Source, Id, Label, Weight
**Nodes n (%)**
1.- High income. n= 67 (52.8%)<br>2.- Less income. n=60 (47.2%)<br>3.- Moderate dependence. n=39 (30.7%)<br>4.- Severe dependency. n=33 (26%)<br>5.- Not dependents after confinement. n=55 (43.3%)<br>5.- Leaves home post-confinement. n=66 (52%)<br>6.- Lives homebound post-confinement. n=61 (48%)<br>7.- Survival. n=75 (59.1%)<br>8.- Mortality. n=52 (40.9%)<br>9.- Less income - Leaves home. n=25 (19.7%)<br>10.- Less income - Homebound. n=37 (29.1%)<br>11.- High income - Leaves home. n=43 (33.8%)<br>12.- High income - Homebound. n=24 (18.9%)<br>13.- Severe dependency - Leaves home. n=11 (8.7%)<br>14.- Moderate dependence - Leaves home. n=21 (16.5%)<br>15.- Severe dependency - homebound. n=22 (17.3%)

16.- Moderate dependence - Homebound. n=18 (14.2%)<br>17.- Less income – Not dependents after confinement. n=23 (18.1%)<br>18.- High income – Not dependents after confinement. n=32 (25.2%)<br>19.- Severe dependency – Not dependents after confinement. n=4 (3.1%)<br>20.- Moderate dependence – Not dependents after confinement. n=51 (40.2%)
**Networks (%)**
Leaves home- Survival 74.2%
Leaves home- Mortality 25.8%
Homebound - Mortality 57.4%
Homebound – Survival 42.6%
Less income – Leaves home 38.3%
Less income – Homebound 61.7%
High income – Leaves home 64.2%
High income – Homebound 35.8%
High income – No dependents after confinement 46.2%
Less income – No dependents after confinement 53.8%
High income – Mortality 52.9%
High income – Survival 47.1%
Less income – Mortality 75%
Less income – Survival 25%
Severe dependency – Leaves home 34.2%
Severe dependency – Homebound 65.8%
Moderate dependence – Homebound 40.4%
Moderate dependence – Leaves home 59.6%
Less income and leaves home – Survival 73.9%
Less income and leaves home – Mortality 26.1%
Less income and homebound – Mortality 70.3%
Less income and Homebound – Survival 29.7%
High income and leaves home – Survival 74.4%
High income and leaves home – Mortality 25.6%
High income and homebound – Mortality 37.5%
High income and homebound – Survival 62.5%
Severe dependency and leaves home – Survival 45.5%
Severe dependency and leaves home – Mortality 54.5%
Severe dependency and homebound – Mortality 68.2%
Severe dependency and homebound – Survival 31.8%
Moderate dependence and leaves home – Survival 76.2%
Moderate dependence and leaves home – Mortality 23.8%
Moderate dependence and homebound – Mortality 66.7%
Moderate dependence and homebound – Survival 33.3%
Less income and no dependents after confinement – Survival 69.6%
Less income and no dependents after confinement – Mortality 30.4%
High income and no dependents after confinement – Mortality 21.9%
High income and no dependent after confinement – Survival 78.1%
Severe dependency pre-confinement and no dependents after confinement – Survival 75%

Severe dependency pre-confinement and no dependents after confinement – Mortality 25%
Moderate dependence pre-confinement and no dependents after confinement – Mortality 24.5%
Moderate dependence pre-confinement and no dependents after confinement – Survival 74.5%
No dependents after confinement – Less income and no dependents after confinement 41.8%
No dependents after confinement – High income and no dependents after confinement 58.2%
No dependents after confinement – Severe dependency pre-confinement and no dependents after confinement 7.3%
No dependents after confinement – Moderate dependence pre-confinement and no dependents after confinement 92.7%
Leaves home – Severe dependency and leaves home 16.7%
Leaves home – Moderate dependence and leaves home 31.8%
Leaves home – No dependents after confinement 51.5%
Homebound – Severe dependency and homebound 36.1%
Homebound – Moderate dependence and homebound 29.5%
Homebound – No dependents after confinement 34.4%
Homebound – Less income and homebound 60.7%
Homebound – High income and homebound 39.3%
Leaves home – Less income and leaves home 34.8%
Leaves home – High income and leaves home 65.2%
Severe dependency – Mortality 72.7%
Severe dependence – Survival 27.3%
Moderate dependence – Survival 54.5%
Moderate dependence – Mortality 44.5%
