# Supplementary material for: Socio-health factors, ability to perform instrumental and basic activities of daily living, and use of assistive mobility devices during the COVID-19 pandemic: Interrelationships and impact on long-term survival
Source: PLoS One. 2025 May 19;20(5):e0318481. doi: 10.1371/journal.pone.0318481 (PMC12088529; doi:10.1371/journal.pone.0318481)
Supplement: S2 Text — (PDF) [file pone.0318481.s002.pdf]

Supplementary 2, “Tables associated with the study”

Socio-health factors, ability to perform instrumental and basic activities of daily living, and use of assistive mobility devices during the Covid-19 pandemic. Interrelationships and impact on long-term survival.

Authors:

Vicente Martín Moreno. María Inmaculada Martínez Sanz. Irene Sánchez González. Miguel Recuero Vázquez. Sara Guerra Maroto. Miriam Fernández Gallardo. Amanda Martín Fernández. Julia Herranz Hernando. María Palma Benítez Calderón. Eva Sevillano Fuentes. Elena Pérez Rico. Laura Calderón Jiménez. Elena Sánchez Rodríguez. Helena Alonso Samperiz. Irene León Saiz. On behalf of GIDO collaborative group (Orcasitas Dependency Research Group). Juana Marcos Guerra.

Address for correspondence:

Vicente Martín Moreno

e-mail: amanvic@hotmail.com

Table 1s: Survival at 3-year follow-up among persons with functional dependence in the Orcasitas cohort, in relation to community living, the need to use a mobility assistance device, and the availability of a public or private assistant to perform housework, or of an internal caregiver. Results relative to the baseline situation of the cohort, before confinement due to the Covid-19 pandemic. 1: The number reflecting each variable corresponds to the number of people who responded to both variables. For privacy, a small number of people did not respond.

Availability of assistants for housework, use of mobility aids, ability to leave home, and three years survival.				
**Assistants for the performance of housework and personal care**				
		**SURVIVAL**		
**Variable[1]**	**n (%)**	**DECEASED**	**ODDS RATIO**	**CONFIDENCE INTERVAL**
Public assistant:<br>Yes<br>No	 70 (55.6%)<br>56 (44.4%)	 25 (48.1%)<br>27 (51.9%)	1.676	0.818-3.432
Private assistant:<br>Yes<br>No	 45 (36.3%)<br>79 (63.7%)	 21 (41.2%)<br>30 (58.8%)	0.700	0.333-1.468
Internal caregiver:<br>Yes<br>No	 24 (19.2%)<br>101 (80.8%)	 11 (21.2%)<br>41 (78.8%)	0.808	0.330-1.978
**Crutch-cane user before confinement due to Covid-19 pandemic (before March 2020)**				
Public assistant:<br>Yes<br>No	 30 (56.6%)<br>23 (43.4%)	 10 (55.6%)<br>8 (44.4%)	1.067	0.339-3.354
Private assistant:<br>Yes<br>No	 17 (32.7%)<br>35 (67.3%)	 7 (38.9%)<br>11 (61.1%)	0.655	0.197-2.176
Internal caregiver:<br>Yes<br>No	 8 (15.1%)<br>45 (84.9%)	 3 (16.7%)<br>15 (83.3%)	0.833	0.175-3.965
**Walker user before confinement due to Covid-19 pandemic (before March 2020)**				
Public assistant:<br>Yes<br>No	 23 (62.2%)<br>14 (37.8%)	 7 (53.8%)<br>6 (46.2%)	1.714	0.431-6.826
Private assistant:<br>Yes<br>No	 13 (36.1%)<br>23 (63.9%)	 3 (25%)<br>9 (75%)	2.143	0.460-9.977
Internal caregiver:<br>Yes<br>No	 9 (24.3%)<br>28 (75.7%)	 2 (15.4%)<br>11 (84.6%)	2.265	0.396-12.966
**Wheelchair user before confinement due to Covid-19 pandemic (before March 2020)**				
Public assistant:<br>Yes<br>No	 24 (58.5%)<br>17 (41.5%)	 14 (60.9%)<br>9 (19.1%)	0.804	0.230-2.808
Private assistant:<br>Yes<br>No	 16 (40%)<br>24 (60%)	 8 (36.4%)<br>14 (63.6%)	1.400	0.392-4.997
Internal caregiver:<br>Yes<br>No	 14 (34.1%)<br>27 (65.9%)	 7 (30.4%)<br>16 (69.6%)	1.455	0.397-5.331
**People who before the Covid-19 pandemic confinement could leave their home**				
Public assistant:<br>Yes<br>No	 48 (59.3%)<br>33 (40.7%)	 14 (29.2%)<br>14 (42.4%)	1.789	0.706-4.533
Private assistant:<br>Yes<br>No	 30 (37.5%)<br>50 (62.5%)	 13 (43.3%)<br>15 (30.0%)	0.560	0.218-1.438
Internal caregiver:<br>Yes<br>No	 17 (21.3%)<br>63 (78.8%)	 8 (47.1%)<br>20 (31.7%)	1.789	0.706-4.533
**People who before the Covid-19 pandemic were living homebound**				
Public assistant:<br>Yes<br>No	 22 (48.9%)<br>23 (50.1%)	 11 (50.8%)<br>13 (56.52%)	1.300	0.402-4.205
Private assistant:<br>Yes<br>No	 15 (34.1%)<br>29 (65.9%)	 8 (53.3%)<br>15 (51.7%)	0938	0.269-3.268
Internal caregiver:<br>Yes<br>No	 7 (15.6%)<br>38 (84.4%)	 3 (42.8%)<br>21 (55.3%)	1.647	0.323-8.388

Table 2s: Instrumental activities of daily living and use of assistive mobility devices before Covid-19 pandemic confinement. Changes observed after nationwide Covid-19 lockdown. 1: Mann–Whitney U test versus Barthel index score. 2: Analysis of the differences between performing the activity of leaving home and the Barthel index score. 3: OR: Odds Ratio. 4: Kruskal-Wallis test versus Barthel index score. 5: NS: not significant. 6: chi-square test before confinement - after confinement. 7: chi-square analysis between patients who left home alone and/or accompanied and those who did not. 8: Chi-square analysis between patients using these mobility assistance devices before and after confinement.

**INSTRUMENTAL ACTIVITIES OF DAILY LIVING, SOCIO-HEALTH DETERMINANTS AND USE OF ASSISTIVE MOBILITY DEVICES**			
**BEFORE CONFINEMENT**			
Leaves home	**YES**	**NO**	**STATISTICAL**
Leaves home – Barthel index score[2]	82 (64.57%)	45 (35.43%)	**z=-2.092; p=0.036[1]**
Leaving home - level of dependence:<br>Moderate dependence<br>Severe dependency	 64 (50.4%)<br>18 (14.3%)	 25 (19.7%)<br>20 (1.6%)	 **OR[3] 0.352**<br>**CI 0.160-0.772**
Leaving home - level of income:<br>Less than 11,200 euros/year.<br>More than 11,200 euros/year	 36 (28.3%)<br>46 (36.2%)	 24 (19%)<br>21 (16.5%)	 OR 0.685<br>CI 0.330-1.421
Leaving home - chronic disease burden:<br>More than 5 chronic diseases<br>Less than 5 chronic diseases	 22 (17.3%)<br>60 (47.3%)	 12 (9.4%)<br>33 (26%)	 OR 0.992<br>CI 0.436-2.256
Leaving home - polypharmacy:<br>More than 5 drugs<br>Less than 5 drugs	 73 (58%)<br>8 (6.3%)	 42 (33.3%)<br>3 (2.4%)	 OR 0.652<br>CI 0.164-2.591
How you get out of the house:<br>- Alone<br>- Accompanied<br>- Does not come out	 16 (12.60%)<br>68 (53.54%)<br>-	 -<br>-<br>43 (33.86%)	 **z=-1.992; p=0.046[4]**
Leaves home for nonmandatory activities	72 (59.50%)	49 (40.50%)	z=-1.841; p= NS[1,4]
Buy autonomously	19 (15.45%)	104 (84.55%)	z=-1.414; p= NS[1,5]
Supervised purchase	17 (13.93%)	105 (86.07%)	z=-0.087; p= NS[1,5]
Wheelchair	41 (32.28%)	86 (67.72%)	z=-1.679; p= NS[1,5]
Walker	45 (35.43%)	82 (64.57%)	z=-0.580; p= NS[1,5]
Crutches-cane	54 (42.52%)	73 (57.48%)	**z=-3.168; p=0.002[1,5]**
**AFTER CONFINEMENT (June 2020)**			
Leaves home	**Yes**	**NO**	**STATISTICAL**
Leaves home – Barthel index score	66 (51.97%)	61 (48.03%)	**χ^2 = 4.144; p<0.05[6]**
Leaving home - level of dependence:<br>Moderate dependence<br>Severe dependency	 53 (41.7%)<br>13 (10.3%)	 36 (28.3%)<br>25 (19.7%)	 **OR 0.353**<br>**CI 0.160-0.780**
Leaving home - level of income:<br>Less than 11,200 euros/year.<br>More than 11,200 euros/year	 23 (18.1%)<br>43 (33.8%)	 37 (29.1%)<br>24 (19%)	 **OR 0.347**<br>**CI 0.169-0.714**
Leaving home - chronic disease burden:<br>More than 5 chronic diseases<br>Less than 5 chronic diseases	 18 (14.3%)<br>48 (37.8%)	 16 (12.5%)<br>45 (35.4%)	 OR 0.948<br>CI 0.432-2.082
Leaving home - polypharmacy:<br>More than 5 drugs<br>Less than 5 drugs	 62 (49.2%)<br>3 (2.4%)	 53 (42.1%)<br>8 (6.3%)	 OR 3.119<br>CI 0.787-12.358
How you get out of the house:<br>- Alone<br>- Accompanied<br>- Does not come out	 10 (7.87%)<br>57 (44.88%)<br>-	 -<br>-<br>60 (47.24%)	 **χ^2 = 4.72; p<0.05[7]**
Leaves home for nonmandatory activities	48 (39.67%)	73 (60.33%)	**χ^2 = 9.52; p<0.001[7]**
Buy autonomously	12 (9.92%)	109 (90.08%)	χ^2 = 1.68; p= NS[5,6]
Supervised purchase	14 (11.57%)	107 (88.43%)	χ^2 = 0.31; p= NS[5,6]
Wheelchair	40(31.50%)	87 (68.50%)	χ^2 = 0.02; p= NS[8]
Walker	38 (29.92%)	89 (70.08%)	χ^2 =0.88; p= NS[8]
Crutches-cane	49 (38.58%)	78 (61.42%)	χ^2 = 0.41; p= NS[8]

Table 3s: Survival at three years of follow-up. Post-hoc tests performed using linear regression analysis in relation to the survival in 2023, June. Stepwise regression analysis. a: Predictors: Independent after confinement, improves functional dependence during covid-19 pandemic confinement to become functionally independent. b: Predictors: Independent after confinement, Sex. c: Predictors: Independent after confinement, Sex, Income level. d: Dependent variable: survival in 2023, June.

POST-HOC REGRESSION ANALYSIS. ORCASITAS COHORT

Model Summary

Model	R	R square	Adjusted R square	Std. Error of the Estimate	Change Statistics				
					Change in R square	Change in F	gl1	gl2	Sig. Change in F
1	0.275[a]	0.076	0.068	0.476	0.076	10.253	1	125	0.002
2	0.363[b]	0.131	0.117	0.464	0.056	7.950	1	124	0.006
3	0.419[c]	0.176	0.156	0.454	0.044	6.628	1	123	0.011

ANOVA[d]

Model	Sum of squares	gl	Mean Square	F	Sig.
1					
Regression	2.328	1	2.328	10.253	0.002[a]
Residual	28.381	125	0.227		
Total	30.709	126			
2					
Regression	4.038	2	2.019	9.386	0.000[b]
Residual	26.671	124	0.215		
Total	30.709	126			
3					
Regression	5.401	3	1.800	8.751	0.000[c]
Residual	25.307	123	0.206		
Total	30.709	126			

Coefficients[d]

Model	Unstandardized Coefficients		Standardized Coefficients	t	Sig.	95% Confidence interval for B	
	B	Std. Error	Beta			Lower Bound	Upper Bound
1[a]							
(Constant):	1.801	0.129		13.919	0.000	1.545	2.057
Independent after confinement	-0.273	0.085	-0.275	-3.202	0.002	-0.442	-0.104
2[b]							
(Constant):	1.461	0.174		8.381	0.000	1.116	1.806
Independent after confinement	-0.276	0.083	-0.278	-3.323	0.001	-0.440	-0.112
Sex	0.284	0.101	0.236	2.820	0.006	0.085	0.483
3[c]							
(Constant):	1.754	0.205		8.556	0.000	1.348	2.160
Independent after confinement	-0.256	0.082	-0.258	-3.137	0.002	-0.418	-0.095
Sex	0.281	0.098	0.234	2.855	0.005	0.086	0.476
Income level	0.209	0.081	-0.212	-2.575	0.011	-0.369	-0.048

Table 4s: Survival at three years of follow-up. Post-hoc tests performed using linear regression analysis in relation to the survival in 2023, June. Stepwise regression analysis. a: Predictors: Sex. b: Predictors: Sex, Barthel level. c: Predictors: Sex, Barthel level, Leaves home. d: Predictors: Sex, Barthel level, Leaves home, Chair-bed transfer. e: Dependent variable: survival in 2023, June. f: Leaves home post-confinement. g: Chaid-bed transfer post-confinement.

POST-HOC REGRESSION ANALYSIS. ORCASITAS COHORT

Model Summary

Model	R	R square	Adjusted R square	Std. Error of the Estimate	Change Statistics				
					Change in R square	Change in F	gl1	gl2	Sig. Change in F
1	0.233[a]	0.054	0.047	0.482	0.054	7.154	1	125	0.008
2	0.405[b]	0.164	0.150	0.455	0.110	16.235	1	124	0.000
3	0.463[c]	0.214	0.195	0.443	0.051	7.914	1	123	0.006
4	0.498[d]	0.248	0.223	0.435	0.034	5.442	1	122	0.021

ANOVA[e]

Model	Sum of squares	gl	Mean Square	F	Sig.
1					
Regression	1.662	1	1.662	7.154	0.008[a]
Residual	29.046	125	0.232		
Total	30.709	126			
2					
Regression	5.025	2	2.513	12.130	0.000[b]
Residual	25.684	124	0.207		
Total	30.709	126			
3					
Regression	6.578	3	2.193	11.176	0.000[c]
Residual	24.131	123	0.196		
Total	30.709	126			
4					
Regression	7.608	4	1.902	10.045	0.000[d]
Residual	23.100	122	0.189		
Total	30.709	126			

Coefficients[e]

Model	Unstandardized Coefficients		Standardized Coefficients	t	Sig.	95% Confidence interval for B	
	B	Std. Error	Beta			Lower Bound	Upper Bound
1							
(Constant):	1.070	0.134		8.000	0.000	0.806	1.335
Sex	0.280	0.105	0.233	2.675	0.008	0.073	0.487
2							
(Constant):	1.673	0.196		8.545	0.000	1.286	2.061
Sex	0.281	0.099	0.234	2.846	0.005	0.086	0.476
Barthel level	-0.355	0.088	-0.331	-4.029	0.000	-0.530	-0.181
3							
(Constant):	1.275	0.237		5.370	0.000	0.805	1.745
Sex	0.248	0.097	0.206	2.563	0.012	0.056	0.440
Barthel level	-0.297	0.088	-0.277	-3.366	0.001	-0.472	-0.122
Leaves home[f]	0.229	0.081	0.233	2.813	0.006	0.068	0.390
4							
(Constant)	1.559	0.263		5.924	0.000	1.038	2.080
Sex	0.249	0.095	0.207	2.619	0.010	0.061	0.437
Barthel level	-0.216	0.093	-0.201	-2.308	0.023	-0.401	-0.031
Leaves home[f]	0.169	0.084	0.172	2.014	0.046	0.003	0.336
Chair-bed transfer[g]	-0.212	0.091	-0.212	-2.333	0.021	-0.392	-0.032
