# Supplementary material for: Socio-health factors, ability to perform instrumental and basic activities of daily living, and use of assistive mobility devices during the COVID-19 pandemic: Interrelationships and impact on long-term survival
Source: PLoS One. 2025 May 19;20(5):e0318481. doi: 10.1371/journal.pone.0318481 (PMC12088529; doi:10.1371/journal.pone.0318481)
Supplement: S3 Text — (PDF) [file pone.0318481.s003.pdf]

Supplement 3: “Basic data of the study”

Socio-health factors, ability to perform instrumental and basic activities of daily living and use of assistive mobility devices during the Covid-19 pandemic. Interrelationships and impact on long-term survival.

Authors:

Vicente Martín Moreno. María Inmaculada Martínez Sanz. Irene Sánchez González. Miguel Recuero Vázquez. Sara Guerra Maroto. Miriam Fernández Gallardo. Amanda Martín Fernández. Julia Herranz Hernando. María Palma Benítez Calderón. Eva Sevillano Fuentes. Elena Pérez Rico. Laura Calderón Jiménez. Elena Sánchez Rodríguez. Helena Alonso Samperiz. Irene León Saiz. On behalf of GIDO collaborative group (Orcasitas Dependency Research Group). Juana Marcos Guerra.

Address for correspondence:

Vicente Martín Moreno

e-mail: amanvic@hotmail.com

Anexo 1:

Minimum data set to validate the results represented in the study performed. Part of the data is contained in the manuscript itself. The data in this appendix allow validation of the data provided through means, percentages and other descriptive analyses.

Data Module 1

Marital status[1]	Education level[2]	Income level[3]	Barthel level BC[4]	Barthel level AC[5]	Barthel score BC[6]	Barthel score AC[7]
2	3	1	1	1	30	20
2	3	2	2	1	50	20
2	3	1	2	3	50	75
1	4	1	2	2	45	45
2	3	2	2	3	60	75
2	1	2	2	3	40	75
2	3	1	2	2	60	50
1	3	2	1	1	15	15
2	8	1	1	2	35	60
2	2	2	2	2	55	45
2	2	1	2	2	40	55
2	3	1	1	1	5	15
2	2	2	2	2	50	50
2	2	1	1	1	5	0
1	3	2	1	3	10	80
1	1	1	2	3	60	85
2	6	2	2	1	55	15
1	3	2	2	1	55	35
2	2	1	2	3	60	65
2	2	2	2	3	60	70
1	5	2	2	1	40	30
2	3	1	2	3	60	85
2	3	1	1	1	25	20
2	3	2	2	2	60	60
2	3	2	2	2	60	60
2	3	2	2	3	60	65
2	3	1	2	3	50	65
3	2	1	2	2	50	50
1	2	2	2	1	50	25
4	3	2	2	2	60	50
2	3	1	1	1	5	0
2	3	1	2	1	60	30
2	3	2	2	1	60	25
2	3	1	2	2	50	60
2	2	1	2	3	60	75
1	3	1	2	2	45	50
2	1	1	1	2	25	55
2	3	1	1	2	25	50
2	1	1	1	1	30	30
1	2	1	1	1	5	25
2	2	1	1	2	15	40
1	3	1	1	2	5	55
2		2	1	1	30	20
2	4	2	1	1	25	35
2	3	1	2	2	45	60
2	3	2	2	2	50	50
1	3	1	1	2	30	60
1	3	2	2	3	55	65

3	3	1	2	2	60	60
2	3	1	1	1	10	35
2	3	2	2	3	60	90
2	3	2	1	1	5	0
2	3	1	2	3	60	80
2	3	2	2	2	50	40
2	4	1	1	1	20	5
2	3	1	2	3	40	95
2	3	2	2	2	55	50
2	3	1	2	2	60	45
4	3	1	2	1	50	20
1	3	2	2	3	45	75
1	1	2	2	3	60	65
2	1	2	1	2	2	60
1	7	1	2	3	50	75
2	3	2	1	1	0	0
2	3	2	1	1	15	15
1	3	2	2	3	60	75
1	3	2	2	3	45	90
1	3	1	1	1	35	10
2	2	1	2	3	55	80
1	6	2	2	1	50	30
2	1	2	2	3	60	95
1	3	1	2	3	50	80
1	2	2	2	3	60	85
2	3	1	2	3	50	65
2	1	2	2	2	60	60
2	3	1	2	3	45	75
2	1	1	2	3	60	75
2	1	2	2	3	40	85
2	3	1	2	2	60	60
2	6	1	2	3	60	70
2	2	1	2	3	55	80
2	4	1	1	2	0	40
2	3	1	1	3	30	70
2	1	1	2	3	40	80
2	2	2	1	3	9	85
2	3	2	2	3	55	70
1	5	2	1	2	35	40
1	3	2	2	3	60	70
1	3	2	2	2	45	40
1	3	1	1	1	20	0
3	4	1	1	1	0	5
2	3	2	2	3	50	75
2	3	2	2	1	60	30
1	3	2	1	3	30	95
1	4	1	1	2	30	40
2	3	2	2	3	55	75
2	4	1	2	3	60	80
2	3	1	2	3	60	80
2	1	2	2	3	60	65
2	3	1	1	1	0	0

1	3	2	2	1	40	30
1	4	1	2	3	60	80
2	3	2	2	2	60	55
1	2	2	2	2	40	60
1	3	1	2	2	55	45
2	2	2	2	3	60	80
2	2	2	1	2	20	55
2	2	1	1	2	35	60
2	2	2	2	3	55	75
1	2	2	2	2	60	50
1	1	2	2	3	60	75
2	3	2	2	3	60	95
2	2	2	2	3	60	90
2	2	2	2	3	60	85
1	3	1	2	2	40	50
1	2	2	2	3	60	85
1	3	2	2	3	60	85
4	2	2	2	3	55	65
2	2	2	2	3	60	75
1	2	2	2	2	55	50
1	2	2	1	1	30	25
2	2	1	1	1	5	5
2	1	1	2	3	55	90
2	1	2	2	3	60	95
2	2	2	1	1	15	25
2	2	1	2	3	60	65
1	2	2	2	2	55	50

1: Marital status: 1: married. 2: widowed. 3: separated/divorced. 4: single.

2: Education level: 1: Illiterate. 2: Uneducated but can read and write. 3: Incomplete primary education. 4: Primary education. Includes elementary baccalaureate degree. 5: Professional training certification. 6: Middle education (high school). 7: Industrial master's degree. 8: Higher education. For practical purposes. in the study conducted. items 1. 2 and 3 were grouped together as “Insufficient education”. Similarly. items 4 and 5 were grouped as “Primary education”. since in both cases the training period ended at the same age. Finally. items 6 and 7 were grouped as “Middle education”. Also, because they are training cycles that take place at the same age.

3: Income level: 1: economic income less than 11.200 euros/year. 2: income over 11.200 euros/year.

4: Barthel level BC: level of functional ADL-dependence using the Barthel index before confinement due to the Covid-19 pandemic. 1: severe dependency. 2: moderate dependence.

5: Barthel level AC: level of functional dependence using the Barthel index after confinement for the Covid-19 pandemic. 1: severe dependence. 2: moderate dependence. 3: Barthel after confinement greater than 60. no longer functionally dependent.

6: Barthel score BC: Barthel index score before confinement.

7: Barthel score BC: Barthel index score after confinement.

Data Module 2

Community dwelling BC[1]	Community dwelling AC[2]	Crutches or cane BC[3]	Walker BC[4]	Wheelchair BC[5]	Crutches or cane AC[6]	Walker AC[7]	Wheelchair AC[8]
1	1	2	1	1	2	2	1
1	1	1	2	1	1	2	1
2	2	2	1	2	2	1	2
2	2	2	2	2	2	2	2
2	2	1	2	2	1	2	2
1	1	1	2	2	1	2	2
1	2	1	2	2	2	1	2
2	2	2	2	2	2	2	2
2	2	1	2	2	1	2	2
2	2	2	2	2	2	2	2
1	1	2	2	2	2	2	2
2	2	2	2	1	2	2	1
1	1	2	1	2	2	1	2
2	2	2	2	1	2	2	1
1	1	1	2	2	1	2	2
1	1	1	2	2	1	2	2
1	2	1	1	1	2	1	1
1	2	2	2	2	2	2	2
1	2	1	1	1	1	1	1
1	2	1	2	2	1	2	2
2	2	1	2	2	1	2	2
2	2	2	2	2	2	2	2
2	2	2	1	2	2	2	2
1	1	2	1	2	2	1	2
1	2	2	1	1	2	1	2
2	2	1	2	2	1	2	2
1	1	2	1	2	2	1	2
1	1	1	2	2	1	2	2
1	1	1	2	2	1	2	2
2	2	2	1	1	2	1	1
2	2	2	2	2	2	2	2
1	1	2	1	1	2	1	1
1	1	2	2	1	2	2	1
1	1	2	2	2	2	2	2
2	2	1	2	1	1	2	1
1	1	1	2	2	1	2	2
2	2	2	1	2	2	1	2
2	2	2	1	2	2	1	2
2	2	2	1	2	2	1	2
2	2	2	2	1	2	2	1
2	2	2	2	2	2	2	2
1	1	2	1	1	1	2	1
1	1	2	2	1	2	2	1
1	1	2	1	2	1	1	1
1	1	1	2	2	1	2	2
2	1	1	1	2	2	1	1
1	2	1	1	1	2	2	2

1	1	2	2	2	2	2	2
1	1	1	1	2	1	2	2
2	2	2	2	1	2	2	1
1	2	2	1	2	2	2	2
2	2	2	2	2	2	2	1
1	1	1	2	1	1	2	2
1	1	2	1	1	2	2	1
1	2	2	1	1	2	1	2
2	2	2	2	2	2	2	2
1	1	1	2	2	1	2	2
1	1	1	1	2	1	1	2
2	2	2	1	2	2	2	2
1	1	1	2	2	1	2	2
2	2	2	1	2	2	1	2
1	1	2	1	1	2	1	1
1	2	1	2	2	1	2	2
1	1	2	2	1	2	2	1
1	1	2	2	2	2	2	1
1	1	2	2	2	2	2	2
1	1	1	1	2	2	1	2
2	2	2	2	1	2	2	2
1	1	1	2	2	2	2	2
2	2	2	1	2	2	1	2
1	1	1	2	2	1	2	2
1	2	2	2	2	2	2	2
2	2	1	1	2	1	1	2
1	1	1	2	1	1	2	1
2	1	2	1	1	2	1	1
1	1	2	1	1	2	1	1
1	1	1	2	2	1	2	2
2	1	2	1	2	2	1	2
1	2	1	2	2	1	2	2
1	1	2	2	2	2	2	2
1	2	1	2	2	1	2	2
1	2	2	1	1	2	1	1
2	2	2	2	1	2	2	1
1	1	1	1	2	1	1	2
1	1	1	2	2	1	2	2
1	2	2	1	2	2	1	2
2	1	2	2	2	2	1	1
1	1	2	1	2	1	2	2
1	1	1	2	1	1	2	1
2	2	2	2	1	2	2	1
1	2	2	2	1	2	2	1
1	1	1	2	2	1	2	1
1	1	2	2	1	2	2	1
1	1	2	2	2	2	2	2
2	2	1	2	2	1	2	2
1	1	1	2	2	1	2	2
1	2	1	2	1	1	2	2
2	2	2	1	1	2	1	1
1	1	1	2	2	1	2	2

2	2	2	2	2	2	2	2
1	1	2	2	1	2	2	1
1	1	2	2	2	2	2	2
2	2	2	2	1	2	2	1
2	2	2	2	1	2	2	1
1	2	2	2	1	2	2	1
2	2	2	1	1	2	1	2
1	1	1	1	1	1	1	1
1	1	2	2	2	2	2	2
1	1	1	2	2	1	2	2
1	2	1	2	2	1	2	2
1	1	1	2	2	1	2	2
1	1	2	2	2	2	2	2
1	1	1	1	2	1	1	2
1	1	1	2	2	1	2	2
1	1	1	1	2	1	1	2
1	1	1	1	1	1	1	1
1	1	2	1	2	2	1	2
2	2	1	2	2	1	2	2
1	1	1	1	2	1	1	2
2	2	1	2	2	2	2	2
2	2	1	2	2	2	2	2
1	2	2	2	1	2	2	1
1	1	2	2	2	2	2	2
1	1	2	2	2	2	2	2
1	2	2	1	2	2	2	2
2	2	1	2	2	1	2	2
2	1	2	2	2	2	1	2

1: Community dwelling BC: Community dwelling mode before confinement. 1: leaves home. 2: lives homebound.

2: Community dwelling AC: Community dwelling mode after confinement. 1: leaves home. 2: lives homebound.

3: Crutches-cane BC: Crutches-cane before confinement. 1: uses crutches-cane. 2: does not use crutches-cane.

4: Walker BC: Walker before confinement. 1: uses walker. 2: does not use walker.

5: Wheelchair BC: Wheelchair before confinement. 1: uses wheelchair. 2: does not use wheelchair.

6: Crutches-cane AC: Crutches-cane after confinement. 1: uses crutches-cane. 2: does not use crutches-cane.

7: Walker AC: Walker after confinement. 1: uses walker. 2: does not use walker.

8: Wheelchair AC: Wheelchair after confinement. 1: uses wheelchair. 2: does not use wheelchair.

Data Module 3

Public assistant[1]	Private assistant[2]	Internal caregiver[3]	Public assistant (hours)[4]	Diseases burden[5]	Diseases[6] (n)	Polimedicated[7]
2	1	1	0	1	3	1
2	1	1	0	2	6	1
1	2	2	2	1	3	1
1	2	2	9	2	5	1
2	2	2	0	1	2	1
1	2	2	0	1	2	1
2	2	2	0	1	4	1
1	1	1	8	1	3	1
2	2	2	0	1	2	1
2	1	1	0	1	3	1
2	2	2	0	1	1	1
1	2	2	1	2	5	1
2	1	1	0	1	2	1
1	2	2	3	1	3	1
1	2	2	1	1	2	1
2	2	2	0	1	4	1
2	2	2	0	1	2	1
1	2	2	1	1	3	1
1	2	2	1	1	3	1
1	2	2	1	1	4	1
1	1	1	8	1	4	1
2	2	2	0	1	4	1
2	1	2	0	2	6	1
2	1	1	0	2	5	1
1	1	2	1	1	3	1
1	2	2	2	1	3	1
1	2	2	1	2	5	1
1	2	2	2	1	0	1
1	1	1	1	1	2	1
1	2	2	1	1	4	1
2	1	1	0	1	3	1
1	2	2	2	2	5	1
1	2	2	3	2	5	1
2	1	1	0	1	4	1
2	2	2	0	1	2	1
1	2	2	0	1	4	1
1	2	2	3	2	8	1
2	2	2	0	2	6	1
1	2	2	3	1	1	2
1	1	2	4	2	5	1
1	1	2	2	1	4	1
2	2	2	0	1	3	1
1	2	2	2	2	6	1
1	1	2	4	1	2	2
1	2	2	4	1	3	1
1	2	2	2	1	1	1
1	2	2	1	2	6	2

1	2	2	2	1	3	2
1	1	2	1	2	5	1
1	2	2	1	1	2	1
1	2	2	1	2	6	1
1	2	2	2	1	1	1
2	2	1	0	2	5	1
2	1	2	0	1	4	1
1	2	2	3	1	2	1
2	2	2	0	1	2	1
1	2	2	2	1	4	1
1	1	2	1	1	2	1
2	2	2	0	1	3	1
1	1	2	2	1	3	1
2	2	2	1	1	1	1
1	1	1	2	1	4	1
1	1	2	1	1	4	1
1	1	1	1	1	3	1
2	1	2	0	1	2	2
1	2	2	2	2	7	1
1	2	2	1	1	3	1
2	2	2	0	2	5	1
2	2	2	0	2	7	1
2	2	2	0	2	5	1
2	2	2	0	1	3	1
2	2	2	0	1	4	1
2	2	2	0	2	5	1
2	1	1	0	1	3	1
2	1	1	0	1	2	1
2	2	2	0	2	5	1
2	2	2	0	1	3	1
2	1	2	0	1	3	1
2	1	2	0	1	3	1
1	2	2	1	1	4	1
2	2	2	0	1	4	2
1	1	1	3	1	1	2
1	2	2	0	2	8	1
2	1	1	0	2	5	1
1	2	2	1	1	2	1
1	2	2	1	2	6	1
2	2	2	0	2	6	1
1	1	2	0	1	4	1
1	1	2	1	2	5	1
1	2	2	3	1	3	1
2	1	1	0	1	4	1
2	1	1	0	2	6	1
1	2	2	2	1	3	1
2	2	2	0	1	2	1
2	2	2	1	1	3	2
1	2	2	1	1	4	1
1	2	2	1	2	5	1
1	1	1	2	1	1	1
1	1	2	1	1	3	1

2	1	2	0	1	3	1
2	1	1	0	1	2	1
1	2	2	1	1	2	1
2	2	2	0	1	4	1
2	2	2	0	1	4	1
2	2	2	0	2	5	1
1		1	1	2	6	1
1	1	1	1	2	6	1
2	1	2	0	1	2	1
1	2	2	1	1	4	1
2	2	2	2	1	4	1
1		2	3	2	6	1
2	1		0	1	3	
			1	1	1	1
2	2	2	0	1	4	1
1	2	2	1	1	2	1
2	2	2	0	1	4	1
1	2	2	1	1	4	1
1	1	2	2	1	2	1
1	2	2	1	2	8	1
2	1	2	0	1	2	1
2	1	2	0	1	2	1
1	1	1	0	1	3	2
1	2	2	1	1	2	1
1	2	2	1	1	2	1
2	2	2	0	1	3	2
1	2	2	1	1	2	2
1	1	2	0	1	4	1

1: Public assistant: 1: Yes. 2: No

2: Private assistant: 1: Yes. 2: No.

3: Internal caregiver. 1: Yes. 2: No.

4: Public assistant (hours): number of hours per week of provision of housework and personal care services. 0: not assistant. 1: 1-5 hours per week: 2: 6-10 hours per week. 3: 11-15 hours per week. 4: 16-20 hours per week. 5: 21-25 hours per week. 6: 26-30 hours per week. 7: 31-35 hours per week. 8: 36-39 hours per week. 9: >40 hours per week.

5: Disease burden: 1: 5 or more chronic diseases. 2: less than 5 chronic diseases. In both cases. chronic diseases are included in the Strategy of Care for People with Chronic Diseases (EAPEC).

6: Diseases: number of chronic diseases listed in the EAPEC that the individual has.

7: Polymedicated: 1: polymedicated person (prescribed 5 or more active ingredients). 2: person not polymedicated.

Data Module 4

Diabetes mellitus	COPD	Stroke	Hypertension	Ischemic cardiopathy
2	2	2	1	2
1	2	2	1	1
2	2	2	2	1
1	1	2	1	2
2	1	2	1	2
1	2	2	1	2
1	1	2	1	2
1	2	2	1	2
2	2	2	1	2
1	2	1	1	2
2	2	2	2	2
1	2	1	1	1
1	2	2	2	2
1	2	2	2	2
2	2	2	2	2
1	1	2	1	2
2	2	2	1	2
2	2	2	1	1
2	1	2	1	2
2	1	2	1	1
1	2	1	1	2
2	2	2	1	2
2	2	2	1	1
2	1	2	1	2
1	2	2	1	2
2	2	1	2	1
2	2	2	1	1
2	2	2	2	2
1	2	2	2	2
1	2	2	1	2
2	2	1	1	2
1	2	2	1	1
1	2	2	1	1
1	2	2	1	2
2	2	2	1	2
1	2	2	1	2
1	2	1	1	1
1	1	2	1	2
2	2	2	2	2
1	2	1	1	1
2	2	1	1	1
1	2	1	1	2
1	2	2	1	1
2	2	2	1	2
2	1	2	1	2
2	2	2	1	2
1	1	2	1	2
1	2	2	2	2

2	2	1	1	1
1	2	2	1	2
1	2	1	1	1
2	2	2	1	2
1	2	1	1	2
2	2	2	1	2
2	1	2	2	2
2	2	2	1	2
1	2	2	1	2
2	2	2	1	1
2	2	2	1	2
2	1	2	1	2
2	2	2	2	2
1	2	1	1	2
2	1	2	1	2
2	2	2	1	2
2	2	2	1	2
1	2	1	1	2
2	2	2	1	2
1	2	2	1	2
2	1	1	1	1
1	2	2	1	2
1	2	2	1	2
2	2	2	1	2
1	2	2	1	1
2	2	2	1	2
2	2	2	1	2
1	1	2	1	1
1	2	2	1	2
1	2	2	2	2
2	2	2	1	2
1	2	1	1	2
2	2	2	1	2
2	2	2	2	2
1	2	1	1	1
2	2	2	1	1
2	2	2	1	2
1	2	1	1	1
1	2	2	1	1
1	2	2	1	2
1	2	1	1	2
1	2	1	1	2
2	2	1	1	2
2	2	2	1	1
2	2	2	1	2
2	2	2	1	2
2	2	2	1	2
2	2	2	1	2
2	1	2	2	1
2	2	2	1	2
2	2	2	1	2
1	2	2	1	2

2	2	2	1	2
1	2	2	2	2
2	2	2	1	2
1	2	2	1	1
2	1	2	1	1
1	1	2	1	2
1	2	1	1	2
2	2	2	1	2
2	2	2	1	2
2	2	2	1	1
1	2	2	1	1
2	2	1	1	2
2	2	2	2	2
2	2	2	1	2
2	2	2	1	2
1	1	2	1	2
2	2	1	1	2
2	2	2	1	2
1	1	1	1	1
2	2	2	1	2
1	2	2	2	1
2	2	2	1	2
2	2	2	1	1
2	2	2	1	2
1	2	2	1	2
2	2	2	1	2
1	2	2	1	2

Chronic diseases included in the Strategy for the Care of People with Chronic Diseases (EAPEC) of the Madrid Health Service. In all variables: 1: has the disease. 2: does not have the disease. COPD: chronic obstructive pulmonary disease.

Data Module 5

Heart failure	Asthma	Obesity	Dyslipidemia	Kidney failure
2	2	1	1	2
2	2	1	1	1
1	2	2	1	2
1	2	1	2	2
2	2	2	2	2
2	2	2	2	2
2	2	2	2	1
2	2	2	1	2
2	2	1	2	2
2	2	2	2	2
2	2	2	1	2
2	2	2	1	2
2	2	2	1	2
2	2	1	1	2
2	2	1	1	2
2	2	2	1	2
2	2	1	2	2
2	2	1	2	2
2	2	1	2	2
2	2	2	1	2
2	2	1	2	2
1	2	1	1	2
1	1	1	1	2
1	2	1	2	1
2	2	1	2	2
1	2	2	2	2
1	2	1	1	2
2	2	2	2	2
2	2	2	1	2
2	2	1	1	2
2	2	2	1	2
1	2	1	2	2
1	2	2	1	2
2	2	1	2	1
1	2	2	2	2
2	2	1	1	2
1	2	1	1	1
1	2	1	2	1
2	2	1	2	2
2	2	2	2	1
2	2	2	2	1
2	2	2	2	2
1	2	1	1	2
2	2	1	2	2
2	2	1	2	2
2	2	2	2	2
1	2	1	2	1

2	2	2	1	1
1	2	2	1	2
2	2	2	2	2
2	2	1	1	2
2	2	2	2	2
2	2	1	1	2
2	2	1	1	1
2	1	2	2	2
2	2	2	1	2
2	2	1	1	2
2	2	2	2	2
2	2	1	1	2
2	2	2	1	2
2	2	2	1	2
2	2	1	2	2
1	2	1	2	2
2	2	2	1	1
2	2	2	1	2
1	2	1	1	1
2	2	1	1	2
2	2	1	1	1
2	1	2	1	1
1	2	1	1	2
2	2	2	1	2
1	2	1	1	2
2	2	1	1	2
1	2	2	2	1
2	2	1	2	2
2	2	2	1	2
2	2	2	1	2
2	2	2	1	1
2	2	1	1	2
2	2	2	1	2
2	1	2	1	1
2	2	2	1	2
1	2	1	1	1
1	2	1	1	2
2	1	2	2	2
2	2	1	1	2
1	2	2	1	1
2	2	1	1	2
2	2	2	1	1
2	2	2	2	2
2	1	2	1	2
1	2	1	1	2
2	2	1	1	2
2	2	2	2	1
2	2	2	1	1
2	2	1	1	1
1	2	1	1	2
2	2	2	2	2
2	2	1	1	2

2	2	2	1	2
2	2	2	1	2
2	2	2	1	2
2	2	1	1	1
2	2	1	2	2
1	2	2	1	2
1	2	1	1	2
1	1	2	1	2
2	2	2	1	2
2	1	1	1	2
2	2	1	1	2
1	2	1	1	2
2	2	2	1	2
2	1	2	2	2
2	2	1	1	1
2	2	2	1	2
2	2	1	2	2
2	2	1	1	2
1	2	2	2	2
1	2	1	1	2
2	2	1	2	2
2	2	2	2	2
1	2	1	2	2
2	2	2	2	2
2	2	2	1	2
2	2	1	2	2
2	2	2	1	2
1	2	1	2	2

Chronic diseases included in the Strategy for the Care of People with Chronic Diseases (EAPEC) of the Madrid Health Service. In all variables: 1: has the disease. 2: does not have the disease.

Data Module 6

Survival 6 months	Survival 12 months	Survival 18 months	Survival 24 months	Survival 30 months	Survival 36 months
1	1	1	1	2	2
1	1	1	1	1	1
1	1	1	1	1	1
2	2	2	2	2	2
1	1	1	1	1	2
1	1	1	1	1	1
1	2	2	2	2	2
1	1	1	1	1	1
1	1	1	1	1	1
1	1	1	1	1	2
1	2	2	2	2	2
1	2	2	2	2	2
1	1	1	1	1	1
1	1	1	1	1	1
1	1	1	2	2	2
1	1	1	1	1	1
1	1	2	2	2	2
1	1	1	1	1	1
1	1	2	2	2	2
2	2	2	2	2	1
1	1	1	1	1	1
1	1	1	1	1	1
1	1	2	2	2	2
1	1	1	1	1	1
1	1	1	1	1	1
1	1	1	1	1	1
1	1	1	1	1	1
1	1	1	1	1	1
1	1	1	1	2	2
1	1	1	1	1	1
1	1	2	2	2	2
1	1	1	1	1	1
1	2	2	2	2	2
1	1	1	1	1	1
1	1	1	1	1	1
1	1	1	1	1	1
1	2	2	2	2	2
1	1	1	1	2	2
1	1	1	1	1	1
1	1	1	2	2	2
1	1	2	2	2	2
1	2	2	2	2	2
1	1	1	1	1	1
1	1	1	1	1	1
1	1	1	1	1	1
1	1	1	1	1	1
1	2	2	2	2	2
1	1	1	1	1	1

1	1	1	1	1	1
1	2	2	2	2	2
1	1	1	1	1	1
1	2	2	2	2	2
1	1	1	1	1	1
1	1	1	1	1	2
1	1	2	2	2	2
1	2	2	2	2	2
1	1	1	1	1	1
1	2	2	2	2	2
1	1	1	1	1	1
1	1	1	1	1	1
1	1	1	1	1	1
1	1	1	1	1	1
1	2	2	2	2	2
1	1	2	2	2	2
1	2	2	2	2	2
1	1	1	1	1	1
1	1	1	1	1	1
2	2	2	2	2	2
1	1	1	1	1	1
1	2	2	2	2	2
1	1	1	1	1	1
1	1	1	1	1	1
1	1	1	1	1	1
2	2	2	2	2	2
1	1	1	1	1	1
1	1	1	1	1	1
1	1	2	2	2	2
1	1	2	2	2	2
1	1	2	2	2	2
1	1	1	1	1	1
1	1	1	1	1	1
1	2	2	2	2	2
1	1	1	1	1	1
1	1	1	1	1	1
1	1	1	1	1	1
1	1	1	1	1	1
2	2	2	2	2	2
1	1	1	1	1	1
1	1	1	1	1	1
1	2	2	2	2	2
1	1	2	2	2	2
1	1	1	2	2	2
1	1	1	2	2	2
1	1	1	1	1	1
2	2	2	2	2	2
1	1	1	1	1	1
2	2	2	2	2	2
1	1	1	1	1	1
1	1	1	1	1	1
2	2	2	2	2	2

1	1	1	1	1	1
1	1	1	1	1	1
1	1	1	1	1	1
1	1	1	1	2	2
1	1	1	2	2	2
1	1	1	1	2	2
1	1	1	1	1	1
1	1	1	1	1	1
1	1	1	1	1	1
1	1	1	1	1	1
1	1	1	1	1	1
1	1	1	1	1	1
1	1	1	1	1	1
1	1	1	1	1	1
1	1	1	1	1	1
1	1	1	1	1	1
1	1	1	1	1	1
1	2	2	2	2	2
1	1	2	2	2	2
1	1	1	1	1	1
1	1	1	1	1	1
1	1	1	2	2	2
1	1	1	1	1	1
1	1	1	1	1	1
1	1	1	1	1	2
1	2	2	2	2	2
1	1	1	1	1	1

Survival at 6-month time intervals from June 2020 to June 2023. First cut-off December 2020. Subsequent cut-off points June 2021. December 2021. June 2022. December 2022 and June 2023. Interpretation: 1: alive. 2: deceased.

Data Module 7: Level of dependency for each activity of the Barthel index according to the classic score

Feeding[1]	Bathing[2]	Dressing-undressing[3]	Grooming[4]	Fecal incontinence[5]
2	1	1	1	3
2	1	1	1	1
3	2	3	2	3
3	1	1	1	3
3	2	3	2	2
3	2	2	2	3
3	2	1	1	2
1	1	1	1	1
3	1	2	2	3
3	1	2	1	2
3	1	2	1	2
3	1	1	2	1
2	1	2	2	3
1	1	1	1	1
3	1	2	2	3
3	2	2	2	3
1	1	1	1	1
3	1	2	1	1
3	2	2	1	3
3	1	3	2	3
2	1	1	1	2
3	2	3	2	3
2	1	1	1	2
3	1	2	1	3
3	2	3	2	1
3	1	3	2	1
3	2	2	2	3
3	1	2	1	1
3	1	1	1	1
3	1	2	2	3
1	1	1	1	1
3	1	1	1	3
3	1	1	1	1
2	1	2	1	3
3	2	3	1	3
1	1	2	2	3
1	2	3	2	2
3	1	2	2	3
3	1	1	1	2
3	1	1	1	1
2	1	1	1	3
3	1	2	2	2
1	1	1	1	2
2	1	2	1	2
3	1	2	2	2
3	2	2	1	2

3	1	2	1	3
3	1	2	1	3
3	1	3	2	3
2	1	1	1	3
3	2	3	2	3
1	1	1	1	1
3	2	2	1	3
3	1	2	2	1
1	1	1	1	2
3	1	3	2	3
2	2	2	1	3
2	1	2	1	2
2	1	1	1	1
3	1	2	2	3
3	1	3	1	3
3	1	2	1	3
3	2	3	2	3
1	1	1	1	1
1	1	1	1	1
3	1	3	2	3
3	2	3	2	3
1	1	1	1	2
2	2	3	2	3
2	1	1	1	3
3	2	3	2	3
3	2	2	1	3
3	2	3	2	3
3	1	2	2	3
3	1	2	1	3
3	1	3	2	3
3	1	2	2	3
3	2	3	2	3
2	1	2	2	3
3	1	2	2	3
3	2	3	2	3
2	1	1	1	2
3	2	3	2	3
3	1	2	2	3
3	2	3	2	3
3	1	2	2	2
3	1	1	1	2
3	1	2	2	3
3	1	1	2	2
1	1	1	1	1
1	1	1	1	1
2	1	2	2	3
1	1	1	1	2
3	2	3	2	3
3	1	2	1	2
3	2	3	2	2

3	1	3	2	3
3	2	2	2	3
3	1	2	2	3
1	1	1	1	1
1	1	1	1	3
3	1	2	2	3
3	1	2	1	3
3	2	3	1	3
3	1	2	2	3
3	1	2	2	3
3	1	1	1	3
2	1	1	2	3
3	1	2	2	3
2	1	1	1	3
3	1	3	1	3
3	2	3	2	3
3	1	2	2	3
3	2	3	2	2
3	1	2	1	2
3	2	3	2	1
3	2	3	2	3
3	1	2	2	3
3	1	3	2	3
3	1	2	2	1
2	1	1	1	1
2	1	1	1	1
3	2	3	2	3
3	2	3	2	3
3	1	1	1	1
3	1	2	2	2
3	1	2	2	2

1: Feeding: 1: Dependent. 2: Help. 3: Independent.

2: Bathing: 1: Dependent. 2: Independent.

3: Dressing-undressing: 1: Dependent. 2: Help. 3: Independent or minimal help.

4: Grooming: 1: Dependent. 2: Independent.

5: Anal sphincter control: 1: Incontinent. 2: Occasional incontinent. 3: Continent.

Data Module 8: Level of dependency for each activity of the Barthel index according to the classic score

Toilet use[1]	Up- down stairs[2]	Urinary incontinence[3]	Chair-to-bed transfer[4]	Walking on level surfaces[5]
1	1	1	2	1
1	1	1	2	3
3	2	2	2	3
2	1	2	2	3
3	1	3	3	3
3	2	2	3	3
3	1	2	2	3
1	1	1	2	3
3	1	2	2	3
2	2	1	2	3
2	2	2	3	3
1	1	1	1	1
2	2	1	2	3
1	1	1	1	1
3	3	3	3	3
3	3	3	3	3
1	1	1	2	3
1	1	1	3	3
3	1	3	2	3
3	1	2	3	3
1	1	1	3	3
3	2	2	3	4
2	1	1	2	1
2	1	3	3	3
2	1	2	3	3
2	3	2	3	3
3	1	1	3	3
2	3	1	3	3
1	1	1	2	3
2	1	2	2	1
1	1	1	1	1
2	1	1	2	1
1	1	1	2	3
2	3	2	3	3
3	1	3	3	3
3	1	1	3	1
3	1	2	2	3
2	1	1	2	3
1	1	1	2	3
1	1	1	2	3
2	1	2	2	3
2	1	2	3	3
2	1	1	2	1
2	1	2	2	1
2	1	2	4	3

2	1	2	2	3
3	1	2	3	3
3	1	3	3	3
2	1	1	4	1
2	1	2	2	1
3	2	2	4	4
1	1	1	1	1
3	3	3	3	3
2	1	1	2	3
1	1	1	1	1
3	3	3	4	4
2	1	1	3	3
2	1	2	3	3
1	1	1	2	3
3	2	2	4	3
3	1	3	2	3
3	1	2	3	3
3	1	2	3	3
1	1	1	1	1
1	1	1	2	3
3	3	2	2	3
3	3	3	3	3
2	1	1	1	1
3	2	2	4	3
2	1	2	2	1
3	3	2	4	4
3	2	2	4	4
3	2	2	4	3
2	2	2	3	3
2	1	3	3	3
3	1	2	4	3
3	2	3	3	3
3	1	3	4	3
3	1	2	3	3
2	2	3	3	3
3	1	2	4	3
2	2	2	2	3
3	1	1	4	1
3	3	2	4	3
3	2	3	3	3
3	1	2	4	4
2	1	1	3	3
3	2	2	4	1
2	1	1	2	3
1	1	1	1	1
1	1	1	2	1
3	2	3	4	3
2	1	2	2	3
3	2	3	4	4
2	1	1	2	3

3	2	2	3	3
3	1	2	4	4
3	1	3	3	4
3	1	2	4	1
1	1	1	1	1
2	1	1	2	3
3	2	2	4	4
2	2	2	2	3
3	1	3	2	1
2	1	3	1	1
3	3	3	3	3
2	2	2	3	3
2	3	2	3	3
3	2	2	4	3
2	2	2	2	4
3	2	2	4	3
3	2	3	4	4
3	3	3	4	4
3	2	2	4	4
2	1	2	3	3
3	3	2	4	4
3	3	2	3	3
2	1	2	4	3
3	2	2	3	3
3	1	1	3	3
2	1	1	2	3
1	1	1	1	1
3	2	2	4	4
3	2	3	4	4
1	1	1	2	3
3	2	2	3	3
2	1	1	3	3

1: Toilet use: 1: Dependent. 2: Help. 3: Independent.

2: Going up-down stairs: 1: Dependent. 2: Help. 3: Independent.

3: Urinary sphincter control: 1: Incontinent. 2: Occasional incontinent. 3: Continent.

4: Chair-to-bed transfer: 1: Dependent. 2: Great help. 3: Minimal help. 4: Independent.

5: Walking: 1: Dependent. 2: Independent by electric wheelchair or similar. 3: Help. 4: Independent.

Data Module 9: Level of dependency for each activity of the Barthel index according to the score obtained in the dichotomized classification

Feeding[1]	Bathing[2]	Dressing-undressing[3]	Grooming[4]	Fecal incontinence[5]
1	1	1	1	2
1	1	1	1	1
2	2	2	2	2
2	1	1	1	2
2	2	2	2	2
2	2	2	2	2
2	2	1	1	2
1	1	1	1	1
2	1	2	2	2
2	1	2	1	2
2	1	2	1	2
2	1	1	2	1
1	1	2	2	2
1	1	1	1	1
2	1	2	2	2
2	2	2	2	2
1	1	1	1	1
2	1	2	1	1
2	2	2	1	2
2	1	2	2	2
1	1	1	1	2
2	2	2	2	2
1	1	1	1	2
2	1	2	1	2
2	2	2	2	1
2	1	2	2	1
2	2	2	2	2
2	1	2	1	1
2	1	1	1	1
2	1	2	2	2
1	1	1	1	1
2	1	1	1	2
2	1	1	1	1
1	1	2	1	2
2	2	2	1	2
1	1	2	2	2
1	2	2	2	2
2	1	2	2	2
2	1	1	1	2
2	1	1	1	1
1	1	1	1	2
2	1	2	2	2
1	1	1	1	2
1	1	2	1	2
2	1	2	2	2
2	2	2	1	2

2	1	2	1	2
2	1	2	1	2
2	1	2	2	2
1	1	1	1	2
2	2	2	2	2
1	1	1	1	1
2	2	2	1	2
2	1	2	2	1
1	1	1	1	2
2	1	2	2	2
1	2	2	1	2
1	1	2	1	2
1	1	1	1	1
2	1	2	2	2
2	1	2	1	2
2	1	2	1	2
2	2	2	2	2
1	1	1	1	1
1	1	1	1	1
2	1	2	2	2
2	2	2	2	2
1	1	1	1	2
1	2	2	2	2
1	1	1	1	2
2	2	2	2	2
2	2	2	1	2
2	2	2	2	2
2	1	2	2	2
2	1	2	1	2
2	1	2	2	2
2	1	2	2	2
2	2	2	2	2
1	1	2	2	2
2	1	2	2	2
2	2	2	2	2
1	1	1	1	2
2	2	2	2	2
2	1	2	2	2
2	2	2	2	2
2	1	2	2	2
2	1	1	1	2
2	1	2	2	2
2	1	1	2	2
1	1	1	1	1
1	1	1	1	1
1	1	2	2	2
1	1	1	1	2
2	2	2	2	2
2	1	2	1	2
2	2	2	2	2

2	1	2	2	2
2	2	2	2	2
2	1	2	2	2
1	1	1	1	1
1	1	1	1	2
2	1	2	2	2
2	1	2	1	2
2	2	2	1	2
2	1	2	2	2
2	1	2	2	2
2	1	1	1	2
1	1	1	2	2
2	1	2	2	2
1	1	1	1	2
2	1	2	1	2
2	2	2	2	2
2	1	2	2	2
2	2	2	2	2
2	1	2	1	2
2	2	2	2	1
2	2	2	2	2
2	1	2	2	2
2	1	2	2	2
2	1	2	2	1
1	1	1	1	1
1	1	1	1	1
2	2	2	2	2
2	2	2	2	2
2	1	1	1	1
2	1	2	2	2
2	1	2	2	2

1: Feeding: 1: Dependent or minimal help. 2: Independent.

2: Bathing: 1: Dependent. 2: Independent.

3: Dressing-undressing: 1: Dependent. 2: Independent or minimal help.

4: Grooming: 1: Dependent. 2: Independent.

5: Fecal incontinence: 1: Incontinent. 2: Continent or occasional incontinent.

Data Module 10: Level of dependency for each activity of the Barthel index according to the score obtained in the dichotomized classification

Toilet use[1]	Up- down stairs[2]	Urinary incontinence[3]	Chair-to-bed transfer[4]	Walking on level surfaces[5]
1	1	1	1	1
1	1	1	1	2
2	2	2	1	2
2	1	2	1	2
2	1	2	2	2
2	2	2	2	2
2	1	2	1	2
1	1	1	1	2
2	1	2	1	2
2	2	1	1	2
2	2	2	2	2
1	1	1	1	1
2	2	1	1	2
1	1	1	1	1
2	2	2	2	2
2	2	2	2	2
1	1	1	1	2
1	1	1	2	2
2	1	2	1	2
2	1	2	2	2
1	1	1	2	2
2	2	2	2	2
2	1	1	1	1
2	1	2	2	2
2	1	2	2	2
2	2	2	2	2
2	1	1	2	2
2	2	1	2	2
1	1	1	1	2
2	1	2	1	1
1	1	1	1	1
2	1	1	1	1
1	1	1	1	2
2	2	2	2	2
2	1	2	2	2
2	1	1	2	1
2	1	2	1	2
2	1	1	1	2
1	1	1	1	2
1	1	1	1	2
2	1	2	1	2
2	1	2	2	2
2	1	1	1	1
2	1	2	1	1
2	1	2	2	2

2	1	2	1	2
2	1	2	2	2
2	1	2	2	2
2	1	1	2	1
2	1	2	1	1
2	2	2	2	2
1	1	1	1	1
2	2	2	2	2
2	1	1	1	2
1	1	1	1	1
2	2	2	2	2
2	1	1	2	2
2	1	2	2	2
1	1	1	1	2
2	2	2	2	2
2	1	2	1	2
2	1	2	2	2
2	1	2	2	2
1	1	1	1	1
1	1	1	1	2
2	2	2	1	2
2	2	2	2	2
2	1	1	1	1
2	2	2	2	2
2	1	2	1	1
2	2	2	2	2
2	2	2	2	2
2	2	2	2	2
2	2	2	2	2
2	1	2	2	2
2	1	2	2	2
2	2	2	2	2
2	1	2	2	2
2	1	2	2	2
2	2	2	2	2
2	1	2	2	2
2	2	2	1	2
2	1	1	2	1
2	2	2	2	2
2	2	2	2	2
2	1	2	2	2
2	1	1	2	2
2	2	2	2	1
2	1	1	1	2
1	1	1	1	1
1	1	1	1	1
2	2	2	2	2
2	1	2	1	2
2	2	2	2	2
2	1	1	1	2

2	2	2	2	2
2	1	2	2	2
2	1	2	2	2
2	1	2	2	1
1	1	1	1	1
2	1	1	1	2
2	2	2	2	2
2	2	2	1	2
2	1	2	1	1
2	1	2	1	1
2	2	2	2	2
2	2	2	2	2
2	2	2	2	2
2	2	2	2	2
2	2	2	1	2
2	2	2	2	2
2	2	2	2	2
2	2	2	2	2
2	2	2	2	2
2	1	2	2	2
2	2	2	2	2
2	2	2	2	2
2	1	2	2	2
2	2	2	2	2
2	1	1	2	2
2	1	1	1	2
1	1	1	1	1
2	2	2	2	2
2	2	2	2	2
1	1	1	1	2
2	2	2	2	2
2	1	1	2	2

1: Toilet use: 1: Dependent. 2: Independent or minimal help.

2: Up and down stairs 1: Dependent. 2: Independent or minimal help.

3: Urinary incontinence: 1: Incontinent. 2: Continent or occasional incontinent.

4: Chair-to-bed transfers: 1: Dependent or great help. 2: Independent or minimal help.

5: Walking on level surfaces: 1: Does not walk or use wheelchair. 2: Independent or help.
