# Supplementary material for: Socio-health factors, ability to perform instrumental and basic activities of daily living, and use of assistive mobility devices during the COVID-19 pandemic: Interrelationships and impact on long-term survival
Source: PLoS One. 2025 May 19;20(5):e0318481. doi: 10.1371/journal.pone.0318481 (PMC12088529; doi:10.1371/journal.pone.0318481)
Supplement: S4 Text — (PDF) [file pone.0318481.s004.pdf]

Supplement 4, “Others figures”

Socio-health factors, ability to perform instrumental and basic activities of daily living, and use of assistive mobility devices during the Covid-19 pandemic. Interrelationships and impact on long-term survival.

Authors:

Vicente Martín Moreno. María Inmaculada Martínez Sanz. Irene Sánchez González. Miguel Recuero Vázquez. Sara Guerra Maroto. Miriam Fernández Gallardo. Amanda Martín Fernández. Julia Herranz Hernando. Palma Benítez Calderón. Eva Sevillano Fuentes. Elena Pérez Rico. Laura Calderón Jiménez. Elena Sánchez Rodríguez. Helena Alonso Samperiz. Irene León Saiz. On behalf of GIDO collaborative group (Orcasitas Dependency Research Group). Juana Marcos Guerra.

Address for correspondence:

Vicente Martín Moreno

e-mail: amanvic@hotmail.com

Data behind the figures:

Figures made with the Gephi Graph® 0.10.1 program consist of nodes and lines. Both nodes and lines have been made maintaining proportionality with respect to their weight within the graph. The size of the text describing the node shows the relevance of that node with respect to the whole data.

Figure 1s: Network analysis between socioeconomic factors, level of dependency and use of mobility aids.

Network analysis of the interrelationships observed in the Orcasitas cohort between the mode of living in the community, the availability of assistants for housework and personal care, the level of economic income, the level of functional dependence estimated by the Barthel index and the need to use assistive mobility devices.

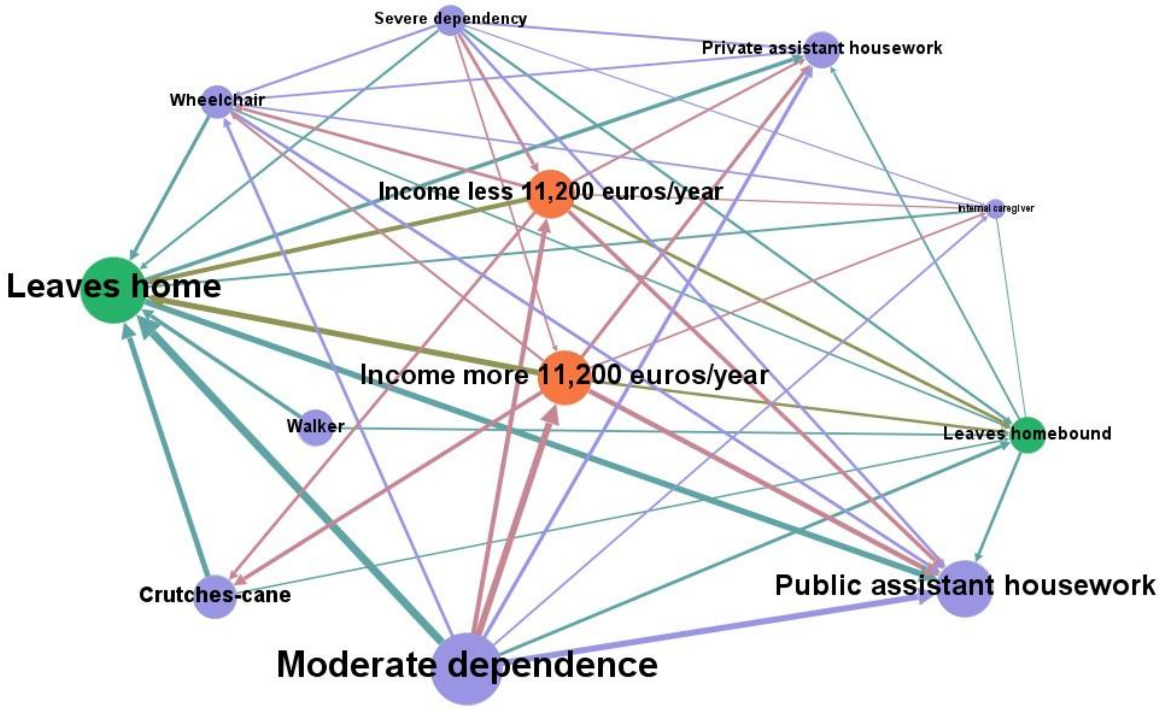

Source, Id, Label, Weight
**Nodes n (%)**
1.- Wheelchair n=41 (32.3%)<br>2.- Crutches-cane n=54 (42.5%)<br>3.- Walker n=45 (35.4%)<br>4.- Income more 11,200 euros/year n=67 (52.8%)<br>5.- Income less 11,200 euros/year n=60 (47.2%)<br>6.- Moderate dependence n=89 (70.1%)<br>7.- Severe dependency n=38 (29.9%)<br>8.- Public assistant housework n=70 (55.1%)<br>9.- Private assistant housework n=45 (35.4%)<br>10.- Internal caregiver n=24 (18.9%)<br>11.- Leaves home n=82 (64.6%)<br>12.- Leaves homebound n=45 (35.4%)<br>13.- Not assistant housework n=33 (26%)

**Networks (n)**
Leaves home – Public assistant housework n=48
Leaves home – Private assistant housework n=30
Leaves home – Internal caregiver n=17
Leaves homebound – Internal caregiver n= 7
Leaves homebound – Private assistant housework n=15
Leaves homebound – Public assistant caregiver n=22
Wheelchair – Leaves home n=27
Wheelchair – Leaves homebound n=14
Crutches-cane – Leaves homebound n=12
Crutches-cane – Leaves home n=42
Walker – Leaves home n=30
Walker – Leaves homebound n=15
Moderate dependence – Income more 11,200 euros/year n=53
Moderate dependence – Income less 11,200 euros/year n=36
Severe dependency – Income less 11,200 euros/year n=24
Severe dependency – Income more 11,200 euros/year n=14
Income more 11,200 euros/year – Leaves home n=46
Income more 11,200 euros/year – Leaves homebound n=21
Income less 11,200 euros/year – Leaves homebound n=24
Income less 11,200 euros/yeat – Leaves home n=36
Moderate dependence – Leaves home n=64
Severe dependency – Leaves homebound n=20
Moderate dependence – Leaves homebound n=25
Severe dependency – Leaves home n=18
Income more 11,200 euros/year – Public assistant housework n=39
Income more 11,200 euros/year – Private assistant housework n=27
Income more 11,200 euros/year – Internal caregiver n=14
Income less 11,200 euros/year – Internal caregiver n=10
Income less 11,200 euros/year – Private assistant housework n=18
Income less 11,200 euros/year – Public assistant housework n=31
Severe dependency – Wheelchair n=18
Moderate dependence – Wheelchair n=23
Severe dependency – Public assistant housework n=22
Severe dependency – Private assistant housework n=17
Severe dependency – Internal caregiver n=9
Moderate dependence – Internal caregiver n=15
Moderate dependence – Private assistant housework n=28
Moderate dependence – Public assistant housework n=48
Income more 11,200 euros/year – Wheelchair n=18
Income less 11,200 euros /year – Wheelchair n=23
Income less 11,200 euros/year – Crutches-cane n=23
Income more 11,200 euros/year – Crutches-cane n=31
Wheelchair – Internal caregiver n=14
Wheelchair – Private assistant housework n=16

Wheelchair – Public assistant housework n=24
Moderate dependence – Crutches-cane n=47
Severe dependency – Crutches-cane n=7
Moderate dependence – Walker n=32
Severe dependency – Walker n=13
Severe dependency – Not assistant housework n=8
Moderate dependence – Not assistant housework n=33
Not assistant housework – Leaves home n=18
Not assistant housework – Leaves homebound n=15

Figure 2s: Covid-19 confinement effect and evolutionary course in the three-year period June 2020 - June 2023.

Effect of confinement on the functional capacity of the persons with functional dependence who make up the Orcasitas cohort. evolutionary course in the three years of follow-up and results in terms of survival-mortality. The specific data are shown in Table 4. Abbreviations: BC: before confinement. AC: after confinement. LH: leaves home. HB: homebound. 3Y: survival at three years.

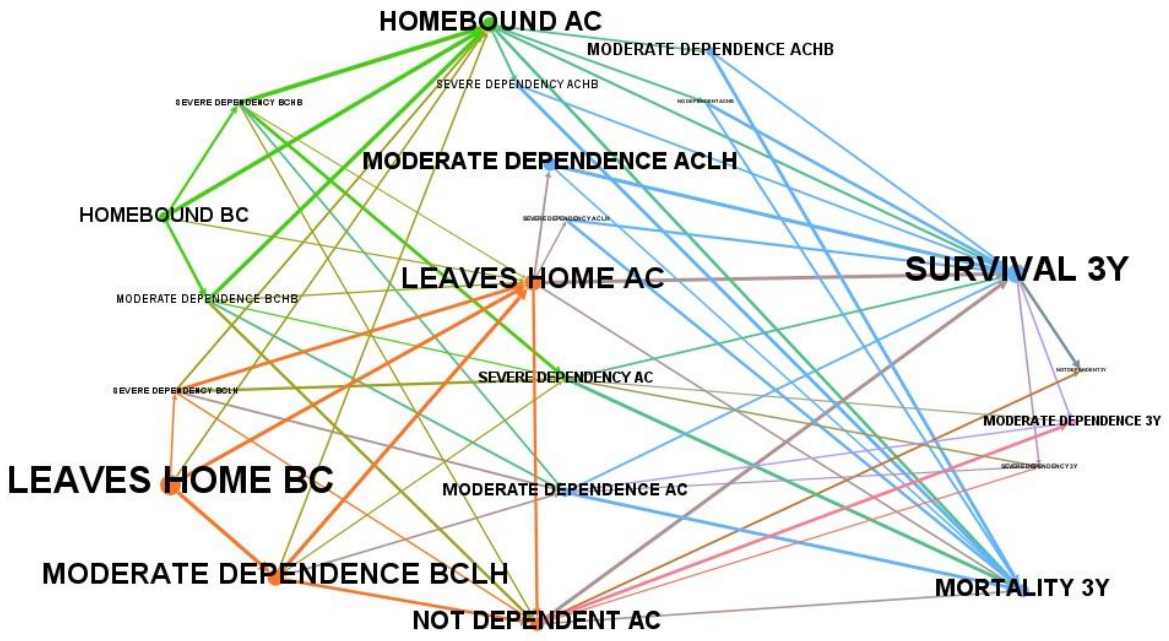

Data behind the figure:

Source. Id. Label. Weight
**Nodes n (%)**
1.- HOMEBOUND BC n=45 (35.4%)<br>2.- LEAVES HOME BC n=82 (64.6%)<br>3.- SEVERE DEPENDENCY BCHB n=20 (15.7%)<br>4.- MODERATE DEPENDENCE BCHB n=25 (19.7%)<br>5.- SEVERE DEPENDENCY BCLH n=18 (14.2%)<br>6.- MODERATE DEPENDENCE BCLH n=64 (50.4%)<br>7.- HOMEBOUND AC n=61 (48%)<br>8.- LEAVES HOME AC n=66 (52%)<br>9.- NOT DEPENDENT AC n=55 (43.3%)<br>10.- SEVERE DEPENDENCY ACHB n=25 (19.7%)<br>11.- MODERATE DEPENDENCE ACHB n=36 (28.3%)<br>12.- SEVERE DEPENDENCY ACLH n=13 (10.2%)<br>13.- MODERATE DEPENDENCE ACLH n=53 (41.7%)<br>14.- NOT DEPENDENT 3Y n=12 (9.4%)<br>15.- SEVERE DEPENDENCY AC n=33 (26%)

16.- MODERATE DEPENDENCE AC n=39 (30.7%)<br>17.- MODERATE DEPENDENCE 3Y n=29 (22.8%)<br>18.- SEVERE DEPENDENCY 3Y n=14 (11%)<br>19.- SURVIVAL 3Y n= 75 (59.1%)<br>20.- MORTALITY 3Y n=52 (40.9%)<br>21.- NOT DEPENDENT ACHB n=10 (7.9%)
**Networks (%)**
HOMEBOUND BC - SEVERE DEPENDENCY BCHB 44.4%
HOMEBOUND BC - MODERATE DEPENDENCE BCHB 55.6%
LEAVES HOME BC - SEVERE DEPENDENCY BCLH 22%
LEAVES HOME BC - MODERATE DEPENDENC BCLH 78%
SEVERE DEPENDENCY BCHB - HOMEBOUND AC 95%
SEVERE DEPENDENCY BCHB - LEAVES HOME AC 5%
SEVERE DEPENDENCY BCHB - NOT DEPENDENT AC 5%
SEVERE DEPEDENCY BCHB - SEVERE DEPENDENCY AC 65%
SEVERE DEPENDENCY BCHB - MODERATE DEPENDENCE AC 30%
MODERATE DEPENDECE BCHB - MODERATE DEPENDENCE AC 36%
MODERATE DEPENDENCE BCHB - SEVERE DEPENDENCY AC 12%
SEVERE DEPENDENCY BCLH - SEVERE DEPENDENCY AC 50%
SEVERE DEPENDENCY BCLH - MODERATE DEPENDENCE AC 33.3%
MODERATE DEPENDENCE BCLH - MODERATE DEPENDENCE AC 28.1%
MODERATE DEPENDENCE BCLH - SEVERE DEPENDENCY AC 12.5%
MODERATE DEPENDENCE BCLH - NOT DEPENDENT AC 59.4%
SEVERE DEPENDENCY BCLH - NOT DEPENDENT AC 16.7%
MODERATE DEPENDENCE BCHB - NOT DEPENDENT AC 52%
SEVERE DEPENDENCY BCHB - NOT DEPENDENT AC 18.5%
MODERATE DEPENDENCE BCHB - HOMEBOUND AC 84%
MODERATE DEPENDENCE BCHB - LEAVES HOME AC 16%
SEVERE DEPENDENCY BCLH - LEAVES HOME 66.7%
SEVERE DEPENDENCY BCLH - HOMEBOUND AC 33.3%
MODERATE DEPENDENCE BCLH - HOMEBOUND AC 23.4%
MODERATE DEPENDENCE BCLH - LEAVES HOME AC 76.6%
HOMEBOUND AC - SEVERE DEPENDENCY ACHB 36.1%
HOMEBOUND AC - MODERATE DEPENDENCE ACHB 29.5%
LEAVES HOME AC - SEVERE DEPENDENCY ACLH 16.7%
LEAVES HOME AC - MODERATE DEPENDENCE ACLH 31.8%
LEAVES HOME AC - SURVIVAL 3Y 74.2%
LEAVES HOME AC - MORTALITY 3Y 25.8%
HOMEBOUND AC - MORTALITY 3Y 57.4%
HOMEBOUND AC - SURVIVAL 3Y 42.6%
NOT DEPENDENT AC - SURVIVAL 3Y 74.5%
NOT DEPENDENT AC - MORTALITY 3Y 25.5%
SEVERE DEPENDENCY ACHB - MORTALITY 3Y 68.2%
SEVERE DEPENDENCY ACHB - SURVIVAL 3Y 31.8%
MODERATE DEPENDENCE ACHB - SURVIVAL 3Y 33.3%
MODERATE DEPENDENCE ACHB - MORTALITY 3Y 66.7%
SEVERE DEPENDENCY ACLH - MORTALITY 3Y 54.5%

SEVERE DEPENDENCY ACLH - SURVIVAL 3Y 45.5%
MODERATE DEPENDENCE ACLH - SURVIVAL 3Y 76.2%
MODERATE DEPENDENCE ACLH - MORTALITY 3Y 23.8%
SURVIVAL 3Y - MODERATE DEPENDENCE 3Y 23.6%
SURVIVAL 3Y - SEVERE DEPENDENCY 3Y 20%
SURVIVAL 3Y - NOT DEPENDENT 3Y 56.4%
NOT DEPENDENT AC - NOT DEPENDENT 3Y 32.3%
HOMEBOUND AC - NOT DEPENDENT ACHB 34.4%
LEAVES HOME AC - NOT DEPENDENT AC 51.5%
SEVERE DEPENDENCY AC - SURVIVAL 3Y 31.8%
SEVERE DEPENDENCY AC - MORTALITY 3Y 68.2%
MODERATE DEPENDENCE AC - MORTALITY 3Y 66.7%
MODERATE DEPENDENCE AC - SURVIVAL 3Y 33.3%
NOT DEPENDENT ACHB - SURVIVAL 3Y 61.9%
NOT DEPENDENT ACHB - MORTALITY 3Y 38.1%
NOT DEPENDENT AC - SEVERE DEPENDENCY 3Y 9.7%
NOT DEPENDENT AC - MODERATE DEPENDENCE 3Y 58.1%
SEVERE DEPENDENCY AC - MODERATE DEPENDENCE 3Y 12.1%
SEVERE DEPENDENCY AC - SEVERE DEPENDENCY 3Y 21.2%
MODERATE DEPENDENCE AC - SEVERE DEPENDENCY 3Y 10.2%
MODERATE DEPENDENCE AC - MODERATE DEPENDENCE 3Y 17.9%
HOMEBOUND BC - HOMEBOUND AC 88.9%
HOMEBOUND BC - LEAVES HOME AC 11.1%
LEAVES HOME BC - LEAVES HOME AC 74.4%
LEAVES HOME BC - HOMEBOUND AC 25.6%

Figure 3s: Relevance of socioeconomic factors and functional dependence on survival.

Network analysis of the influence on survival-mortality of level of dependency. economic level. ability to leave home. homebound status and use or non-use of mobility assistance devices. The role of a King's Fund model.

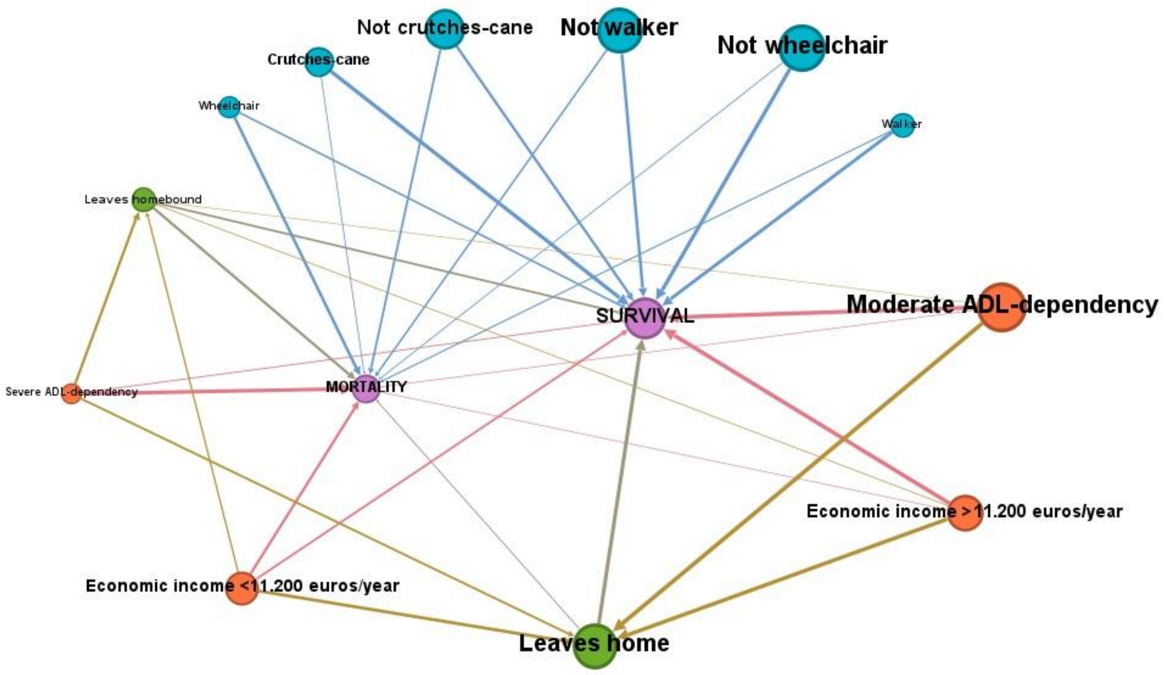

Data behind the figure:

Source. Id. Label. Weight
**Nodes n (%)**
1.- Wheelchair n=41 (32.3%)<br>2.- Crutches-cane n=54 (42.5%)<br>3.- Walker n=45 (35.4%)<br>4.- Income more 11,200 euros/year n=67 (52.8%)<br>5.- Income less 11,200 euros/year n=60 (47.2%)<br>6.- Moderate dependence n=89 (70.1%)<br>7.- Severe dependency n=38 (29.9%)<br>8.- Leaves home n=82 (64.6%)<br>9.- Leaves homebound n=45 (35.4%)<br>10.- Does not use crutches-cane n=73 (57.5%)<br>11.- Does not use walker n=82 (64.6%)<br>12.- Does not use wheelchair n=86 (67.7%)
**Networks (%)**
Economic income greater than 11.200 euros/year – Alive 70.1%
Economic income greater than 11.200 euros/year – Deceased 29.9%

Economic income less than 11.200 euros/year – Alive 46.7%
Economic income less than 11.200 euros/year – Deceased 53.3%
Moderate ADL-dependency – Alive 69.7%
Moderate ADL-dependency – Deceased 30.3%
Severe ADL-dependency – Alive 34.2%
Severe ADL-dependency – Deceased 65.8%
Leaves home – Alive 65.9%
Leaves home – Deceased 34.1%
Homebound – Alive 46.7%
Homebound – Deceased 53.3%
Crutches/cane – Alive 66.7%
Crutches/cane – Deceased 33.3%
Does not use crutches/cane – Alive 53.4%
Does not use crutches/cane – Deceased 46.6%
Walker – Alive 62.2%
Walker – Deceased 37.8%
Does not use walker – Alive 57.3%
Does not use walker – Deceased 42.7%
Wheelchair – Alive 43.9%
Wheelchair – Deceased 56.1%
Does not use wheelchair – Alive 66.3%
Does not use wheelchair – Deceased 33.7%
Moderate ADL-dependency - Leaves home 71.9%
Moderate ADL-dependency – Homebound 28.1%
Severe ADL-dependency – Homebound 52.6%
Severe ADL-dependency - Leaves home 47.4%
Economic income greater than 11.200 euros/year - Leaves home 68.7%
Economic income greater than 11.200 euros/year – Homebound 31.3%
Economic income less than 11.200 euros/year – Homebound 40%
Economic income less than 11.200 euros/year - Leaves home 60%
